# Sulfatide Acts as a Regulatory Molecule Controlling β1 Integrin–STAT5 Signaling and BOLA2-Dependent Apoptotic Pathway in Breast Cancer Cells

**DOI:** 10.3390/ijms262411873

**Published:** 2025-12-09

**Authors:** Jaroslaw Suchanski, Weronika Woldanska, Adam Sciana, Barbara Suchanska, Leszek Moniakowski

**Affiliations:** Department of Biochemistry and Molecular Biology, Wroclaw University of Environmental and Life Sciences, C.K. Norwida 31, 50-375 Wroclaw, Poland

**Keywords:** sulfatide, galactosylceramide sulfotransferase, BOLA2, STAT5, β1 integrin, apoptosis, invasion, breast cancer

## Abstract

Cell membrane glycosylations play a critical role in regulating cell signaling, adhesion, and immune recognition. Abnormal glycosylation is a hallmark of cancer and promotes tumor progression. Sulfatide (SM4), a glycosphingolipid synthesized by galactosylceramide sulfotransferase (CST), is frequently altered in cancers, including breast cancer. Our previous studies identified SM4 as an adhesive molecule that enhances metastasis through interactions with platelets and endothelium; however, its elevated levels increase apoptotic sensitivity and reduce tumorigenicity. Here, we elucidate the molecular mechanisms linking sulfatide metabolism to apoptosis and gene regulation. Using MDA-MB-231 and MDA-MB-468 breast cancer cells with altered CST/SM4 levels, RNA sequencing and functional analyses revealed that overproduction of the CST/SM4 significantly downregulated *BOLA2*, a gene in the CIAPIN1 pathway involved in apoptosis. RT-qPCR and Western blot confirmed an inverse relationship between CST/SM4 and BOLA2. Overexpressing BOLA2 provided resistance to doxorubicin-induced apoptosis, suggesting that SM4-mediated repression of BOLA2 increases apoptotic sensitivity. Luciferase assays showed reduced *BOLA2* promoter activity in SM4-enriched cells. Transcription factor profiling and Electrophoretic Mobility Shift Assay demonstrated that SM4 suppresses STAT5 activation, which directly binds and regulates the BOLA2 promoter. SM4 also altered integrin profiles by upregulating β4/β5 and downregulating β1 subunits. Reintroducing β1 integrin restored STAT5 activation and BOLA2 expression, positioning β1 integrin upstream of STAT5. Collectively, these findings identify a novel sulfatide-dependent β1 integrin–STAT5–BOLA2 pathway controlling apoptosis in breast cancer cells. SM4 suppresses β1 integrin and STAT5-mediated *BOLA2* transcription, promoting apoptosis, while β4/β5 upregulation may facilitate invasion. This pathway represents a potential therapeutic target in breast cancer.

## 1. Introduction

The uniqueness of cell membranes is characterized by the presence of carbohydrate chains carried by glycoconjugates—glycoproteins, proteoglycans, and glycosphingolipids (GSLs)—which are essential for maintaining cellular membrane structure and function [1]. This multitude and diversity in glycosylation patterns are not merely biological variations, but are critical for the precise regulation of cellular processes, underpinning essential functions such as signaling, adhesion, and immune recognition [1]. In cancer, these patterns are often disrupted, contributing to tumor initiation, progression, and metastasis [2]. Although the involvement of the glycan landscape in cancer progression is well recognized [3], the molecular mechanisms underlying the generation of altered glycan structures in cancer cells remain poorly understood. Nevertheless, elucidating these mechanisms appears fundamental to reducing cancer-related mortality.

Sulfated glycosphingolipids, commonly known as sulfolipids (SLs), represent a unique class of anionic amphiphilic GSLs found in various cancers, including those of the brain, kidney, gastrointestinal tract, and breast [4,5]. These molecules are predominantly located in the outer leaflet of the plasma membrane and are involved in cell–cell and cell–matrix interactions [6]. Among SLs, sulfatide (3-O-sulfogalactosylceramide, SGalCer, SM4) is the most extensively studied [6]. It is synthesized by galactosylceramide sulfotransferase (CST) and is highly expressed in the myelin sheath of oligodendrocytes and Schwann cells. Intriguingly, SM4 expression is frequently dysregulated in cancers, including colon, ovarian, gastric, renal, hepatocellular, lung, and breast carcinomas [6,7].

Our previous studies highlighted the essential role of CST and SM4 in the progression of breast cancer (BC). In particular, sulfatides present on the surface of breast cancer cells act as adhesive molecules, triggering their metastatic potential by facilitating interactions between cancer cells and platelets and endothelial cells [7]. Moreover, SM4 acted as an enhancing molecule, increasing the expression of P-selectin in activated platelets [7]. Using immunohistochemistry, we demonstrated that CST expression decreases as tumor malignancy grade increases, with significant differences observed between G1 and G3 breast cancer tumors. Additionally, high CST expression in invasive ductal carcinoma (IDC) is associated with longer overall patient survival. Furthermore, high SM4 levels resulted in increased sensitivity of cancer cells to apoptosis induced by hypoxia and doxorubicin in vitro, and decreased tumorigenicity after transplantation into athymic nu/nu mice [7]. Such a phenomenon has been associated with increased synthesis of SM4, resulting in a reduced level of its precursor, galactosylceramide—a molecule known for its anti-apoptotic properties [7,8,9]. However, our data suggest that this pathway is not exclusive to the increased sensitivity to apoptosis. A comparative gene expression analysis between SM4-positive and SM4-negative BC cells revealed that various genes are differentially expressed in association with apoptosis potential.

Despite SM4′s role in apoptosis regulation, it is also crucial to consider its role in metastasis progression. The metastatic process is closely linked to cell migration and adhesion, primarily mediated by integrins, a class of heterodimeric transmembrane receptors composed of α and β subunits located on the cell surface, mediating adhesion between cells and the extracellular matrix [10]. While certain integrins promote tumor progression, others have been shown to inhibit tumor cell proliferation by inducing apoptosis or cell death [11]. Among them, β1 integrin, which constitutes the largest subgroup of integrins, is aberrantly expressed in breast cancer and is implicated in processes such as epithelial-to-mesenchymal transition (EMT), angiogenesis, metastasis, and therapy resistance [12]. Interactions between β1 integrin and extracellular matrix (ECM) components, such as fibronectin, can protect cancer cells from apoptosis induced by chemotherapeutic agents, including doxorubicin and etoposide [13]. β1 integrin has also been shown to regulate the transcriptional apoptotic machinery, enhancing the expression of anti-apoptotic BCL2, while concurrently downregulating pro-apoptotic genes such as BAX, caspase-3, and p21 [14]. This protective effect is associated with inhibition of caspase-3 activation and PI3K-mediated suppression of mitochondrial cytochrome c release [13]. Recent studies have suggested that sulfatide may influence the expression and activation of certain integrins, although the underlying mechanisms and functional consequences of this regulation are not yet fully understood [15,16].

Given the multifaceted roles of SM4 and its potential regulation of β1 integrin in breast cancer biology, this study aimed to elucidate further its particular role in mediating breast cancer cell resistance and tumorigenicity. We used cell lines with differential SM4 expression to investigate the interplay between sulfatide levels, integrin signaling, and cellular responses to chemotherapy.

## 2. Results

### 2.1. CST Expression Is Correlated with the Down-Regulated Expression of the BOLA2 Gene

**Figure 1:**
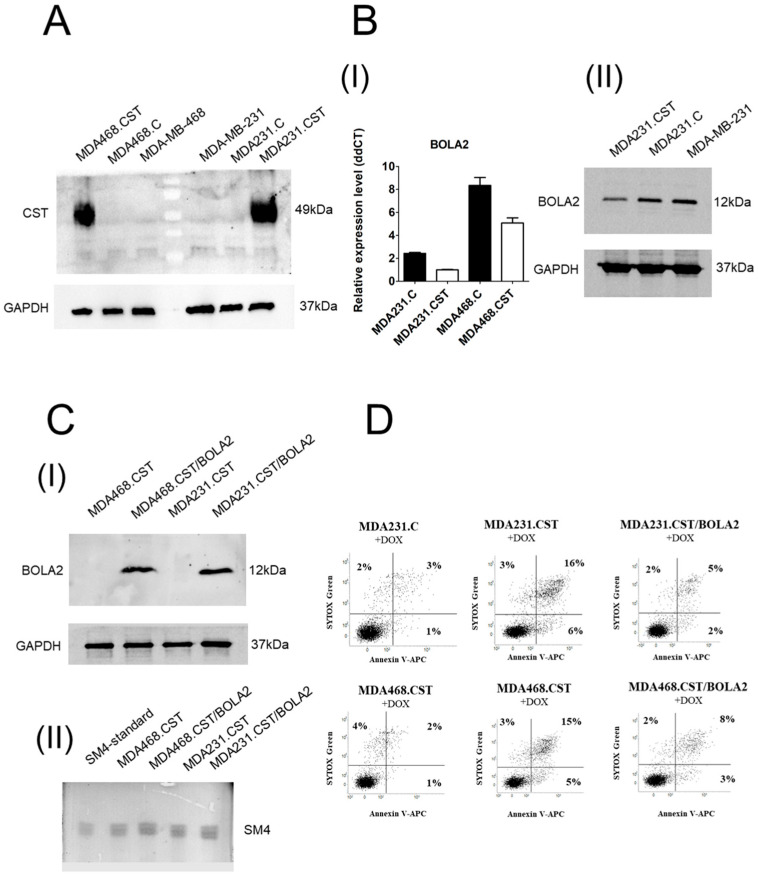
(**A**) Expression of CST protein in parental MDA-MB-231 or MDA-MB-468 cells, control cells transduced with vector alone (MDA231.C or MDA468.C), and cells overexpressing CST (MDA231.CST or M468.CST); Western blotting with anti-human CST antibodies was used to analyze the expression of galactosylceramide sulfotransferase and GAPDH was used as the internal control. (**B-I**) *BOLA2* gene expression was quantified in MDA231.CST and MDA468.CST cells alongside their respective controls using qPCR. mRNA levels were normalized to GAPDH expression with MDA231.CST cells serving as the calibrator. All values are mean ± SD of at least 2 independent experiments, each assayed in triplicate. (**B**-**II**) Expression of BOLA2 protein in parental MDA-MB-231, control cells MDA231.C, and cells overexpressing CST (MDA231.CST). (**C-I**) Level of BOLA2 protein expression in MDA-MB-231 and MDA-MB-468 cells overexpressing simultaneously BOLA2 and CST. (**C-II**) Immunostaining of gangliosides from in MDA-MB-231 and MDA-MB-468 cells simultaneously overexpressing BOLA2 and CST, separated by HP-TLC, with mouse monoclonal antibody against SM4. (**D**) Sensitivity of MDA-MB-231 and MDA-MB-468 cells simultaneously overexpressing BOLA2 and CST with appropriate controls to apoptosis induced by doxorubicin. Cells were grown in the presence of doxorubicin at a concentration of 0.5 μM for 48 h. Cellular response measured by staining with Annexin V and SYTOX Green. Flow cytometry FACS plots show percentage of early apoptotic cells (Annexin V+/SYTOX Green −, lower right) and late apoptotic cells (Annexin V+/SYTOX Green +, upper right). FACS plots display representative results obtained from two independent experiments.

The ability of SM4 to regulate gene expression remains poorly understood. To determine whether the presence of SM4 influences gene expression related to apoptosis potential, we performed Illumina NextSeq 500 sequencing and bioinformatics analyses. Differentially expressed mRNA (DEGs) identified via a volcano plot in RNA from MDA-MB-231 and MDA-MB-468 cells overexpressing human CST and SM4 (Figure 1A), along with appropriate controls, are shown in Figure S1. Comparative gene expression analysis between SM4-positive and SM4-negative breast cancer cells revealed a multitude of differentially expressed genes. Interestingly, high CST expression correlated with downregulation of the *BOLA2* gene, which is involved in apoptosis through the CIAPIN1 pathway [13]. Notably, these changes occurred in both research models (MDA231.CST and MDA468.CST). Those findings were verified by traditional qPCR (Figure 1B-I) and at the protein level for MDA-MB-231 cells (Figure 1B-II). Furthermore, as a proof of concept, we created an MDA-MB-231 and MDA-MB-468 cell model with simultaneously high levels of BOLA2 (Figure 1C-I) and SM4 (Figure 1C-II), leading to increased resistance to doxorubicin–induced apoptosis (Figure 1D). This demonstrates that reduced BOLA2 expression due to the presence of SM4 may be a key factor in making cells more resistant to apoptosis.

### 2.2. Sulfatide Synthesis Does Not Alter Cysteine Redistribution in Cells

**Figure 2:**
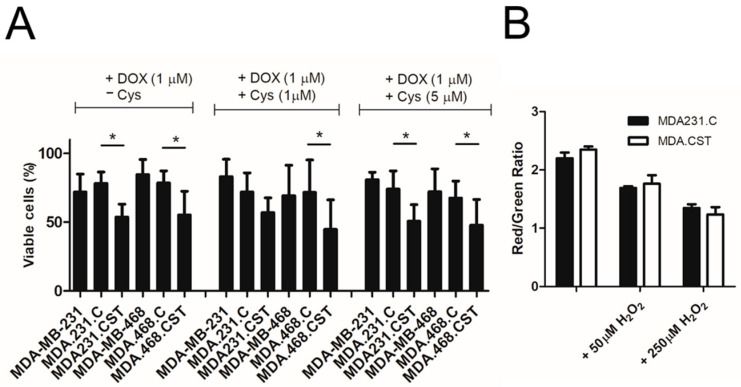
(**A**) Viability of breast cancer MDA-MB-231 and MDA-MB-468 with different SM4 levels exposed to doxorubicin and increased concentrations of cysteine for 48 h. Percentage of viable cells was determined by MTT assay. Data represent the mean ± standard deviation (SD) of 6 replicates from 2 independent measurements. Differences that are statistically significant * *p* < 0.1 (**B**) Lipid peroxidation was measured in the cells treated with or without H_2_O_2_. Fluorescence of the cells were analyzed on a BD FACS Lyric (Becton-Dickinson, Franklin Lakes, NJ, USA) using green (FITC) and red (APC) fluorescence channels. Data, expressed as the FITC/APC fluorescence ratio and indicative of increased green signal with higher lipid peroxidation, represent mean values from two independent cell preparations.

Cysteine biosynthesis through the sulfate assimilation pathway, which involves transporting inorganic sulfate into the cell, may be disrupted in cancers with elevated SM4 levels. This disruption occurs because sulfur is redirected toward conversion to PAPS (which, as mentioned earlier, acts as a donor for sulfotransferases like CST). It was important to determine whether SM4 directly inhibits BOLA2 expression or if the lack of cysteine as a sulfur source is more significant. Supplementing with cysteine did not significantly change the viability of breast cancer cells treated with doxorubicin. Using gain-of-function cellular models, it was demonstrated that all analyzed SM4-rich breast cancer cells were more sensitive to the cytotoxic effects of doxorubicin than cells unable to synthesize this GSL (Figure 2A). Notably, the sensitivity of the control MDA231.C and MDA468.C cells to anti-cancer drugs was the same as that of the respective parental wild-type cells. Additionally, lipid peroxidation, a process linked to non-apoptotic cell death, remained unchanged, as indicated by the lipid peroxidation assay (Figure 2B), suggesting that the effects are specific to apoptotic pathways, as also confirmed by our previous studies [5,6,9]. The exact role of sulfatide within ferroptosis through BOLA2 regulation needs however to be further investigated.

### 2.3. SM4 Is Responsible for Changes in BOLA2 Gene Expression at the Transcriptional Level

**Figure 3:**
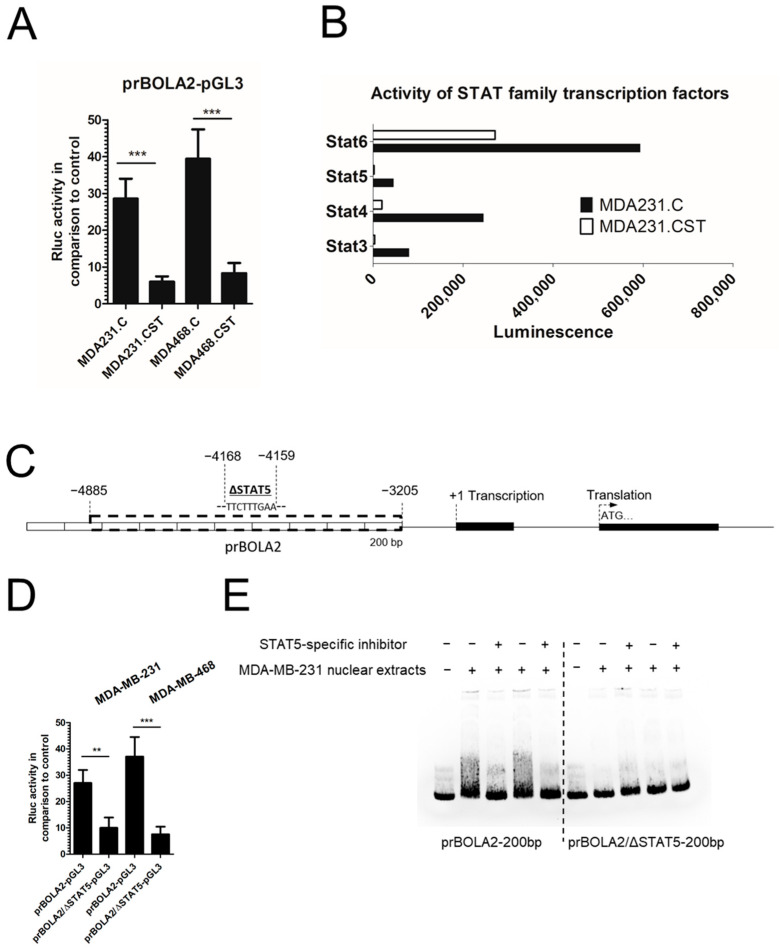
(**A**) Transcriptional activity of the *BOLA2* promoter fragment cloned into the pGL3 Basic Vector (prBOLA2-pGL3) after transfection into breast cancer cells MDA231.C and MDA468.C (lacking SM4) compared to MDA231.CST and MDA468.CST cells with high SM4 levels. All values are mean ± SD of two independent transfection experiments, each assayed in five technical replicates. Statistically significant differences (*** *p* < 0.001). (**B**) The activity of the STAT family was assessed based on each factor’s ability to bind specific DNA sequences in cells with high or low sulfatide levels, using the Combo TF Activation Profiling Plate Array-192 (Signosis). Values are reported from a single experiment with no technical or biological replicates. (**C**) Mutational analysis of STAT5 binding site in the BOLA2 gene promoter. A schematic representation of the *BOLA2* promoter (prBOLA2) shows a functional and deleted STAT5 binding site, which is indicated by the sequence of the sense strand of the DNA. (**D**) Luciferase activities of prBOLA2/ΔSTAT5 -pGL3 deletion mutant, following transfection into the MDA-MB-231 cell line, were measured. Promoter activities were assessed using the Dual-Luciferase Reporter Assay System (Promega, Madison, WI, USA). The bars represent the average luciferase activities compared to the control pRL-TK vector. All values are expressed as mean ± SD from two independent experiments, each assayed in five technical replicates. Statistically significant differences (** *p* < 0.01; *** *p* < 0.001) (**E**) Binding of nuclear proteins from MDA-MB-231 cells to STAT5 response element fragment, analyzed by EMSA in the presence (+) and absence (−) of nuclear lysate and STAT5-specific inhibitor, respectively.

Changes in the expression of *BOLA2* mRNAs in BC cells with varying levels of SM4 suggest that SM4 may influence the transcription of this gene. To investigate this, the promoter region of the BOLA2 gene, approximately 1000 bp long and designated as prBOLA2, was cloned into the pGL3 basic vector (prBOLA2-pGL3). A dual-luciferase reporter assay system was then used to measure its activity in BC cell lines with different levels of CST expression. The activity of the *BOLA2* promoter was higher in MDA231.C and MDA468.C cells lacking SM4 compared to MDA231.CST and MDA468.CST cells with high SM4 levels (Figure 3A). The cis-regulatory effects of SM4 on the cell surface likely relate to the activation or inhibition of signaling pathways, as well as the regulation of transcription factors that directly interact with the BOLA2 promoter. To explore this, the activity of 192 different transcription factors was examined in high- and low-sulfatide level cell lines using the Combo TF Activation Profiling Plate Array-192 (Signosis, Santa Clara, CA, USA) (Figure S3). The activity of transcriptional factor protein was determined based on each factor’s ability to bind specific DNA sequences, producing luminescence. Using criteria such as statistical significance and relevance to stress found in the literature, a key finding was the decreased activity of a few STAT family factors in cell lines with high sulfatide levels (Figure 3B). Based on these results, an in silico analysis was performed to identify potential transcription factor binding sites that could affect BOLA2 promoter activity in breast cancer cells. The Genomatix MatInspector software version 2.7 detected numerous transcriptional elements within each analyzed promoter sequence. Notably, a putative STAT5 transcription factor binding site was identified within the *BOLA2* gene promoter region (Figure 3C).

### 2.4. STAT5 Induces BOLA2 Gene Transcription

Based on in silico analysis, we found that STAT5 interacts with sequences located at positions (−4159/−4168) of the prBOLA2, and these binding sites are designated as ΔSTAT5. Therefore, to demonstrate that STAT5 is directly involved in regulating the *BOLA2* gene, prBOLA2 mutants with deletions of the STAT5 binding sites were generated through site-directed mutagenesis. After cloning into the pGL3 Basic vector, the reporter plasmids called prBOLA2/ΔSTAT5 -pGL3 were tested for promoter activity following transfection into MDA-MB-231 or MDA-MB-468 cells. It was found that deletion of ΔSTAT5 significantly reduced prBOLA2 activity (Figure 3D). To confirm that STAT5 is the main nuclear protein in breast cancer cells binding to the prBOLA2 fragment, MDA-MB-231 cells were treated with a STAT5-specific inhibitor, and nuclear extracts from these cells were subsequently used in electrophoretic mobility shift assays (EMSA) to assess interactions with a 200 bp fragment of the prBOLA2 promoter, either containing (prBOLA2-200bp) or lacking (prBOLA2/ΔSTAT5-200bp) the STAT5 binding site. The results showed that inhibiting STAT5 abolished the binding of nuclear proteins from inhibitor-treated MDA-MB-231 cells to the prBOLA2-200 bp fragment. In contrast, strong binding was seen in nuclear extracts from untreated cells (Figure 3E). Furthermore, a fragment lacking the STAT5 binding site exhibited markedly reduced interaction with all of the MDA-MB-231 nuclear lysates, further supporting the functional importance of this region in mediating interaction with STAT5 (Figure 3E).

### 2.5. SM4 Regulates Integrin Expression: Implications for Cellular Invasion and Signal Transduction

STAT5 is known to be regulated by the β1 subunit of integrin [9], while sulfatides have been shown to influence the expression of specific integrins in cancer cells, especially integrin αV [10]. Therefore, using the Integrin Antibody Sampler Kit (Cell Signaling Technology), we compared the levels of various integrin subunits in MDA231.CST relative to their respective control. Interestingly, integrin subunits—specifically, β4 and β5—were found to be upregulated, while the β1 subunit showed notable downregulation (Figure S2). These expression patterns suggest different functional roles for these subunits, with β4 and β5 possibly enhancing cellular invasiveness, and β1 likely involved in regulating intracellular signaling pathways (see below).

**Figure 4:**
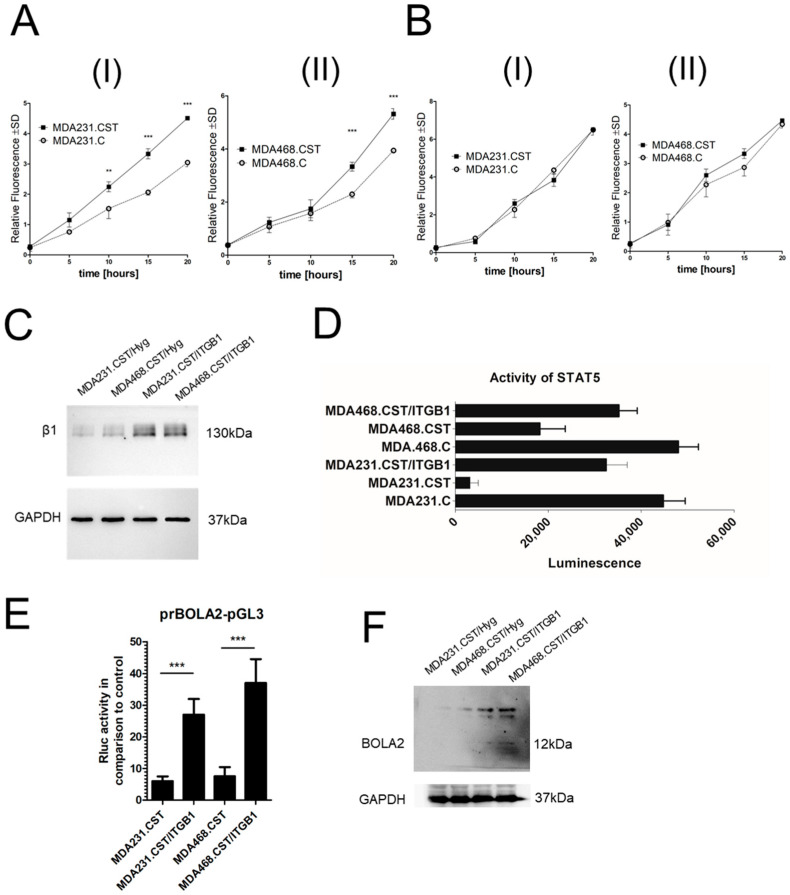
(**A**) Invasiveness or (**B**) Migratory capability of MDA231.CST (**A-I**,**B-I**) and MDA468.CST (**A-II**,**B-II**) cells compared to appropriate controls. Data are presented as mean ± SD. Bonferroni multiple comparison test, ** *p* < 0.01. Results are based on three independent biological experiments, each assayed in two technical replicates. (**C**) Expression of ITGB1 protein in cells overexpressing CST (MDA231.CST and MDA468.CST), control cells transduced with vector alone (MDA231.CST/Hyg and MDA468.CST/Hyg) and cells exhibiting simultaneous overexpression of CST and ITGB1(MDA231.CST/ITGB1 and MDA468.CST/ITGB1) (**D**) The activity of the STAT5 was assessed based on STAT5 ability to bind specific DNA sequences using the Combo TF Activation Profiling Plate Array-192 (Signosis). Results are based on two independent biological experiments, each assayed in three technical replicates. (**E**) Transcriptional activity of the *BOLA2* promoter fragment cloned into the pGL3 Basic Vector (prBOLA2-pGL3) after transfection into breast cancer cells: MDA231.CST, MDA231.CST/ITGB1, MDA468.CST and MDA468.CST/ITGB1. All values are mean ± SD of at least two independent transfection experiments, each assayed in five technical replicates. Statistically significant differences (*** *p* < 0.001) (**F**) Expression of BOLA2 protein in MDA231.CST/ITGB1 and MDA231.CST/ITGB1cells compared to appropriate controls.

To clarify the role of sulfatide on the surface of cells in breast cancer progression, migration and invasion assays were conducted using tumor cells that produce varying amounts of sulfatide. Our data indicate that SM4 on the surface of MDA-MB-231 and MDA-MB-468 directly affects the malignant properties of BC cells by significantly increasing their invasiveness, thereby promoting the movement of cancer cells into the tumor stroma (Figure 4(A-I,A-II)). A statistically significant increase in invasiveness was observed in BC cells overproducing SM4 compared to control cells at the 15th and 20th hours of the experiment in both cell models (*p* < 0.0001, respectively; Bonferroni multiple comparison test). However, when comparing the migration abilities of both cell lines, no differences were detected in the in vitro migration assay (Figure 4(B-I,B-II)).

To demonstrate that integrin β1 directly influences STAT5 activity, MDA231.CST and MDA468.CST cells were used to create a model co-expressing integrin β1 and sulfotransferase. Transducing these cells with the pRRL-CMV-ITGB1-IRES-Hyg expression vector containing ITGB1 cDNA resulted in a cell population with restored integrin β1 expression, while sulfatide levels remained consistently high (Figure 4C). It was found that increased β1 expression enhances STAT5 activity in MDA231.CST and MDA468.CST cell lines (Figure 4D), which in turn leads to higher promoter activity of *BOLA2* in these cells (Figure 4E). To confirm that changes in integrin β1 expression directly affect BOLA2 levels, BOLA2 expression was analyzed at the protein level in MDA-MB-231 cells with high integrin β1 and sulfatide levels. As expected, the restored expression of integrin β1 caused a significant increase in BOLA2 protein level (Figure 4F).

## 3. Discussion

The impact of SM4 on cancer cell behavior after entering the bloodstream is clearly negative, as SM4 acts as a malignancy-related adhesive molecule that promotes metastasis [7,17,18]. However, its role in the development of primary tumors remains unclear. On the one hand, we demonstrated that CST expression decreases with increasing tumor malignancy grade, with significant differences observed between G1 and G3 tumors. Additionally, high CST expression in IDC cells is associated with longer overall survival for patients, suggesting that higher CST levels may indicate a better prognosis [6]. A clinical study involving IDC patients supports our observation that, in primary tumors, cancer cells rich in sulfatides are more likely to undergo apoptosis triggered by microenvironmental stressors such as hypoxia. This is because increased synthesis of this glycolipid reduces the levels of its precursor, GalCer, an anti-apoptotic molecule [6,12]. On the other hand, our data presented in this study indicate that SM4 on the surface of MDA-MB-231 and MDA-MB-468 cells directly influences the malignant properties of breast cancer cells by significantly increasing their invasiveness, thereby facilitating cancer cell movement into the tumor stroma.

The increase in invasiveness in cells with elevated levels of SM4 is most likely due to changes in the cell membrane structure associated with the formation of sulfatide clusters, commonly referred to as ”lipid rafts.” The proteins contained within these lipid rafts can modulate cell invasiveness, and available literature data suggest the potential involvement of SM4 in tumor cell invasiveness through regulation of integrins [11,12], which are a superfamily of cell adhesion receptors that bind to extracellular matrix ligands, cell-surface ligands, and soluble ligands [13]. Integrins are essential for cell migration and invasion, not only because they directly mediate adhesion to the extracellular matrix but also because they regulate intracellular signaling pathways that, in turn, control cytoskeletal organization, force generation, and survival [14]. They are transmembrane αβ heterodimers, and at least 18 α and 8 β subunits are known in humans, forming 24 heterodimers [19]. Increased expression levels of integrins αvβ3 are closely associated with increased cell invasion and metastasis [20]. Integrin α6 expression is also significantly upregulated in numerous carcinomas, including breast cancer [21]. Expression of integrin α6β4 enhances tumor cell invasiveness and metastasis, especially in breast carcinomas [22]. It has been shown that SM4 enhances cell invasiveness by activating Src kinase and increasing integrin αVβ3 expression [16]. Our data indicate that SM4 present on the surface of BC cells does not affect mRNA expression levels of specific integrins. However, SM4 can modulate the quantity of integrins through post-translational regulation. Interestingly, several integrin β subunit family members, namely β3, β4, and β5, were elevated, which could result in increased invasiveness. However, a particularly intriguing observation was the decreased expression of the β1 subunit, which is the most common subunit and has emerged as a key mediator in cancer, influencing various aspects of cancer progression, including cell motility, adhesion, migration, proliferation, differentiation, and chemotherapy resistance [23]. The role of β1 in this context is probably not associated with changes in cell invasiveness but rather with its impact on the signaling pathways, which will be discussed later.

**Figure 5:**
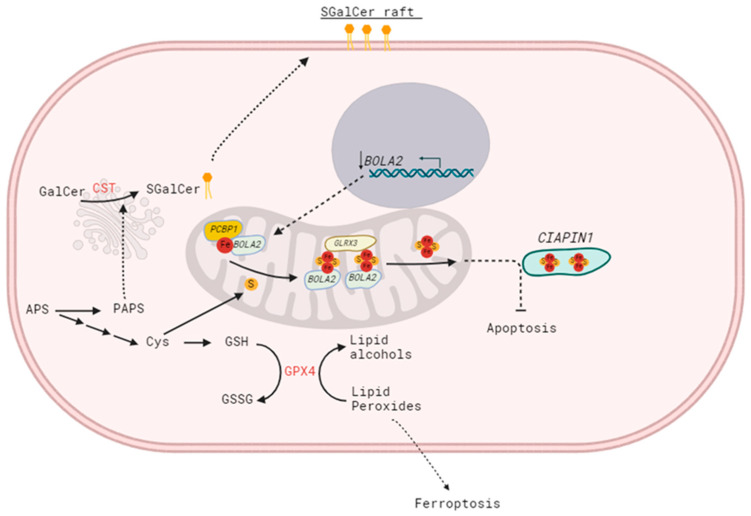
Proposed pathway for sulfur assimilation into cysteine or sulfatide, and the role of BOLA2 in mammalian cytosolic iron-sulfur cluster trafficking as a potential key factor in breast cancer apoptosis.

As mentioned above, the accumulation of sulfatides in breast cancer cells correlates with increased sensitivity to doxorubicin-induced apoptosis [7] by altering metabolism and reducing the amounts of GalCer, which acts directly as an anti-apoptotic molecule [8]. However, our data suggest this is not the only pathway leading to increased sensitivity to apoptosis. The ability of SM4 to regulate gene expression remains a poorly understood phenomenon, but a comparative gene expression analysis between SM4-positive and SM4-negative BC cells revealed that various genes are differentially expressed in association with apoptosis potential. Among them, high CST expression correlated with down-regulated expression of the *BOLA2* gene, known to be involved in apoptosis through the CIAPIN1 pathway [24]. The role of the *BOLA2* gene in the carcinogenesis of breast cancer cells, as well as the underlying mechanism, remains poorly understood. Interestingly, BOLA2 forms a distinct iron chaperone complex with Glx3 for the cytosolic [2Fe-2S] cluster, which can be directly transferred to the Fe-S protein Ciapin1 (cytokine-induced apoptosis inhibitor-1) in vitro. Incorporation of [2Fe-2S] into Ciapin1, a well-known regulator of apoptosis in many different cell types, is crucial for its activity [25]. The sulfur source needed to form the [2Fe-2S] clusters is unclear, possibly provided by the cytosolic isoform of the cysteine desulfurase NFS1 or exported from the mitochondrial ISC (iron–sulfur cluster) pathway [26]. Changes in BOLA2 expression suggest an alternative mechanism for sensitizing cells to apoptosis by blocking the proper redistribution of sulfur, as shown in Figure 5. Although cysteine is the least abundant amino acid in the cell, evidence describes it as one of the most important amino acids for cell survival and growth [27]. Cysteine biosynthesis through the sulfate assimilation pathway, which involves the transport of inorganic sulfate into the cell, could be disrupted in cancers with increased amounts of SM4, because the sulfur redistribution shifts toward conversion to PAPS (as mentioned earlier, PAPS serves as a donor for sulfotransferases like CST).

In this context, it was essential to determine whether SM4 directly inhibits BOLA2 expression or if the absence of cysteine as a sulfur source is more significant. We found that (1) adding cysteine to the culture did not significantly affect the sensitivity of BC cells with SM4 to apoptosis induced by doxorubicin, and (2) overproduction of sulfatide does not adversely affect the availability of cysteine. This suggests that sulfur availability is not the decisive factor in determining the sensitivity of cells with high sulfatide content to chemotherapeutic agents. This also suggests a direct involvement of the SM4 in the regulation of BOLA2 expression, especially since we observed a significant reduction in *BOLA2* promoter activity in MDA231.CST cells. The cis-regulatory properties of SM4 present on the cell surface are most likely related to the activation or deactivation of signaling pathways, along with the associated regulation of transcription factors interacting directly with the *BOLA2* promoter. Therefore, the activity of 192 different transcription factors was verified, with a particularly interesting decrease in the activity of signal transducers and activators of transcription (STAT) family factors in breast cancer cells with a large amount of SM4. To date, seven members of the STAT family have been identified in mammals [28]. While some cytokines and growth factors are capable of activating multiple STAT proteins, certain STAT proteins are activated with a high degree of specificity by particular kinases [29]. The binding site for STAT5 is present in the promoter for the BOLA2 gene, suggesting a potential involvement in its activity. This protein is activated in response to a diverse number of cytokines, growth factors, and hormones. Upon activation, which occurs following ligand-receptor binding, STAT5 proteins undergo dimerization, translocate to the nucleus, and bind to the promoters of specific target genes [30]. In this proposal, STAT5 was identified as a key regulatory protein driving the overexpression of BOLA2 in breast cancer cells exhibiting a malignant, mesenchymal-like phenotype. This hypothesis was substantiated by functional studies demonstrating the role of STAT5 in the transcriptional upregulation of *BOLA2*. Specifically, pharmacological inhibition of STAT5 activity led to a marked reduction in BOLA2 expression, which was associated with increased sensitivity to apoptosis. Although the data presented here (EMSA, STAT5 inhibition, reporter assays) provide indirect but supportive evidence for STAT5-mediated regulation of BOLA2, direct demonstration of STAT5 binding to the BOLA2 promoter remains to be established.

**Figure 6:**
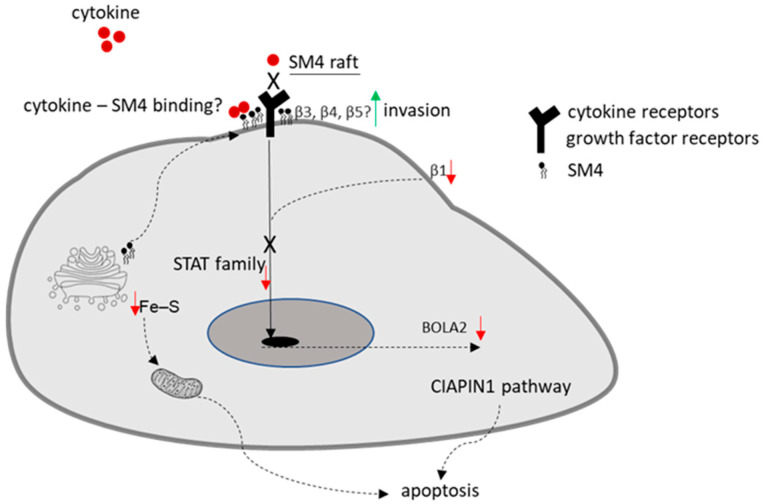
Potential mechanism behind the effect of membrane-anchored SM4 on the invasive properties of BC cells and the signal transduction pathways that lead to apoptosis.

While the cytosolic SM4-dependent signaling pathway has been partially described before, the role of the plasma membrane in this regulation remains unclear. Sulfatides, in contrast to gangliosides, neutral glycolipids, and phospholipids, are bound by various chemokines such as MCP-1/CCL2, IL-8/CXCL8, SDF-1α/CXCL12, MIP-1α/CCL3, and MIP-1β/CCL4 [31]. Since SM4 is a component of lipid rafts, and chemokine receptors are part of these specific microdomains found in the cell membrane, it is hypothesized that they may modulate chemokine signal transduction. SM4 not only binds chemokines but also can regulate cytokine expression in human lymphocytes and monocytes [32]. Incubation of these cells, previously activated with phytohemagglutinin with sulfatide, resulted in a decrease in IL-6 production. Conversely, in cells activated with LPS, sulfatide reduced the secretion of IL-1β and IL-10. Subsequent studies have shown that the effect of sulfatide depends on the type of fatty acid in the molecule. The sulfatide containing palmitic acid, abundant in the cells of the pancreatic islets of Langerhans, was discovered to be the most potent inhibitor of cytokine production, including IL-1β, TNF-α, MIP-1α, and IL-8 [33]. In the brain, SM4 derived from myelin stimulated cytokine production by lymphoid cells with the capacity to initiate an immune response [34]. There is no experimental evidence for this phenomenon; however, there is a high probability that SM4 present in the cell membrane, in lipid rafts, may compete with cytokine receptors and thus inhibit the activity of cytokine-dependent transcription factors such as members of the STAT family, Figure 6. Another regulatory pathway for this TF family involves integrin β1 [35], and its regulation may be dependent on SM4 since, as previously mentioned, integrin β1 is likely inactive in cell lines with high levels of SM4. The restored expression of integrin β1 resulted in a significant upregulation of both *BOLA2* mRNA and protein levels. However, the underlying mechanism by which sulfatide levels influence BOLA2 regulation through the β1 integrin/STAT5 signaling pathway remains undefined and requires further elucidation.

## 4. Materials and Methods

### 4.1. Cell Lines and Culture Conditions

Human breast cancer MDA-MB-231 and MDA-MB-468 cell lines were purchased from American Type Culture Collection (ATCC). They were cultured in α-minimum essential medium (α-MEM) supplemented with 10% fetal bovine serum (FBS, Cat. No. S181H, Biowest, Riverside, MO, USA), 2 mM L-glutamine, 100 U/mL streptomycin, and 0.1 mg/mL penicillin (complete α-MEM). For the EMSA experiments, MDA-MB-231 breast cancer cells were treated with the STAT5 inhibitor (catalog no. 573108-M, Merck, Darmstad, Germany), reconstituted in DMSO at a 25 mM stock concentration, with an initial dose of 10 μM (24 h) followed by a 5 μM maintenance dose (48 h).

### 4.2. Cell Survival Assay

The viability of the cells was measured using an MTT assay. Briefly, 5 × 10^3^ cells per well were seeded in a 96-well plate (Nunc, Rochester, NY, USA) and incubated with 1 µM doxorubicin hydrochloride (DOX) along with increasing concentrations of cysteine (Cat. No. 168149, Merck) for 48 h. Afterward, the medium was discarded, and the cells were incubated with MTT solution (0.5 mg/mL, Merck) for 4 h at 37 °C. Following this, the MTT solution was removed, and 200 µL of DMSO (Chempur, Piekary Śląskie, Poland) was added for 15 min at room temperature. The optical density was then measured at 570 nm.

### 4.3. Purification of Gangliosides, Thin-Layer Chromatography (TLC), and TLC Binding Assay

Gangliosides were purified as described previously [7,36]. Glycolipids were extracted from 10^8^–10^9^ cells using the chloroform–methanol extraction method. HP-TLC was performed on Silica Gel 60 high-performance TLC (HPTLC) plates (Merck). For ganglioside/sulfatide separation, a chloroform: methanol: 0.2% CaCl_2_ solvent system (60:40:9, *v*/*v*/*v*) was used. Sulfatide was detected by TLC binding assay as described previously [8].

### 4.4. RNA-Sequencing and RT-qPCR

Total RNA was extracted using the RNeasy Mini Kit (Cat. No. 74104, Qiagen, Hilden, Germany) following the manufacturer’s instructions. RNA purity and concentration were measured with the Nanodrop DS-11 FX (Denovix, Wilmington, DE, USA). RNA library preparation and transcriptome sequencing were performed by Novogene (Cambridge, UK). Genes with an adjusted *p*-value  <  0.05 and |log2 (FoldChange)|  >  0 were considered differentially expressed. To validate the RNA-Seq data, the expression of selected candidate genes was assessed with RT-qPCR. Briefly, 1 μg of total RNA was reverse transcribed into complementary DNA (cDNA) using the High-Capacity cDNA Reverse Transcription Kit (Thermo, Oxford, UK) in a 20-μL volume. The relative mRNA levels were measured by qPCR with the EvaGreen dye-based detection system (Cat. no. 31000, Biotium, Fremont, CA, USA) according to the manufacturer’s instructions, using an iQ5 Optical System (Bio-Rad, Hercules, CA, USA). GAPDH served as the internal control. Primer sequences are listed in Supplementary Table S1.

### 4.5. SDS-PAGE and Western Blot

RIPA buffer containing Protease Inhibitor Cocktail (Merck, Darmstadt, Germany) was used for total protein isolation. The protein content in the lysate was measured using the Bicinchoninic Acid Kit (Merck). A 4-fold concentrated Laemmli buffer with beta-mercaptoethanol was then added to the samples, which were boiled at 95 °C for 5 min. Samples containing 40 μg of protein per well were separated by SDS-PAGE electrophoresis and transferred onto 0.45 µm nitrocellulose membranes (Cytiva, Uppsala, Sweden) using the Semi-Dry Trans-Blot Turbo system (Bio-Rad). Next, the membrane was blocked with TBS buffer containing 0.5% skimmed milk. After blocking, the membrane was incubated overnight at 4 °C with primary antibodies: anti-BOLA-2 (Cat. No. A305-890A, Thermo), anti-CST (Cat. No. NBP1-85431, Novus Biologicals), and mouse monoclonal anti-GAPDH antibody (Cat. No. NB300-221, Novus Biologicals). Following washes, the membranes were incubated at room temperature for 1 h with HRP-conjugated goat polyclonal secondary antibodies targeting rabbit (Cat. No. P0448, Dako) or murine (Cat. No. 115-035-003, Jackson ImmunoResearch, West Grove, PA, USA) immunoglobulins. After additional washing, they were exposed to substrate to visualize HRP complexes (Cat. No. 34094, Thermo). Chemiluminescent detection was performed using ChemiDoc Touch (Bio-Rad). Protein levels were quantified relative to GAPDH expression.

### 4.6. Dual-Luciferase Reporter Assay

The promoter region of the *BOLA2* gene was cloned by amplification of the genomic sequence by PCR using total DNA purified from MDA-MB-231 cells as a template and forMluI-prBOLA2 and revNheI-prBOLA2 primers (Table S1) as follows: 35 cycles of denaturation (95 °C for 20 s), annealing (55 °C for 20 s) and extension (72 °C for 1 min). The generated *BOLA2* promoter was cloned into the corresponding sites of the pGL3 Basic Vector (Cat. No. E1751, Promega) and named: pGL3-BOLA2/LUC.

To assess promoter activity, cells were processed using Promega’s Dual-Luciferase Reporter Assay System. In brief, cells were cultivated to approximately 70% confluence in 6-well plates (Nunc) and then co-transfected with a pGL3 promoter-containing Firefly luciferase reporter vector (2 μg) and a control *Renilla* luciferase-expressing pRL-TK vector (2 μg) with polyethylenimine (PEI) reagent. Following a 48 h incubation period, the cells were lysed and then assayed for luciferase activity. Firefly luciferase and *Renilla* luciferase signals were sequentially measured on a luminescence microplate reader (Tecan, Männedorf, Switzerland). The signal integration time was set to one second per well. Mock-transfected cells and empty wells were included to evaluate the background luminescence. The relative luciferase activity was then normalized to the *Renilla* luciferase activity. Each experiment was performed in three independent biological replicates.

### 4.7. Nuclear Extracts and Electrophoretic Mobility Shift Assay (EMSA)

The purification of nuclear proteins was carried out using a commercially available NE-PER Nuclear and Cytoplasmic Extraction Kit (Cat. No.78833, Thermo), following the manufacturer’s instructions. The purity of non-nuclear and nuclear fractions was subsequently determined using specific protein markers, namely GAPDH and histone H3, respectively, Figure S4. Biotin end-labeled double-stranded oligonucleotides were then generated using PCR, using biotinylated primers at the 5′ end (Table S1), followed by purification of the obtained products. The binding reactions (including 5 μg of nuclear extract protein, 1 μg of poly (dI-dC), 2 nM of biotin-labeled DNA, and 10 mM Tris buffer, pH 7.5, 50 mM KCl, 5 mM MgCl_2_, 1 mM dithiothreitol, 0.05% Nonidet P-40, and 2.5% glycerol) were carried out at 23 °C for 20 min. To separate bound oligonucleotides from free probe, the samples were electrophoresed on a 6% Tris-borate-EDTA gel at 100 V for 1 h in a 100 mM Tris-borate-EDTA buffer and transferred to a nylon membrane. The biotin-labeled DNA was detected with a LightShift chemiluminescent electrophoretic mobility shift assay kit (Cat. No. 20148, Thermo).

### 4.8. Apoptotic Assay

The cells were seeded in 6-well plates at a density of 5  ×  10^5^ cells/mL. The following day, the cells were treated with DOX for 48 h. Apoptosis was measured by staining cells with Annexin V conjugated with red-laser-excited allophycocyanin (APC) and SYTOX Green using the Dead Cell Apoptosis Kit (Cat. No. V13242, Thermo, Oxford, UK). Fluorescence was measured on a BD FACSLyric (Becton-Dickinson, Franklin Lakes, NJ, USA), and data were analyzed using the BD FACSuite™ Software v1.5.0.925 (Becton-Dickinson). The percentage of Annexin V APC-positive cells corresponded to cells in early apoptosis, whereas Annexin V APC and SYTOX Green-positive cells corresponded to cells in late apoptosis.

### 4.9. Lipid Peroxidation Assay

The cells (10^6^) were seeded in 6-well plates (Nunc, Rochester, NY, USA). The following day, lipid peroxidation was determined using a lipid peroxidation assay kit (ab243377, Abcam, Cambridge, UK) in cells treated with 50 µM H_2_O_2_ or 250 µM H_2_O_2_ (30 min). In brief, a ratiometric lipid peroxidation sensor was added to the cells and incubated for 30 min. After 3 washing steps with HHBS buffer, fluorescence of cells was measured on a BD FACS Lyric (Becton-Dickinson) through green and red channels, and data were quantified as the FITC/APC signal ratio, using the BD FACSuite™ Software (Becton-Dickinson). Lipid peroxidation is represented with increasing in green fluorescence. Data are the result of the quantification of 3 independent cell preparations.

### 4.10. Transcription Factor Activation Profiling Arrays

Analysis of transcription factors was performed using Combo TF Activation Profiling Plate. Nuclear extracts were prepared from 5 × 10^5^ cells using the NE-PER Nuclear and Cytoplasmic Extraction Kit (Thermo). A total of 10 μg of the resulting nuclear extract was used to form biotin-labeled protein–DNA complexes. Following elution, the denatured probes were hybridized overnight at 42 °C on a precoated plate containing immobilized target oligonucleotides. After washing, biotin-labeled probes were detected using streptavidin-conjugated horseradish peroxidase (streptavidin-HRP). Luminescence was measured using a Tecan Spark multimode microplate reader controlled by SparkControl Software v3.2, with an integration time of 1 s. Signal intensity was recorded as relative light units (RLUs) after 10 min.

## 5. Conclusions

In conclusion, our findings demonstrate that SM4 directly affects the malignant properties of breast cancer cells by significantly enhancing their invasive capacity, thereby promoting the dissemination of cancer cells into the tumor stroma. Furthermore, elevated SM4 levels on breast cancer cells modulates the transcription of the *BOLA2* gene, which is implicated in apoptotic processes, via the integrin β1/STAT5 signaling pathway. Overall, we discovered a new SM4-dependent signaling pathway describing for the first time the involvement of this glycolipid in the regulation of gene expression, leading to transcriptional changes in proteins involved in both the apoptotic process and invasiveness. The dual effects of SM4—enhancing apoptosis susceptibility while promoting invasiveness—may appear paradoxical, as these phenotypes are often considered antagonistic in tumor progression. Our findings suggest that invasive phenotype occurs under resting conditions, whereas apoptosis sensitivity is revealed only under external stress, such as doxorubicin exposure. Thus, these seemingly antagonistic phenotypes can coexist.

A deeper understanding of these mechanisms would be pivotal in discovering novel therapeutic targets in breast cancer, particularly in approaching the balance between cell death and metastatic potential.

## Data Availability

The data that support the findings of this study are available from the corresponding author upon reasonable request.

## Supplementary Materials

The following supporting information can be downloaded at https://www.mdpi.com/article/10.3390/ijms262411873/s1.
