# Supplementary material for: Sulfatide Acts as a Regulatory Molecule Controlling β1 Integrin–STAT5 Signaling and BOLA2-Dependent Apoptotic Pathway in Breast Cancer Cells
Source: Int J Mol Sci. 2025 Dec 9;26(24):11873. doi: 10.3390/ijms262411873 (PMC12733076; doi:10.3390/ijms262411873)
Supplement: Supplementary file 1 [file ijms-26-11873-s001.zip › Supplementary Materials.docx]

**Supplementary Materials for Suchanski et al.**

**Sulfatide acts as a regulatory molecule controlling β1 integrin–STAT5 signaling and BOLA2-dependent apoptotic pathway in breast cancer cells**

**Supplementary Tables**

**Table S1**: List of primers used in this study, related to the experimental procedures

**Table S2**: Identification of differentially expressed genes (DEGs) in a comparison between

MDA231.CST and MDA231.C

**Table S3:** Identification of differentially expressed genes (DEGs) in a comparison between

MDA468.CST and MDA468.C

**Figure legends**

**Figure S1**: Differential Gene Volcano Map when comparing A) MDA231.CST vs MDA231.C and B) MDA468.CST *vs* MDA468.C; the x-axis shows the fold change in gene expression between different samples, and the y-axis shows the statistical significance of the differences. Red dots represent up-regulation genes, and green dots represent down-regulation genes.

**Figure S2:** SM4 can modulate the quantity of integrins through post-transcriptional regulation. Western blot analysis was performed to assess the expression of integrin subunits in MDA231.CST in comparison to MDA231.C cells, using the Integrin Antibody Sampler Kit (Cell Signaling Technology). Glyceraldehyde 3-phosphate dehydrogenase (GAPDH) served as a loading control.

**Figure S3:** The activity of the TFs was assessed based on each factor's ability to bind specific DNA sequences in cells with high or low sulfatide levels, using the Combo TF Activation Profiling Plate Array-192 (Signosis).

**Figure S4:** Nuclear and cytoplasmic fractions from MDA-MB-231 cells were prepared using the NE-PER Nuclear and Cytoplasmic Extraction Kit (Thermo Scientific, Cat. No. 78833). The purity and efficiency of the subcellular fractionation were verified by Western blot analysis. Histone H3 served as a nuclear marker, while GAPDH was used as a cytoplasmic marker.
