# Supplementary material for: Sulfatide Acts as a Regulatory Molecule Controlling β1 Integrin–STAT5 Signaling and BOLA2-Dependent Apoptotic Pathway in Breast Cancer Cells
Source: Int J Mol Sci. 2025 Dec 9;26(24):11873. doi: 10.3390/ijms262411873 (PMC12733076; doi:10.3390/ijms262411873)
Supplement: Supplementary file 1 [file ijms-26-11873-s001.zip › Table S1.docx]

**Electrophoretic mobility shift assay (EMSA)**

| **STAT5 response element (RE)** | **5’ biotin-labeled primer** | **primer sequence 5’-3’** |
| --- | --- | --- |
| prBOLA2-200bp or prBOLA2/ΔSTAT5-200bp | forSTAT5RE | [Btn]AGTGCAGCGGAGTACTGATG |
|  | revSTAT5RE | [Btn]AGAGTCCAGACGACTTCGGAT |

**Promotor insertion into pGL3-Basic luciferase vector**

| **primer** | **primer sequence 5’-3’** |
| --- | --- |
| forMluI-prBOLA2 | CA**ACGCGT**GTGTTAAGGGAAAGGGGGTG |
| revXhoI-prBOLA2 | GA**CTCGAG**GGAACTCAGGGAAGGAGCTA |

The highlighted region corresponds to the DNA sequence specifically recognized by the restriction enzyme indicated in the primer’s name.

**Real-time-PCR assay**

| **gene** | **primer** | **primer sequence 5’-3’** |
| --- | --- | --- |
| BOLA2 | BOLA2-F | GAATACCTCCGCGAGAAGC |
|  | BOLA2-R | GTTCAAAGGCATGGATGTGC |
| CST | CST-F | GCTGGTGTACTCCTATGCCG |
|  | CST-R | CAAGAACACGATGTTGCGCC |
| GAPDH | GAPDH-F | GGAAGGTGAAGGTCGGAGTC |
|  | GAPDH-R | TGAAGGGGTCATTGATGGCA |

**Expressing vector construction**

| **gen** | **primer** | **primer sequence 5’-3’** |
| --- | --- | --- |
| BOLA2 | forXhoI-BOLA2 | CGC**TCGAG**ATGGAACTCAGCGCCGAAT |
|  | revBamHI-BOLA2 | AT**GGATCC**TCATTTCTGTCGCTCACGTG |
| CST | forEcoRI-CST | GGG**GAATTC**CGCCTCCATGCTGCCACCGCAGAAG |
|  | revBamHI-CST | GGG**GGATCC**CGCCTCCTCACCACCGCAGGAAATCG |
| ITGB1 | forXhoI-ITGB1 | GC**CTCGAG**ATGAATTTACAACCAATTTTCTGGA |
|  | revBamHI-ITGB1 | GC**GGATCC**TCATTTTCCCTCATACTTCGG |

The highlighted region corresponds to the DNA sequence specifically recognized by the restriction enzyme indicated in the primer’s name.
