# Supplementary material for: Sulfatide Acts as a Regulatory Molecule Controlling β1 Integrin–STAT5 Signaling and BOLA2-Dependent Apoptotic Pathway in Breast Cancer Cells
Source: Int J Mol Sci. 2025 Dec 9;26(24):11873. doi: 10.3390/ijms262411873 (PMC12733076; doi:10.3390/ijms262411873)
Supplement: Supplementary file 1 [file ijms-26-11873-s001.zip › Table S2.docx]

| gene_name | MDA231.CST | MDA231.C | log2FoldChange | Gene  chr | Gene  start | Gene  end | Gene  strand | Gene  length | gene_description | |
| --- | --- | --- | --- | --- | --- | --- | --- | --- | --- | --- |
| GAL3ST1 | 177923,09 | 0,00 | 20,48 | 22 | 30554635 | 30574587 | - | 3017 | galactose-3-O-sulfotransferase 1 [Source:HGNC Symbol;Acc:HGNC:24240] | |
| BOLA2B | 22,61 | 497,14 | -4,45 | 16 | 30192934 | 30194306 | - | 1365 | bolA family member 2B [Source:HGNC Symbol;Acc:HGNC:32479] |  |
| XAGE1B | 124,28 | 1050,87 | -3,08 | X | 52512077 | 52520803 | - | 1414 | X antigen family member 1B [Source:HGNC Symbol;Acc:HGNC:25400] |  |
| EIF3CL | 685,32 | 125,07 | 2,45 | 16 | 28379579 | 28403879 | - | 3091 | eukaryotic translation initiation factor 3 subunit C like [Source:HGNC Symbol;Acc:HGNC:26347] |  |
| F8A2 | 2,53 | 41,41 | -3,98 | X | 155382115 | 155383230 | + | 1116 | coagulation factor VIII associated 2 [Source:HGNC Symbol;Acc:HGNC:31849] |  |
| NUTM2B | 2,53 | 35,84 | -3,77 | 10 | 79703227 | 79714681 | + | 5080 | NUT family member 2B [Source:HGNC Symbol;Acc:HGNC:23445] |  |
| ZBTB45P2 | 0,00 | 21,49 | -7,47 | 2 | 110383112 | 110384642 | + | 1531 | zinc finger and BTB domain containing 45 pseudogene 2 [Source:HGNC Symbol;Acc:HGNC:49228] |  |
| RN7SL5P | 1,28 | 27,87 | -4,35 | 9 | 9442060 | 9442380 | + | 321 | RNA, 7SL, cytoplasmic 5, pseudogene [Source:HGNC Symbol;Acc:HGNC:10040] |  |
| PKD1P1 | 11,32 | 64,52 | -2,50 | 16 | 16310341 | 16334190 | + | 6805 | polycystin 1, transient receptor potential channel interacting pseudogene 1 [Source:HGNC Symbol;Acc:HGNC:30065] |  |
| DCN | 0,00 | 18,31 | -7,24 | 12 | 91140484 | 91183123 | - | 12847 | decorin [Source:HGNC Symbol;Acc:HGNC:2705] |  |
| NPIPA7 | 43,95 | 6,36 | 2,76 | 16 | 16379055 | 16393954 | + | 1401 | nuclear pore complex interacting protein family member A7 [Source:HGNC Symbol;Acc:HGNC:41982] |  |
| RPL29P11 | 116,75 | 28,66 | 2,02 | 3 | 37016523 | 37017014 | - | 492 | ribosomal protein L29 pseudogene 11 [Source:HGNC Symbol;Acc:HGNC:36905] |  |
| HERC2P3 | 28,89 | 109,93 | -1,92 | 15 | 20379495 | 20506180 | - | 9413 | hect domain and RLD 2 pseudogene 3 [Source:HGNC Symbol;Acc:HGNC:4871] |  |
| IKBKGP1 | 107,96 | 31,06 | 1,79 | X | 154639978 | 154648275 | - | 1073 | inhibitor of nuclear factor kappa B kinase subunit gamma pseudogene 1 [Source:HGNC Symbol;Acc:HGNC:24455] |  |
| LINC00629 | 15,08 | 0,78 | 4,05 | X | 134549973 | 134559923 | + | 909 | long intergenic non-protein coding RNA 629 [Source:HGNC Symbol;Acc:HGNC:44262] |  |
| AL133255,1 | 15,08 | 0,00 | 6,96 | 6 | 27001208 | 27001648 | - | 441 | novel transcript |  |
| AC078851,1 | 15,08 | 0,00 | 6,96 | 2 | 139824896 | 139825877 | - | 298 | novel transcript |  |
| C9orf163 | 42,70 | 8,75 | 2,27 | 9 | 136483495 | 136486067 | + | 2573 | chromosome 9 open reading frame 163 [Source:HGNC Symbol;Acc:HGNC:26718] |  |
| AL118505,1 | 18,85 | 1,58 | 3,47 | 20 | 6065966 | 6067897 | - | 1932 | novel transcript |  |
| IDH1-AS1 | 2,53 | 23,88 | -3,19 | 2 | 208255247 | 208256181 | + | 655 | IDH1 antisense RNA 1 [Source:HGNC Symbol;Acc:HGNC:40292] |  |
| ZFP2 | 28,89 | 4,76 | 2,56 | 5 | 178895894 | 178933212 | + | 2641 | ZFP2 zinc finger protein [Source:HGNC Symbol;Acc:HGNC:26138] |  |
| WDR72 | 0,00 | 12,73 | -6,72 | 15 | 53513741 | 53762878 | - | 8572 | WD repeat domain 72 [Source:HGNC Symbol;Acc:HGNC:26790] |  |
| SERPINB10 | 0,00 | 12,73 | -6,72 | 18 | 63897174 | 63936111 | + | 2700 | serpin family B member 10 [Source:HGNC Symbol;Acc:HGNC:8942] |  |
| AC146944,2 | 13,83 | 0,00 | 6,84 | 5 | 70462244 | 70479215 | + | 1104 | NLR family, apoptosis inhibitory protein (NAIP) pseudogene |  |
| IGKV1OR9-2 | 13,83 | 0,78 | 3,92 | 9 | 40641841 | 40642117 | - | 277 | immunoglobulin kappa variable 1/OR9-2 (pseudogene) [Source:HGNC Symbol;Acc:HGNC:49466] |  |
| AL359555,2 | 13,83 | 0,00 | 6,84 | 20 | 38270778 | 38274020 | + | 3243 | novel transcript |  |
| FAM198A | 31,40 | 5,56 | 2,47 | 3 | 42979267 | 43060211 | + | 4411 | family with sequence similarity 198 member A [Source:HGNC Symbol;Acc:HGNC:24485] |  |
| AC234783,1 | 17,59 | 1,58 | 3,37 | X | 104062542 | 104063037 | - | 496 | histone H2B pseudogene |  |
| STARD5 | 21,36 | 2,37 | 3,09 | 15 | 81309053 | 81324183 | - | 6339 | StAR related lipid transfer domain containing 5 [Source:HGNC Symbol;Acc:HGNC:18065] |  |
| PSMD10P2 | 1,28 | 19,10 | -3,80 | 3 | 186760693 | 186762050 | + | 1087 | proteasome 26S subunit, non-ATPase, 10 pseudogene 2 [Source:HGNC Symbol;Acc:HGNC:30151] |  |
| AC012615,4 | 23,87 | 3,17 | 2,86 | 19 | 1860250 | 1862019 | + | 607 | novel transcript, antisense to KLF16 |  |
| PCDH7 | 55,25 | 161,72 | -1,55 | 4 | 30720415 | 31146805 | + | 12249 | protocadherin 7 [Source:HGNC Symbol;Acc:HGNC:8659] |  |
| AC004877,2 | 12,57 | 50,18 | -1,99 | 7 | 149851572 | 149852170 | + | 599 | TEC |  |
| AL162741,1 | 16,34 | 1,58 | 3,26 | 1 | 1249777 | 1251334 | - | 1558 | novel transcript |  |
| SNX10 | 92,90 | 30,26 | 1,61 | 7 | 26291895 | 26374329 | + | 3518 | sorting nexin 10 [Source:HGNC Symbol;Acc:HGNC:14974] |  |
| LRRC37A5P | 0,00 | 11,93 | -6,63 | 9 | 111602831 | 111631289 | - | 2324 | leucine rich repeat containing 37 member A5, pseudogene [Source:HGNC Symbol;Acc:HGNC:23369] |  |
| AC011466,1 | 0,00 | 11,14 | -6,53 | 19 | 48204083 | 48213154 | + | 566 | novel transcript, antisense to CARD8 |  |
| AC093627,3 | 0,00 | 11,93 | -6,63 | 7 | 182935 | 194180 | - | 574 | novel transcript |  |
| PRTN3 | 0,00 | 11,14 | -6,53 | 19 | 840960 | 848175 | + | 1146 | proteinase 3 [Source:HGNC Symbol;Acc:HGNC:9495] |  |
| MAATS1 | 0,00 | 11,14 | -6,53 | 3 | 119703022 | 119767102 | + | 7106 | MYCBP associated and testis expressed 1 [Source:HGNC Symbol;Acc:HGNC:24010] |  |
| TBC1D3G | 20,10 | 2,37 | 3,01 | 17 | 36323884 | 36334759 | + | 2065 | TBC1 domain family member 3G [Source:HGNC Symbol;Acc:HGNC:29860] |  |
| EXOC5P1 | 3,79 | 24,68 | -2,67 | 4 | 62816826 | 62818794 | + | 1969 | exocyst complex component 5 pseudogene 1 [Source:HGNC Symbol;Acc:HGNC:43870] |  |
| NKAIN1 | 22,61 | 3,97 | 2,47 | 1 | 31179745 | 31239554 | - | 2987 | sodium/potassium transporting ATPase interacting 1 [Source:HGNC Symbol;Acc:HGNC:25743] |  |
| HLA-V | 18,85 | 2,37 | 2,92 | 6 | 29790954 | 29797811 | + | 5662 | major histocompatibility complex, class I, V (pseudogene) [Source:HGNC Symbol;Acc:HGNC:23482] |  |
| AC106782,1 | 32,65 | 7,15 | 2,17 | 16 | 30204316 | 30209071 | - | 2707 | SAGA complex associated factor 29 pseudogene [Source:NCBI gene;Acc:613038] |  |
| LINC00460 | 6,30 | 31,06 | -2,29 | 13 | 106374477 | 106384315 | + | 4232 | long intergenic non-protein coding RNA 460 [Source:HGNC Symbol;Acc:HGNC:42809] |  |
| AC004232,3 | 11,32 | 0,00 | 6,55 | 16 | 3292879 | 3293403 | + | 525 | TEC |  |
| AC025031,2 | 0,00 | 10,34 | -6,43 | 12 | 46371463 | 46373778 | + | 627 | novel transcript, antisense to SLC38A2 |  |
| AP000688,1 | 0,00 | 10,34 | -6,43 | 21 | 36069642 | 36126640 | - | 1293 | uncharacterized LOC100133286 [Source:NCBI gene;Acc:100133286] |  |
| PAGE5 | 0,00 | 10,34 | -6,43 | X | 55220355 | 55224108 | + | 743 | PAGE family member 5 [Source:HGNC Symbol;Acc:HGNC:29992] |  |
| ACADL | 11,32 | 0,00 | 6,55 | 2 | 210187939 | 210225491 | - | 4383 | acyl-CoA dehydrogenase long chain [Source:HGNC Symbol;Acc:HGNC:88] |  |
| TP53TG3D | 0,00 | 10,34 | -6,43 | 16 | 32252719 | 32255922 | + | 2922 | TP53 target 3D [Source:HGNC Symbol;Acc:HGNC:44657] |  |
| KRT87P | 36,42 | 103,56 | -1,51 | 12 | 52250466 | 52258867 | - | 1778 | keratin 87 pseudogene [Source:HGNC Symbol;Acc:HGNC:30198] |  |
| AP003721,1 | 15,08 | 1,58 | 3,15 | 11 | 60918469 | 60925397 | - | 864 | novel transcript |  |
| AC135977,1 | 21,36 | 3,17 | 2,70 | 11 | 49433480 | 49434438 | - | 959 | solute carrier family 25, member 33 (SLC25A33) pseudogene |  |
| AP003062,1 | 53,99 | 16,71 | 1,68 | 11 | 134950708 | 134951568 | + | 762 | uncharacterized LOC105369586 [Source:NCBI gene;Acc:105369586] |  |
| SERPINB2 | 22,61 | 70,89 | -1,64 | 18 | 63871692 | 63903890 | + | 2748 | serpin family B member 2 [Source:HGNC Symbol;Acc:HGNC:8584] |  |
| INO80B | 5,04 | 27,07 | -2,40 | 2 | 74455023 | 74457960 | + | 1522 | INO80 complex subunit B [Source:HGNC Symbol;Acc:HGNC:13324] |  |
| LDLRAD2 | 8,81 | 37,43 | -2,08 | 1 | 21812265 | 21825221 | + | 4490 | low density lipoprotein receptor class A domain containing 2 [Source:HGNC Symbol;Acc:HGNC:32071] |  |
| Z92544,2 | 36,42 | 9,54 | 1,92 | 16 | 689001 | 692554 | + | 3554 | novel transcript, antisense to FBXL16 |  |
| AP001020,2 | 0,00 | 9,54 | -6,31 | 18 | 738058 | 739662 | - | 1605 | novel transcript, sense intronic toYES1 |  |
| AP001412,1 | 0,00 | 9,54 | -6,31 | 21 | 37267784 | 37268497 | + | 714 | novel transcript, antisense to DSCR3 |  |
| POU3F2 | 0,00 | 9,54 | -6,31 | 6 | 98834592 | 98839470 | + | 4879 | POU class 3 homeobox 2 [Source:HGNC Symbol;Acc:HGNC:9215] |  |
| KCNJ8 | 0,00 | 9,54 | -6,31 | 12 | 21764955 | 21775581 | - | 2484 | potassium voltage-gated channel subfamily J member 8 [Source:HGNC Symbol;Acc:HGNC:6269] |  |
| GVINP2 | 0,00 | 9,54 | -6,31 | 11 | 6748131 | 6750324 | + | 2194 | GTPase, very large interferon inducible pseudogene 2 [Source:HGNC Symbol;Acc:HGNC:38876] |  |
| TRNP1 | 150,64 | 362,50 | -1,27 | 1 | 26993707 | 27000898 | + | 1976 | TMF1-regulated nuclear protein 1 [Source:HGNC Symbol;Acc:HGNC:34348] |  |
| AC087752,4 | 3,79 | 21,49 | -2,48 | 8 | 94884609 | 94885070 | + | 462 | novel transcript, antisense to CCNE2 |  |
| SNORD117 | 22,61 | 4,76 | 2,21 | 6 | 31536374 | 31536449 | - | 76 | small nucleolar RNA, C/D box 117 [Source:HGNC Symbol;Acc:HGNC:32742] |  |
| P4HA3 | 8,81 | 36,63 | -2,05 | 11 | 74235801 | 74311657 | - | 4009 | prolyl 4-hydroxylase subunit alpha 3 [Source:HGNC Symbol;Acc:HGNC:30135] |  |
| PRKG1 | 5,04 | 26,27 | -2,36 | 10 | 50990891 | 52298350 | + | 12831 | protein kinase cGMP-dependent 1 [Source:HGNC Symbol;Acc:HGNC:9414] |  |
| NUDT9P1 | 5,04 | 26,27 | -2,36 | 10 | 91152605 | 91153349 | - | 745 | nudix hydrolase 9 pseudogene 1 [Source:HGNC Symbol;Acc:HGNC:28523] |  |
| DTNA | 33,91 | 93,20 | -1,46 | 18 | 34493290 | 34891844 | + | 12634 | dystrobrevin alpha [Source:HGNC Symbol;Acc:HGNC:3057] |  |
| RF02246 | 16,34 | 2,37 | 2,71 | 2 | 44932320 | 44932406 | + | 87 |  |  |
| AC233992,2 | 1,28 | 15,12 | -3,47 | 8 | 144353228 | 144355609 | - | 1596 | novel transcript |  |
| PRICKLE4 | 1,28 | 15,12 | -3,47 | 6 | 41780762 | 41787372 | + | 2718 | prickle planar cell polarity protein 4 [Source:HGNC Symbol;Acc:HGNC:16805] |  |
| TMEM75 | 1,28 | 15,92 | -3,54 | 8 | 127946559 | 127948723 | - | 2165 | transmembrane protein 75 [Source:HGNC Symbol;Acc:HGNC:32295] |  |
| AC024361,1 | 13,83 | 1,58 | 3,02 | 17 | 82745068 | 82745709 | + | 642 | novel transcript, sense intronic FN3K |  |
| TAF1L | 13,83 | 1,58 | 3,02 | 9 | 32629454 | 32635669 | - | 6216 | TATA-box binding protein associated factor 1 like [Source:HGNC Symbol;Acc:HGNC:18056] |  |
| HLA-DQB2 | 13,83 | 1,58 | 3,02 | 6 | 32756098 | 32763534 | - | 2028 | major histocompatibility complex, class II, DQ beta 2 [Source:HGNC Symbol;Acc:HGNC:4945] |  |
| PIFO | 25,12 | 5,56 | 2,15 | 1 | 111346288 | 111353013 | + | 4261 | primary cilia formation [Source:HGNC Symbol;Acc:HGNC:27009] |  |
| SESN3 | 42,70 | 112,32 | -1,39 | 11 | 95165513 | 95232541 | - | 10573 | sestrin 3 [Source:HGNC Symbol;Acc:HGNC:23060] |  |
| AP001178,3 | 21,36 | 4,76 | 2,13 | 18 | 650229 | 652843 | + | 2106 | novel transcript, antisense to C18orf56 |  |
| TBC1D30 | 17,59 | 53,36 | -1,60 | 12 | 64780516 | 64881032 | + | 9325 | TBC1 domain family member 30 [Source:HGNC Symbol;Acc:HGNC:29164] |  |
| ZNF521 | 2,53 | 17,51 | -2,75 | 18 | 25061926 | 25352190 | - | 6223 | zinc finger protein 521 [Source:HGNC Symbol;Acc:HGNC:24605] |  |
| TMC4 | 23,87 | 5,56 | 2,07 | 19 | 54160108 | 54173250 | - | 3062 | transmembrane channel like 4 [Source:HGNC Symbol;Acc:HGNC:22998] |  |
| AC090409,2 | 23,87 | 5,56 | 2,07 | 18 | 61571342 | 61579456 | - | 610 | novel transcript |  |
| GJC1 | 0,00 | 8,75 | -6,19 | 17 | 44798448 | 44830816 | - | 8660 | gap junction protein gamma 1 [Source:HGNC Symbol;Acc:HGNC:4280] |  |
| MME | 0,00 | 8,75 | -6,19 | 3 | 155024124 | 155183729 | + | 9620 | membrane metalloendopeptidase [Source:HGNC Symbol;Acc:HGNC:7154] |  |
| AC114730,3 | 0,00 | 8,75 | -6,19 | 2 | 241724615 | 241725693 | - | 584 | novel transcript |  |
| CYP2W1 | 0,00 | 8,75 | -6,19 | 7 | 983199 | 989640 | + | 2773 | cytochrome P450 family 2 subfamily W member 1 [Source:HGNC Symbol;Acc:HGNC:20243] |  |
| AP000560,1 | 0,00 | 8,75 | -6,19 | 11 | 74830574 | 74832510 | - | 1937 | TEC |  |
| AC119403,1 | 0,00 | 8,75 | -6,19 | 19 | 2915146 | 2926807 | - | 776 | novel transcript, antisense to ZNF57 |  |
| AC007405,2 | 0,00 | 8,75 | -6,19 | 2 | 170816293 | 170818037 | - | 832 | novel transcript |  |
| LINC00954 | 10,06 | 0,00 | 6,38 | 2 | 19868860 | 19885047 | + | 4808 | long intergenic non-protein coding RNA 954 [Source:HGNC Symbol;Acc:HGNC:48668] |  |
| IGLON5 | 0,00 | 8,75 | -6,19 | 19 | 51311848 | 51330354 | + | 2606 | IgLON family member 5 [Source:HGNC Symbol;Acc:HGNC:34550] |  |
| ADGRL3 | 0,00 | 8,75 | -6,19 | 4 | 61201258 | 62078335 | + | 13991 | adhesion G protein-coupled receptor L3 [Source:HGNC Symbol;Acc:HGNC:20974] |  |
| RPS10P5 | 1,28 | 14,32 | -3,39 | 20 | 839447 | 839977 | - | 531 | ribosomal protein S10 pseudogene 5 [Source:HGNC Symbol;Acc:HGNC:15795] |  |
| C3orf49 | 1,28 | 14,32 | -3,39 | 3 | 63819362 | 63848636 | + | 1581 | chromosome 3 open reading frame 49 [Source:HGNC Symbol;Acc:HGNC:25190] |  |
| DTX2P1 | 1,28 | 14,32 | -3,39 | 7 | 76978617 | 77004308 | + | 1633 | DTX2 pseudogene 1 [Source:HGNC Symbol;Acc:HGNC:42352] |  |
| RGPD5 | 30,14 | 80,45 | -1,41 | 2 | 109792758 | 109857695 | + | 14175 | RANBP2-like and GRIP domain containing 5 [Source:HGNC Symbol;Acc:HGNC:32418] |  |
| FAM71E1 | 26,38 | 6,36 | 2,03 | 19 | 50466785 | 50476753 | - | 1566 | family with sequence similarity 71 member E1 [Source:HGNC Symbol;Acc:HGNC:25107] |  |
| DEPDC7 | 3,79 | 19,10 | -2,31 | 11 | 33015864 | 33033582 | + | 3326 | DEP domain containing 7 [Source:HGNC Symbol;Acc:HGNC:29899] |  |
| AC135048,4 | 3,79 | 19,10 | -2,31 | 16 | 30984630 | 30988270 | - | 3641 | novel transcript |  |
| AL442125,1 | 55,25 | 19,90 | 1,47 | 13 | 113527260 | 113530621 | + | 3362 | novel transcript, sense intronic to TMCO3 |  |
| TBC1D3E | 13,83 | 44,60 | -1,68 | 17 | 38127951 | 38138862 | + | 2076 | TBC1 domain family member 3E [Source:HGNC Symbol;Acc:HGNC:27071] |  |
| SLITRK5 | 13,83 | 44,60 | -1,68 | 13 | 87672615 | 87696272 | + | 21103 | SLIT and NTRK like family member 5 [Source:HGNC Symbol;Acc:HGNC:20295] |  |
| ARHGAP30 | 20,10 | 57,35 | -1,51 | 1 | 161046946 | 161069970 | - | 5064 | Rho GTPase activating protein 30 [Source:HGNC Symbol;Acc:HGNC:27414] |  |
| HRCT1 | 7,55 | 28,66 | -1,91 | 9 | 35906192 | 35907141 | + | 950 | histidine rich carboxyl terminus 1 [Source:HGNC Symbol;Acc:HGNC:33872] |  |
| THSD1 | 7,55 | 28,66 | -1,91 | 13 | 52377167 | 52416373 | - | 3705 | thrombospondin type 1 domain containing 1 [Source:HGNC Symbol;Acc:HGNC:17754] |  |
| AC087071,2 | 30,14 | 8,75 | 1,77 | 7 | 130173718 | 130205361 | + | 575 | novel transcript |  |
| SEMA3A | 128,04 | 285,21 | -1,15 | 7 | 83955777 | 84492724 | - | 9371 | semaphorin 3A [Source:HGNC Symbol;Acc:HGNC:10723] |  |
| NR1I3 | 2,53 | 16,71 | -2,68 | 1 | 161229666 | 161238302 | - | 3305 | nuclear receptor subfamily 1 group I member 3 [Source:HGNC Symbol;Acc:HGNC:7969] |  |
| RSAD2 | 32,65 | 84,44 | -1,37 | 2 | 6865806 | 6898239 | + | 4834 | radical S-adenosyl methionine domain containing 2 [Source:HGNC Symbol;Acc:HGNC:30908] |  |
| LTB | 27,63 | 71,69 | -1,37 | 6 | 31580525 | 31582522 | - | 1535 | lymphotoxin beta [Source:HGNC Symbol;Acc:HGNC:6711] |  |
| AP003469,4 | 8,81 | 32,65 | -1,88 | 8 | 101166805 | 101169629 | - | 2825 | novel transcript |  |
| QRFP | 33,91 | 10,34 | 1,70 | 9 | 130892702 | 130896812 | - | 1658 | pyroglutamylated RFamide peptide [Source:HGNC Symbol;Acc:HGNC:29982] |  |
| SLC4A8 | 32,65 | 82,84 | -1,34 | 12 | 51391317 | 51515763 | + | 17980 | solute carrier family 4 member 8 [Source:HGNC Symbol;Acc:HGNC:11034] |  |
| TDRKH-AS1 | 26,38 | 7,15 | 1,86 | 1 | 151790804 | 151794402 | + | 1141 | TDRKH antisense RNA 1 [Source:HGNC Symbol;Acc:HGNC:40578] |  |
| AL645929,2 | 26,38 | 7,15 | 1,86 | 6 | 29849550 | 29885615 | - | 3405 | MHC class I polypeptide-related sequence F pseudogene |  |
| CHRNA3 | 1,28 | 13,53 | -3,31 | 15 | 78593052 | 78621295 | - | 4765 | cholinergic receptor nicotinic alpha 3 subunit [Source:HGNC Symbol;Acc:HGNC:1957] |  |
| LINC00982 | 15,08 | 2,37 | 2,60 | 1 | 3059615 | 3068437 | - | 5600 | long intergenic non-protein coding RNA 982 [Source:HGNC Symbol;Acc:HGNC:48664] |  |
| TINCR | 0,00 | 7,95 | -6,05 | 19 | 5558167 | 5578349 | - | 4429 | TINCR ubiquitin domain containing [Source:HGNC Symbol;Acc:HGNC:14607] |  |
| AC110619,1 | 0,00 | 7,95 | -6,05 | 2 | 240449315 | 240456714 | - | 3235 | uncharacterized LOC100130449 [Source:NCBI gene;Acc:100130449] |  |
| RASL10A | 0,00 | 7,95 | -6,05 | 22 | 29312933 | 29319679 | - | 2240 | RAS like family 10 member A [Source:HGNC Symbol;Acc:HGNC:16954] |  |
| AP001029,1 | 0,00 | 7,95 | -6,05 | 18 | 12432897 | 12437635 | + | 168 | novel transcript |  |
| AL606760,1 | 8,81 | 0,00 | 6,19 | 1 | 53238610 | 53242783 | + | 1181 | uncharacterized LOC100507564 [Source:NCBI gene;Acc:100507564] |  |
| ERICH6-AS1 | 0,00 | 7,95 | -6,05 | 3 | 150703564 | 150720146 | + | 827 | ERICH6 antisense RNA 1 [Source:HGNC Symbol;Acc:HGNC:41205] |  |
| AL122023,1 | 0,00 | 7,95 | -6,05 | 14 | 93334528 | 93335057 | + | 530 | novel transcript, sense intronic UNC79 |  |
| MIR6859-3 | 0,00 | 7,95 | -6,05 | 15 | 101973524 | 101973591 | + | 68 | microRNA 6859-3 [Source:HGNC Symbol;Acc:HGNC:50248] |  |
| AP001330,5 | 0,00 | 7,95 | -6,05 | 8 | 101208148 | 101208558 | + | 411 | novel transcript |  |
| AL359258,3 | 0,00 | 7,95 | -6,05 | 1 | 108200413 | 108202743 | + | 2331 | TEC |  |
| AC243654,3 | 8,81 | 0,00 | 6,19 | 17 | 37386886 | 37387926 | + | 735 | novel transcript, sense intronic to C17orf78 |  |
| RNA5SP187 | 8,81 | 0,00 | 6,19 | 5 | 88274258 | 88274376 | - | 119 | RNA, 5S ribosomal pseudogene 187 [Source:HGNC Symbol;Acc:HGNC:43087] |  |
| DEPDC1-AS1 | 0,00 | 7,95 | -6,05 | 1 | 68496676 | 68538627 | + | 1247 | DEPDC1 antisense RNA 1 [Source:HGNC Symbol;Acc:HGNC:50592] |  |
| KIRREL1-IT1 | 0,00 | 7,95 | -6,05 | 1 | 158025550 | 158031166 | + | 587 | KIRREL1 intronic transcript 1 [Source:HGNC Symbol;Acc:HGNC:41412] |  |
| AC100788,2 | 0,00 | 7,95 | -6,05 | 17 | 78897264 | 78900152 | + | 2889 | TEC |  |
| Z82217,1 | 8,81 | 0,00 | 6,19 | 22 | 35992321 | 36000469 | + | 8149 | novel transcript |  |
| AC010273,2 | 8,81 | 0,00 | 6,19 | 5 | 69038518 | 69043821 | - | 438 | novel transcript |  |
| AC122134,1 | 0,00 | 7,95 | -6,05 | 2 | 234222838 | 234224514 | + | 1677 | novel transcript |  |
| OPA1-AS1 | 0,00 | 7,95 | -6,05 | 3 | 193618609 | 193627337 | - | 497 | OPA1 antisense RNA 1 [Source:HGNC Symbol;Acc:HGNC:40421] |  |
| PARP15 | 0,00 | 7,95 | -6,05 | 3 | 122577602 | 122639047 | + | 5507 | poly(ADP-ribose) polymerase family member 15 [Source:HGNC Symbol;Acc:HGNC:26876] |  |
| AC007262,2 | 8,81 | 0,00 | 6,19 | 14 | 81012099 | 81170414 | - | 8011 | uncharacterized LOC101928462 [Source:NCBI gene;Acc:101928462] |  |
| C16orf96 | 8,81 | 0,00 | 6,19 | 16 | 4556490 | 4600714 | + | 3823 | chromosome 16 open reading frame 96 [Source:HGNC Symbol;Acc:HGNC:40031] |  |
| SERF1B | 55,25 | 127,46 | -1,21 | 5 | 70025247 | 70043113 | + | 3051 | small EDRK-rich factor 1B [Source:HGNC Symbol;Acc:HGNC:10756] |  |
| TCEA1P2 | 230,96 | 106,74 | 1,11 | 3 | 37275693 | 37276598 | + | 906 | transcription elongation factor A1 pseudogene 2 [Source:HGNC Symbol;Acc:HGNC:29891] |  |
| HOOK1 | 15,08 | 44,60 | -1,56 | 1 | 59814786 | 59876378 | + | 6758 | hook microtubule tethering protein 1 [Source:HGNC Symbol;Acc:HGNC:19884] |  |
| C6orf223 | 15,08 | 44,60 | -1,56 | 6 | 44000580 | 44005958 | + | 3917 | chromosome 6 open reading frame 223 [Source:HGNC Symbol;Acc:HGNC:28692] |  |
| TNXA | 11,32 | 1,58 | 2,73 | 6 | 32008614 | 32012472 | - | 2038 | tenascin XA (pseudogene) [Source:HGNC Symbol;Acc:HGNC:11975] |  |
| AL133329,1 | 11,32 | 1,58 | 2,73 | X | 42047883 | 42054836 | - | 478 | novel transcript |  |
| GPR89B | 277,40 | 129,85 | 1,09 | 1 | 147928393 | 147993521 | + | 5143 | G protein-coupled receptor 89B [Source:HGNC Symbol;Acc:HGNC:13840] |  |
| IL11 | 380,32 | 786,36 | -1,05 | 19 | 55364389 | 55370463 | - | 2671 | interleukin 11 [Source:HGNC Symbol;Acc:HGNC:5966] |  |
| UPF3AP2 | 2,53 | 15,12 | -2,54 | 17 | 20375369 | 20376840 | - | 1472 | UPF3A pseudogene 2 [Source:HGNC Symbol;Acc:HGNC:30567] |  |
| AC008592,3 | 2,53 | 15,12 | -2,54 | 5 | 95834424 | 95835046 | - | 418 | gamma-glutamyl cyclotransferase (GGCT) pseudogene |  |
| AC106820,4 | 2,53 | 15,12 | -2,54 | 16 | 2476558 | 2482173 | + | 5616 | novel transcript, intronic to TBC1D24 |  |
| SAMD14 | 16,34 | 3,17 | 2,31 | 17 | 50110040 | 50129882 | - | 7016 | sterile alpha motif domain containing 14 [Source:HGNC Symbol;Acc:HGNC:27312] |  |
| AC010201,2 | 16,34 | 3,17 | 2,31 | 12 | 89367807 | 89369301 | + | 1495 | novel transcript |  |
| AC110792,3 | 2,53 | 15,12 | -2,54 | 4 | 53997415 | 53997712 | - | 298 | novel transcript |  |
| AL158206,1 | 12,57 | 37,43 | -1,57 | 9 | 19453209 | 19455173 | + | 1965 | novel transcript, overlapping ACER2 |  |
| AC026471,1 | 15,08 | 43,80 | -1,53 | 16 | 31456711 | 31459736 | - | 3026 | novel transcript, antisense to ARMC5 |  |
| FAM21FP | 46,46 | 17,51 | 1,40 | 10 | 45706431 | 45727231 | - | 2087 | family with sequence similarity 21 member F, pseudogene [Source:HGNC Symbol;Acc:HGNC:45011] |  |
| HS6ST3 | 17,59 | 47,79 | -1,44 | 13 | 96090839 | 96839562 | + | 7806 | heparan sulfate 6-O-sulfotransferase 3 [Source:HGNC Symbol;Acc:HGNC:19134] |  |
| AMY2B | 18,85 | 3,97 | 2,21 | 1 | 103553815 | 103579534 | + | 7491 | amylase, alpha 2B (pancreatic) [Source:HGNC Symbol;Acc:HGNC:478] |  |
| AC053513,1 | 3,79 | 17,51 | -2,18 | 12 | 22460519 | 22463914 | - | 738 | novel transcript |  |
| Z69733,1 | 18,85 | 4,76 | 1,95 | X | 103497523 | 103500317 | + | 632 | novel transcript |  |
| AC093110,1 | 8,81 | 28,66 | -1,69 | 2 | 54661011 | 54680045 | - | 1236 | novel transcript, antisense to SPTBN1 |  |
| LRRN1 | 30,14 | 9,54 | 1,64 | 3 | 3799437 | 3847703 | + | 4264 | leucine rich repeat neuronal 1 [Source:HGNC Symbol;Acc:HGNC:20980] |  |
| IL34 | 62,78 | 25,48 | 1,30 | 16 | 70579895 | 70660682 | + | 2233 | interleukin 34 [Source:HGNC Symbol;Acc:HGNC:28529] |  |
| TBC1D8-AS1 | 1,28 | 12,73 | -3,22 | 2 | 101151660 | 101155412 | + | 2478 | TBC1 domain family member 8 antisense RNA 1 [Source:HGNC Symbol;Acc:HGNC:52782] |  |
| EIF1P6 | 1,28 | 12,73 | -3,22 | 19 | 4347780 | 4349061 | - | 334 | eukaryotic translation initiation factor 1 pseudogene 6 [Source:HGNC Symbol;Acc:HGNC:49619] |  |
| DLEU7 | 1,28 | 12,73 | -3,22 | 13 | 50711008 | 50843939 | - | 3656 | deleted in lymphocytic leukemia, 7 [Source:HGNC Symbol;Acc:HGNC:17567] |  |
| AC139713,2 | 1,28 | 12,73 | -3,22 | 4 | 143559457 | 144188367 | + | 12703 | uncharacterized LOC101927636 [Source:NCBI gene;Acc:101927636] |  |
| AC104453,1 | 51,48 | 20,70 | 1,31 | 1 | 98052077 | 98054592 | - | 1784 | novel transcript |  |
| TAS2R14 | 20,10 | 52,57 | -1,38 | 12 | 10937406 | 11171573 | - | 2350 | taste 2 receptor member 14 [Source:HGNC Symbol;Acc:HGNC:14920] |  |
| ZNF154 | 22,61 | 6,36 | 1,81 | 19 | 57697367 | 57709194 | - | 5713 | zinc finger protein 154 [Source:HGNC Symbol;Acc:HGNC:12939] |  |
| MEIS3P2 | 6,30 | 23,09 | -1,86 | 17 | 20589293 | 20590367 | + | 1075 | Meis homeobox 3 pseudogene 2 [Source:HGNC Symbol;Acc:HGNC:17638] |  |
| ULBP3 | 41,44 | 93,20 | -1,17 | 6 | 150063150 | 150069095 | - | 988 | UL16 binding protein 3 [Source:HGNC Symbol;Acc:HGNC:14895] |  |
| TM4SF19-AS1 | 26,38 | 8,75 | 1,58 | 3 | 196318330 | 196325570 | + | 2136 | TM4SF19 antisense RNA 1 [Source:HGNC Symbol;Acc:HGNC:41085] |  |
| SMG1P5 | 36,42 | 83,64 | -1,20 | 16 | 30267553 | 30335374 | - | 4791 | SMG1 pseudogene 5 [Source:HGNC Symbol;Acc:HGNC:49862] |  |
| PRMT5-AS1 | 2,53 | 14,32 | -2,46 | 14 | 22918947 | 22926900 | + | 3194 | PRMT5 antisense RNA 1 [Source:HGNC Symbol;Acc:HGNC:40533] |  |
| AL133338,1 | 8,81 | 27,87 | -1,65 | 6 | 100881471 | 100882987 | + | 1517 | novel transcript, antisense to ASCC3 |  |
| APOBR | 8,81 | 27,07 | -1,61 | 16 | 28494649 | 28498970 | + | 3792 | apolipoprotein B receptor [Source:HGNC Symbol;Acc:HGNC:24087] |  |
| NPY1R | 10,06 | 1,58 | 2,57 | 4 | 163323961 | 163344832 | - | 4180 | neuropeptide Y receptor Y1 [Source:HGNC Symbol;Acc:HGNC:7956] |  |
| AC073063,1 | 10,06 | 1,58 | 2,57 | 7 | 99442890 | 99443496 | - | 607 | cytokine induced protein 29 kDa (CIP29) pseudogene |  |
| OPRD1 | 10,06 | 1,58 | 2,57 | 1 | 28812142 | 28871267 | + | 9345 | opioid receptor delta 1 [Source:HGNC Symbol;Acc:HGNC:8153] |  |
| GFOD1-AS1 | 10,06 | 1,58 | 2,57 | 6 | 13486294 | 13486852 | + | 332 | GFOD1 antisense RNA 1 [Source:HGNC Symbol;Acc:HGNC:40956] |  |
| AC087491,1 | 10,06 | 1,58 | 2,57 | 17 | 39619613 | 39622513 | + | 616 | novel transcript |  |
| ABCA6 | 10,06 | 1,58 | 2,57 | 17 | 69078702 | 69141888 | - | 14151 | ATP binding cassette subfamily A member 6 [Source:HGNC Symbol;Acc:HGNC:36] |  |
| NPR3 | 12,57 | 35,84 | -1,51 | 5 | 32689070 | 32791724 | + | 9866 | natriuretic peptide receptor 3 [Source:HGNC Symbol;Acc:HGNC:7945] |  |
| AL590705,1 | 48,97 | 19,10 | 1,35 | 9 | 97200475 | 97238700 | - | 806 | novel transcript |  |
| AC005034,5 | 17,59 | 45,40 | -1,36 | 2 | 75669989 | 75670454 | + | 466 | novel transcript, sense intronic to MRPL19 |  |
| AK4P3 | 84,11 | 175,26 | -1,06 | 12 | 31615771 | 31616439 | - | 669 | adenylate kinase 4 pseudogene 3 [Source:HGNC Symbol;Acc:HGNC:21596] |  |
| NEFH | 0,00 | 6,36 | -5,74 | 22 | 29480230 | 29491390 | + | 3783 | neurofilament heavy [Source:HGNC Symbol;Acc:HGNC:7737] |  |
| LGR6 | 0,00 | 6,36 | -5,74 | 1 | 202193901 | 202319781 | + | 4583 | leucine rich repeat containing G protein-coupled receptor 6 [Source:HGNC Symbol;Acc:HGNC:19719] |  |
| FAM83E | 0,00 | 6,36 | -5,74 | 19 | 48600810 | 48614854 | - | 2529 | family with sequence similarity 83 member E [Source:HGNC Symbol;Acc:HGNC:25972] |  |
| HEPHL1 | 0,00 | 6,36 | -5,74 | 11 | 94021361 | 94113751 | + | 5345 | hephaestin like 1 [Source:HGNC Symbol;Acc:HGNC:30477] |  |
| BMS1P7 | 0,00 | 6,36 | -5,74 | 10 | 48050282 | 48060016 | - | 1052 | BMS1, ribosome biogenesis factor pseudogene 7 [Source:HGNC Symbol;Acc:HGNC:23655] |  |
| SYN1 | 0,00 | 7,15 | -5,90 | X | 47571898 | 47619943 | - | 3822 | synapsin I [Source:HGNC Symbol;Acc:HGNC:11494] |  |
| AC093274,1 | 0,00 | 7,15 | -5,90 | 5 | 21323873 | 21341375 | - | 5454 | novel transcript |  |
| LINC00882 | 0,00 | 6,36 | -5,74 | 3 | 106836811 | 107240641 | - | 3882 | long intergenic non-protein coding RNA 882 [Source:HGNC Symbol;Acc:HGNC:48568] |  |
| AC004982,1 | 7,55 | 0,00 | 5,97 | 7 | 7550104 | 7552440 | - | 1655 | novel transcript |  |
| AC106738,1 | 7,55 | 0,00 | 5,97 | 16 | 54934913 | 54954665 | + | 359 | novel transcript |  |
| AC009486,1 | 0,00 | 7,15 | -5,90 | 2 | 12715415 | 12716227 | + | 813 | novel transcript |  |
| MUC20P1 | 0,00 | 6,36 | -5,74 | 3 | 195614947 | 195620233 | + | 1680 | mucin 20, cell surface associated pseudogene 1 [Source:HGNC Symbol;Acc:HGNC:51921] |  |
| SLX1B | 7,55 | 0,00 | 5,97 | 16 | 29454501 | 29458219 | + | 2462 | SLX1 homolog B, structure-specific endonuclease subunit [Source:HGNC Symbol;Acc:HGNC:28748] |  |
| AC004466,1 | 0,00 | 6,36 | -5,74 | 12 | 47784923 | 47786002 | + | 1080 | novel transcript, antisense to HDAC7 |  |
| SERPINE3 | 0,00 | 6,36 | -5,74 | 13 | 51335773 | 51364735 | + | 2608 | serpin family E member 3 [Source:HGNC Symbol;Acc:HGNC:24774] |  |
| AC009220,3 | 0,00 | 6,36 | -5,74 | 7 | 139198557 | 139199142 | - | 586 | FCF1 small subunit (SSU) processome component homolog (S, cerevisiae), pseudogene |  |
| PPATP1 | 7,55 | 0,00 | 5,97 | 3 | 87051192 | 87052738 | - | 1547 | phosphoribosyl pyrophosphate amidotransferase pseudogene 1 [Source:HGNC Symbol;Acc:HGNC:9239] |  |
| AC004771,1 | 7,55 | 0,00 | 5,97 | 17 | 5019214 | 5020093 | - | 583 | uncharacterized LOC102724009 [Source:NCBI gene;Acc:102724009] |  |
| FO704657,1 | 0,00 | 7,15 | -5,90 | 1 | 1659325 | 1662602 | + | 400 | novel transcript, antisense to SLC35E2B |  |
| AC069234,4 | 0,00 | 6,36 | -5,74 | 12 | 120709112 | 120709523 | + | 412 | novel transcript |  |
| OTUD7A | 0,00 | 6,36 | -5,74 | 15 | 31475398 | 31870789 | - | 14331 | OTU deubiquitinase 7A [Source:HGNC Symbol;Acc:HGNC:20718] |  |
| AL049780,2 | 0,00 | 6,36 | -5,74 | 14 | 75127153 | 75136930 | + | 367 | novel transcript, antisense to NEK9 and TMED10 |  |
| PYY2 | 0,00 | 6,36 | -5,74 | 17 | 28226563 | 28228065 | + | 1076 | peptide YY 2 (pseudogene) [Source:HGNC Symbol;Acc:HGNC:9749] |  |
| ARHGEF26-AS1 | 0,00 | 6,36 | -5,74 | 3 | 154024401 | 154121332 | - | 6580 | ARHGEF26 antisense RNA 1 [Source:HGNC Symbol;Acc:HGNC:41048] |  |
| AC004263,1 | 0,00 | 7,15 | -5,90 | 12 | 120224744 | 120225421 | + | 678 | novel transcript, antisense to PXN |  |
| AC087301,1 | 0,00 | 7,15 | -5,90 | 17 | 73243093 | 73244706 | + | 321 | novel transcript, sense intronic to C17orf80 |  |
| TTLL9 | 7,55 | 0,00 | 5,97 | 20 | 31870702 | 31944963 | + | 6175 | tubulin tyrosine ligase like 9 [Source:HGNC Symbol;Acc:HGNC:16118] |  |
| SLC6A7 | 0,00 | 7,15 | -5,90 | 5 | 150189957 | 150222788 | + | 4391 | solute carrier family 6 member 7 [Source:HGNC Symbol;Acc:HGNC:11054] |  |
| AC003965,1 | 0,00 | 6,36 | -5,74 | 16 | 2866348 | 2867618 | - | 667 | novel transcript |  |
| KRT79 | 0,00 | 6,36 | -5,74 | 12 | 52821410 | 52834295 | - | 2542 | keratin 79 [Source:HGNC Symbol;Acc:HGNC:28930] |  |
| Z93930,3 | 0,00 | 6,36 | -5,74 | 22 | 28814914 | 28815662 | + | 749 | novel transcript |  |
| C1orf100 | 7,55 | 0,00 | 5,97 | 1 | 244352635 | 244389663 | + | 1151 | chromosome 1 open reading frame 100 [Source:HGNC Symbol;Acc:HGNC:30435] |  |
| AC104651,1 | 0,00 | 6,36 | -5,74 | 2 | 111940302 | 111941036 | - | 735 | chromosome 14 open reading frame 166 (C14orf166) pseudogene |  |
| LINC01220 | 7,55 | 0,00 | 5,97 | 14 | 75294404 | 75296638 | + | 718 | long intergenic non-protein coding RNA 1220 [Source:HGNC Symbol;Acc:HGNC:49664] |  |
| AC013472,3 | 0,00 | 6,36 | -5,74 | 2 | 27053618 | 27054276 | - | 659 | novel transcript, antisense to AGBL5 |  |
| AL359715,4 | 0,00 | 6,36 | -5,74 | 6 | 80440730 | 80441172 | + | 443 | TEC |  |
| IGSF10 | 0,00 | 6,36 | -5,74 | 3 | 151425384 | 151458709 | - | 11727 | immunoglobulin superfamily member 10 [Source:HGNC Symbol;Acc:HGNC:26384] |  |
| HOXB8 | 0,00 | 6,36 | -5,74 | 17 | 48611377 | 48614939 | - | 2051 | homeobox B8 [Source:HGNC Symbol;Acc:HGNC:5119] |  |
| AC006116,5 | 0,00 | 6,36 | -5,74 | 19 | 56311928 | 56312486 | - | 559 | novel transcript, sense intronic to ZSCAN5A |  |
| RPL23AP25 | 7,55 | 0,00 | 5,97 | 1 | 248936581 | 248937043 | + | 463 | ribosomal protein L23a pseudogene 25 [Source:HGNC Symbol;Acc:HGNC:35672] |  |
| KLF7-IT1 | 0,00 | 7,15 | -5,90 | 2 | 207120884 | 207122044 | - | 655 | KLF7 intronic transcript 1 [Source:HGNC Symbol;Acc:HGNC:41355] |  |
| BMS1P10 | 0,00 | 6,36 | -5,74 | 9 | 63299453 | 63385117 | - | 1596 | BMS1, ribosome biogenesis factor pseudogene 10 [Source:HGNC Symbol;Acc:HGNC:49154] |  |
| AC007998,3 | 0,00 | 6,36 | -5,74 | 18 | 35443869 | 35467088 | - | 916 | novel transcript |  |
| AC018761,3 | 0,00 | 6,36 | -5,74 | 19 | 12784539 | 12785101 | - | 388 | novel transcript |  |
| CHGA | 7,55 | 0,00 | 5,97 | 14 | 92923080 | 92935293 | + | 3102 | chromogranin A [Source:HGNC Symbol;Acc:HGNC:1929] |  |
| RF00012 | 7,55 | 0,00 | 5,97 | 4 | 158700691 | 158700909 | + | 219 |  |  |
| RF00019 | 7,55 | 0,00 | 5,97 | 17 | 82417226 | 82417321 | + | 96 |  |  |
| MIR133A1HG | 0,00 | 6,36 | -5,74 | 18 | 21825487 | 21831410 | - | 4376 | MIR133A1 host gene [Source:HGNC Symbol;Acc:HGNC:49594] |  |
| AC079766,1 | 0,00 | 6,36 | -5,74 | 4 | 182881777 | 182882203 | - | 427 | novel transcript |  |
| FAM95B1 | 0,00 | 6,36 | -5,74 | 9 | 40321299 | 40329221 | + | 7923 | family with sequence similarity 95 member B1 [Source:HGNC Symbol;Acc:HGNC:32318] |  |
| HSFX4 | 7,55 | 0,00 | 5,97 | X | 149929645 | 149931287 | + | 1184 | heat shock transcription factor family, X-linked member 4 [Source:HGNC Symbol;Acc:HGNC:52398] |  |
| SNORD37 | 7,55 | 0,00 | 5,97 | 19 | 3982507 | 3982572 | - | 66 | small nucleolar RNA, C/D box 37 [Source:HGNC Symbol;Acc:HGNC:10166] |  |
| CACNA1S | 0,00 | 6,36 | -5,74 | 1 | 201039512 | 201112566 | - | 6168 | calcium voltage-gated channel subunit alpha1 S [Source:HGNC Symbol;Acc:HGNC:1397] |  |
| UBXN7-AS1 | 7,55 | 0,00 | 5,97 | 3 | 196431385 | 196432530 | + | 386 | UBXN7 antisense RNA 1 [Source:HGNC Symbol;Acc:HGNC:41227] |  |
| AC128709,2 | 0,00 | 6,36 | -5,74 | 3 | 197445061 | 197458323 | - | 558 | novel transcript |  |
| RN7SL748P | 0,00 | 6,36 | -5,74 | 6 | 36522191 | 36522501 | + | 311 | RNA, 7SL, cytoplasmic 748, pseudogene [Source:HGNC Symbol;Acc:HGNC:46764] |  |
| PTPRQ | 0,00 | 6,36 | -5,74 | 12 | 80402178 | 80680271 | + | 12477 | protein tyrosine phosphatase, receptor type Q [Source:HGNC Symbol;Acc:HGNC:9679] |  |
| AC010182,1 | 0,00 | 6,36 | -5,74 | 12 | 106170004 | 106172437 | + | 2434 | thyroid hormone receptor interactor 11 (TRIP11) pseudogene |  |
| AL035658,1 | 7,55 | 0,00 | 5,97 | 22 | 41169190 | 41183144 | - | 357 | novel transcript, antisense to EP300 |  |
| AC113385,3 | 0,00 | 6,36 | -5,74 | 5 | 100375700 | 100381398 | - | 747 | glucuronidase, beta (GUSB) pseudogene |  |
| GRIA1 | 0,00 | 6,36 | -5,74 | 5 | 153489615 | 153813869 | + | 7636 | glutamate ionotropic receptor AMPA type subunit 1 [Source:HGNC Symbol;Acc:HGNC:4571] |  |
| LINC00574 | 0,00 | 6,36 | -5,74 | 6 | 169790321 | 169802873 | + | 2318 | long intergenic non-protein coding RNA 574 [Source:HGNC Symbol;Acc:HGNC:21598] |  |
| CCDC85A | 7,55 | 0,00 | 5,97 | 2 | 56184123 | 56386173 | + | 3982 | coiled-coil domain containing 85A [Source:HGNC Symbol;Acc:HGNC:29400] |  |
| DDTL | 33,91 | 78,06 | -1,20 | 22 | 23966901 | 23972532 | + | 1545 | D-dopachrome tautomerase like [Source:HGNC Symbol;Acc:HGNC:33446] |  |
| YTHDF3-AS1 | 3,79 | 16,71 | -2,12 | 8 | 63167725 | 63168442 | - | 718 | YTHDF3 antisense RNA 1 (head to head) [Source:HGNC Symbol;Acc:HGNC:48728] |  |
| PROSER2-AS1 | 17,59 | 3,97 | 2,11 | 10 | 11849608 | 11894700 | - | 3274 | PROSER2 antisense RNA 1 [Source:HGNC Symbol;Acc:HGNC:27343] |  |
| CDH2 | 23,87 | 58,14 | -1,28 | 18 | 27950966 | 28177446 | - | 4950 | cadherin 2 [Source:HGNC Symbol;Acc:HGNC:1759] |  |
| FP565260,6 | 72,82 | 152,16 | -1,06 | 21 | 5155499 | 5165472 | - | 5411 | trafficking protein particle complex subunit 10-like [Source:NCBI gene;Acc:102724200] |  |
| AC023043,1 | 21,36 | 6,36 | 1,72 | 18 | 36179996 | 36187448 | - | 609 | uncharacterized LOC101927809 [Source:NCBI gene;Acc:101927809] |  |
| AL662797,1 | 21,36 | 6,36 | 1,72 | 6 | 30723105 | 30723877 | - | 773 | novel transcript, antisense to TUBB |  |
| AL137145,2 | 21,36 | 6,36 | 1,72 | 10 | 6277687 | 6335982 | + | 3086 | uncharacterized LOC399715 [Source:NCBI gene;Acc:399715] |  |
| CXCR4 | 97,92 | 199,17 | -1,02 | 2 | 136114349 | 136118165 | - | 2224 | C-X-C motif chemokine receptor 4 [Source:HGNC Symbol;Acc:HGNC:2561] |  |
| TSPAN8 | 16,34 | 42,21 | -1,37 | 12 | 71125085 | 71441898 | - | 2519 | tetraspanin 8 [Source:HGNC Symbol;Acc:HGNC:11855] |  |
| FGD3 | 23,87 | 7,15 | 1,72 | 9 | 92947451 | 93036236 | + | 5890 | FYVE, RhoGEF and PH domain containing 3 [Source:HGNC Symbol;Acc:HGNC:16027] |  |
| GPC2 | 6,30 | 22,29 | -1,81 | 7 | 100169606 | 100177372 | - | 3828 | glypican 2 [Source:HGNC Symbol;Acc:HGNC:4450] |  |
| HCN2 | 40,19 | 15,12 | 1,40 | 19 | 589893 | 617159 | + | 3408 | hyperpolarization activated cyclic nucleotide gated potassium and sodium channel 2 [Source:HGNC Symbol;Acc:HGNC:4846] |  |
| RPL37AP1 | 7,55 | 24,68 | -1,70 | 20 | 44466564 | 44466842 | - | 279 | ribosomal protein L37a pseudogene 1 [Source:HGNC Symbol;Acc:HGNC:16548] |  |
| DAPK1 | 8,81 | 26,27 | -1,57 | 9 | 87497228 | 87708633 | + | 8907 | death associated protein kinase 1 [Source:HGNC Symbol;Acc:HGNC:2674] |  |
| AC005077,4 | 11,32 | 32,65 | -1,52 | 7 | 76071469 | 76074963 | - | 3495 | hypothetical protein LOC285908 (LOC285908) pseudogene |  |
| USP2-AS1 | 30,14 | 10,34 | 1,53 | 11 | 119381778 | 119526664 | + | 4762 | USP2 antisense RNA 1 (head to head) [Source:HGNC Symbol;Acc:HGNC:48673] |  |
| VAC14-AS1 | 31,40 | 11,14 | 1,48 | 16 | 70755098 | 70773251 | + | 4853 | VAC14 antisense RNA 1 [Source:HGNC Symbol;Acc:HGNC:48605] |  |
| PHC1P1 | 31,40 | 11,93 | 1,38 | 12 | 55411727 | 55414787 | - | 3061 | polyhomeotic homolog 1 pseudogene 1 [Source:HGNC Symbol;Acc:HGNC:34502] |  |
| BX255923,1 | 10,06 | 30,26 | -1,58 | 9 | 41073710 | 41076392 | + | 681 | novel transcript |  |
| RINL | 12,57 | 2,37 | 2,33 | 19 | 38867834 | 38878279 | - | 4201 | Ras and Rab interactor like [Source:HGNC Symbol;Acc:HGNC:24795] |  |
| MMP25 | 1,28 | 11,93 | -3,13 | 16 | 3046681 | 3060726 | + | 4126 | matrix metallopeptidase 25 [Source:HGNC Symbol;Acc:HGNC:14246] |  |
| PIPOX | 1,28 | 11,93 | -3,13 | 17 | 28950513 | 29057220 | + | 4059 | pipecolic acid and sarcosine oxidase [Source:HGNC Symbol;Acc:HGNC:17804] |  |
| AC093388,1 | 12,57 | 2,37 | 2,33 | 2 | 190454092 | 190454521 | - | 430 | novel transcript, antisense to MFSD6 |  |
| AL355802,1 | 1,28 | 11,93 | -3,13 | 6 | 43538822 | 43539703 | - | 882 | 40S ribosomal protein S2 (RPS2) pseudogene |  |
| AL359183,1 | 12,57 | 2,37 | 2,33 | 10 | 71364243 | 71366374 | + | 2132 | TEC |  |
| AC138956,1 | 1,28 | 11,14 | -3,03 | 5 | 176347941 | 176353584 | + | 504 | novel transcript |  |
| AC009242,1 | 1,28 | 11,14 | -3,03 | 2 | 23667208 | 23685453 | - | 2706 | novel transcript |  |
| AL356801,1 | 12,57 | 2,37 | 2,33 | 14 | 55394940 | 55395233 | - | 294 | high mobility group nucleosome binding pseudogene |  |
| AL162615,1 | 1,28 | 11,93 | -3,13 | 20 | 49829125 | 49831085 | + | 1653 | cytokine-like nuclear factor n-pac (N-PAC) pseudogene |  |
| HSD17B13 | 1,28 | 11,14 | -3,03 | 4 | 87303789 | 87322906 | - | 2397 | hydroxysteroid 17-beta dehydrogenase 13 [Source:HGNC Symbol;Acc:HGNC:18685] |  |
| WNT8B | 12,57 | 2,37 | 2,33 | 10 | 100463041 | 100483744 | + | 2112 | Wnt family member 8B [Source:HGNC Symbol;Acc:HGNC:12789] |  |
| PCNPP3 | 1,28 | 11,14 | -3,03 | 12 | 65645992 | 65646462 | - | 471 | PEST containing nuclear protein pseudogene 3 [Source:HGNC Symbol;Acc:HGNC:41975] |  |
| AL772337,3 | 1,28 | 11,93 | -3,13 | 9 | 87864096 | 87866291 | + | 756 | cathepsin L (CTSL) pseudogene |  |
| AL049780,3 | 12,57 | 2,37 | 2,33 | 14 | 75011269 | 75012851 | - | 1583 | TEC |  |
| BMS1P13 | 1,28 | 11,93 | -3,13 | 9 | 65828120 | 65831696 | + | 885 | BMS1, ribosome biogenesis factor pseudogene 13 [Source:HGNC Symbol;Acc:HGNC:49158] |  |
| PROX2 | 1,28 | 11,14 | -3,03 | 14 | 74852871 | 74871940 | - | 4566 | prospero homeobox 2 [Source:HGNC Symbol;Acc:HGNC:26715] |  |
| RIMBP3C | 1,28 | 11,93 | -3,13 | 22 | 21545357 | 21551461 | - | 6105 | RIMS binding protein 3C [Source:HGNC Symbol;Acc:HGNC:33892] |  |
| SLC2A5 | 74,07 | 33,45 | 1,14 | 1 | 9035107 | 9088478 | - | 6693 | solute carrier family 2 member 5 [Source:HGNC Symbol;Acc:HGNC:11010] |  |
| NOD2 | 164,44 | 81,25 | 1,02 | 16 | 50693588 | 50734041 | + | 6873 | nucleotide binding oligomerization domain containing 2 [Source:HGNC Symbol;Acc:HGNC:5331] |  |
| TRPM2 | 48,97 | 20,70 | 1,24 | 21 | 44350163 | 44443081 | + | 6705 | transient receptor potential cation channel subfamily M member 2 [Source:HGNC Symbol;Acc:HGNC:12339] |  |
| C15orf62 | 18,85 | 47,79 | -1,34 | 15 | 40770080 | 40772449 | + | 2370 | chromosome 15 open reading frame 62 [Source:HGNC Symbol;Acc:HGNC:34489] |  |
| FAM157B | 46,46 | 19,90 | 1,22 | 9 | 138217068 | 138253217 | + | 2073 | family with sequence similarity 157 member B [Source:HGNC Symbol;Acc:HGNC:34080] |  |
| UNC5B | 17,59 | 43,80 | -1,31 | 10 | 71212570 | 71302864 | + | 6841 | unc-5 netrin receptor B [Source:HGNC Symbol;Acc:HGNC:12568] |  |
| AP001619,1 | 15,08 | 3,17 | 2,20 | 21 | 41874756 | 41877613 | - | 447 | novel transcript |  |
| SPDYE2B | 2,53 | 13,53 | -2,38 | 7 | 102650325 | 102661398 | + | 2072 | speedy/RINGO cell cycle regulator family member E2B [Source:HGNC Symbol;Acc:HGNC:48334] |  |
| RN7SKP16 | 2,53 | 13,53 | -2,38 | 1 | 33336566 | 33336864 | - | 299 | RNA, 7SK small nuclear pseudogene 16 [Source:HGNC Symbol;Acc:HGNC:45740] |  |
| Z95331,1 | 16,34 | 41,41 | -1,34 | 22 | 45657019 | 45680130 | + | 23112 | novel transcript, sense overlapping ATXN10 |  |
| IFNAR2 | 114,24 | 55,75 | 1,03 | 21 | 33229901 | 33265675 | + | 4614 | interferon alpha and beta receptor subunit 2 [Source:HGNC Symbol;Acc:HGNC:5433] |  |
| LINC01481 | 31,40 | 69,30 | -1,14 | 12 | 69904033 | 70243360 | - | 1490 | long intergenic non-protein coding RNA 1481 [Source:NCBI gene;Acc:101928062] |  |
| KLHL30 | 3,79 | 15,92 | -2,05 | 2 | 238138722 | 238152947 | + | 3726 | kelch like family member 30 [Source:HGNC Symbol;Acc:HGNC:24770] |  |
| EAF2 | 16,34 | 4,76 | 1,75 | 3 | 121835183 | 121886526 | + | 1770 | ELL associated factor 2 [Source:HGNC Symbol;Acc:HGNC:23115] |  |
| SLC2A14 | 16,34 | 3,97 | 2,00 | 12 | 7812512 | 7891148 | - | 5575 | solute carrier family 2 member 14 [Source:HGNC Symbol;Acc:HGNC:18301] |  |
| EP300-AS1 | 3,79 | 15,92 | -2,05 | 22 | 41174591 | 41197456 | - | 457 | EP300 antisense RNA 1 [Source:HGNC Symbol;Acc:HGNC:50504] |  |
| AL157935,1 | 3,79 | 15,92 | -2,05 | 9 | 127934503 | 127940952 | + | 2520 | novel transcript |  |
| AC125437,1 | 16,34 | 4,76 | 1,75 | 18 | 79117207 | 79117920 | + | 714 | novel transcript, sense intronic to ATP9B |  |
| AC124242,1 | 3,79 | 15,92 | -2,05 | 8 | 18084689 | 18096394 | + | 2013 | uncharacterized LOC101929066 [Source:NCBI gene;Acc:101929066] |  |
| LINC00239 | 3,79 | 15,12 | -1,97 | 14 | 101730437 | 101732522 | + | 639 | long intergenic non-protein coding RNA 239 [Source:HGNC Symbol;Acc:HGNC:20119] |  |
| TCN1 | 16,34 | 3,97 | 2,00 | 11 | 59852800 | 59866575 | - | 1586 | transcobalamin 1 [Source:HGNC Symbol;Acc:HGNC:11652] |  |
| SPTLC1P1 | 16,34 | 3,97 | 2,00 | 10 | 31360955 | 31361215 | + | 261 | serine palmitoyltransferase long chain base subunit 1 pseudogene 1 [Source:HGNC Symbol;Acc:HGNC:39668] |  |
| TPRG1-AS1 | 3,79 | 15,12 | -1,97 | 3 | 188941715 | 188947639 | - | 697 | TPRG1 antisense RNA 1 [Source:HGNC Symbol;Acc:HGNC:42391] |  |
| SYN2 | 3,79 | 15,92 | -2,05 | 3 | 12004402 | 12191400 | + | 5683 | synapsin II [Source:HGNC Symbol;Acc:HGNC:11495] |  |
| AC103974,1 | 16,34 | 3,97 | 2,00 | 11 | 18706537 | 18740568 | + | 874 | novel transcript |  |
| AC080013,4 | 12,57 | 33,45 | -1,41 | 3 | 158693120 | 158693768 | - | 649 | novel transcript |  |
| AC145207,8 | 11,32 | 31,85 | -1,49 | 17 | 81922899 | 81924511 | + | 563 | novel transcript, antisense MAFG |  |
| AL391095,3 | 18,85 | 5,56 | 1,73 | 20 | 38418483 | 38419202 | - | 720 | novel transcript |  |
| ZNF876P | 28,89 | 10,34 | 1,47 | 4 | 212610 | 255985 | + | 2804 | zinc finger protein 876, pseudogene [Source:HGNC Symbol;Acc:HGNC:32472] |  |
| CLN3 | 26,38 | 9,54 | 1,45 | 16 | 28474111 | 28495575 | - | 9858 | CLN3, battenin [Source:HGNC Symbol;Acc:HGNC:2074] |  |
| MIR6753 | 8,81 | 25,48 | -1,52 | 11 | 68044794 | 68044957 | + | 164 | microRNA 6753 [Source:HGNC Symbol;Acc:HGNC:50255] |  |
| PSG8 | 26,38 | 9,54 | 1,45 | 19 | 42752686 | 42855691 | - | 4090 | pregnancy specific beta-1-glycoprotein 8 [Source:HGNC Symbol;Acc:HGNC:9525] |  |
| KLHL31 | 5,04 | 19,10 | -1,90 | 6 | 53647901 | 53665708 | - | 5743 | kelch like family member 31 [Source:HGNC Symbol;Acc:HGNC:21353] |  |
| AL513534,1 | 5,04 | 19,10 | -1,90 | 10 | 68698500 | 68700794 | + | 2295 | novel transcript, overlapping to TET1 |  |
| ABHD17AP1 | 5,04 | 19,10 | -1,90 | 1 | 148146395 | 148149566 | + | 933 | abhydrolase domain containing 17A pseudogene 1 [Source:HGNC Symbol;Acc:HGNC:28394] |  |
| AC245052,4 | 7,55 | 23,09 | -1,60 | 19 | 54119511 | 54125343 | - | 4158 | novel transcript |  |
| RPL18AP7 | 25,12 | 8,75 | 1,51 | 3 | 38526802 | 38527325 | - | 524 | ribosomal protein L18a pseudogene 7 [Source:HGNC Symbol;Acc:HGNC:35607] |  |
| PCSK6 | 6,30 | 21,49 | -1,76 | 15 | 101297142 | 101525202 | - | 13239 | proprotein convertase subtilisin/kexin type 6 [Source:HGNC Symbol;Acc:HGNC:8569] |  |
| ACAP2-IT1 | 22,61 | 7,15 | 1,64 | 3 | 195280723 | 195282741 | - | 690 | ACAP2 intronic transcript 1 [Source:HGNC Symbol;Acc:HGNC:41426] |  |
| RPL7P23 | 22,61 | 7,15 | 1,64 | 5 | 77582376 | 77583122 | + | 747 | ribosomal protein L7 pseudogene 23 [Source:HGNC Symbol;Acc:HGNC:35658] |  |
| BACH1-IT2 | 22,61 | 7,15 | 1,64 | 21 | 29370497 | 29373709 | + | 1291 | BACH1 intronic transcript 2 [Source:HGNC Symbol;Acc:HGNC:40007] |  |
| GPR162 | 6,30 | 21,49 | -1,76 | 12 | 6821545 | 6829972 | + | 3295 | G protein-coupled receptor 162 [Source:HGNC Symbol;Acc:HGNC:16693] |  |
| MYOM2 | 6,30 | 21,49 | -1,76 | 8 | 2045040 | 2165552 | + | 7062 | myomesin 2 [Source:HGNC Symbol;Acc:HGNC:7614] |  |
| AC002401,2 | 22,61 | 7,15 | 1,64 | 17 | 50100704 | 50101920 | - | 430 | novel transcript, antisense to PDK2 |  |
| C9orf84 | 74,07 | 35,04 | 1,08 | 9 | 111686173 | 111795008 | - | 6276 | chromosome 9 open reading frame 84 [Source:HGNC Symbol;Acc:HGNC:26535] |  |
| RN7SL431P | 10,06 | 0,78 | 3,47 | 1 | 154166245 | 154166549 | - | 305 | RNA, 7SL, cytoplasmic 431, pseudogene [Source:HGNC Symbol;Acc:HGNC:46447] |  |
| STPG3 | 10,06 | 0,78 | 3,47 | 9 | 137251261 | 137253483 | + | 1611 | sperm-tail PG-rich repeat containing 3 [Source:HGNC Symbol;Acc:HGNC:37285] |  |
| AC092017,1 | 10,06 | 0,78 | 3,47 | 1 | 211207239 | 211207897 | + | 659 | pseudogene similar to px19-like protein (PX19) |  |
| SPDYE16 | 10,06 | 0,78 | 3,47 | 7 | 76531319 | 76541459 | - | 3611 | speedy/RINGO cell cycle regulator family member E16 [Source:HGNC Symbol;Acc:HGNC:51512] |  |
| AC005225,2 | 10,06 | 0,78 | 3,47 | 14 | 73616700 | 73633941 | - | 919 | novel transcript, antisense to ACOT6 |  |
| GPC5 | 10,06 | 0,78 | 3,47 | 13 | 91398607 | 92873682 | + | 3544 | glypican 5 [Source:HGNC Symbol;Acc:HGNC:4453] |  |
| MTNR1A | 10,06 | 0,78 | 3,47 | 4 | 186533655 | 186555567 | - | 1289 | melatonin receptor 1A [Source:HGNC Symbol;Acc:HGNC:7463] |  |
| GIP | 10,06 | 0,78 | 3,47 | 17 | 48958554 | 48968596 | - | 716 | gastric inhibitory polypeptide [Source:HGNC Symbol;Acc:HGNC:4270] |  |
| NRTN | 28,89 | 64,52 | -1,16 | 19 | 5823802 | 5828324 | + | 1109 | neurturin [Source:HGNC Symbol;Acc:HGNC:8007] |  |
| BAIAP2L2 | 13,83 | 34,24 | -1,30 | 22 | 38084889 | 38110670 | - | 2146 | BAI1 associated protein 2 like 2 [Source:HGNC Symbol;Acc:HGNC:26203] |  |
| NPIPB3 | 72,82 | 34,24 | 1,08 | 16 | 21402237 | 21448567 | - | 4717 | nuclear pore complex interacting protein family member B3 [Source:HGNC Symbol;Acc:HGNC:28989] |  |
| COX20 | 20,10 | 46,99 | -1,22 | 1 | 244835322 | 244845057 | + | 4864 | cytochrome c oxidase assembly factor COX20 [Source:HGNC Symbol;Acc:HGNC:26970] |  |
| GPX1P1 | 86,62 | 43,01 | 1,01 | X | 13378735 | 13379340 | - | 606 | glutathione peroxidase pseudogene 1 [Source:HGNC Symbol;Acc:HGNC:4560] |  |
| CBWD4P | 45,21 | 19,90 | 1,18 | 9 | 65287914 | 65323015 | - | 655 | COBW domain containing 4 pseudogene [Source:HGNC Symbol;Acc:HGNC:18520] |  |
| PLCB4 | 0,00 | 5,56 | -5,55 | 20 | 9068763 | 9481242 | + | 6900 | phospholipase C beta 4 [Source:HGNC Symbol;Acc:HGNC:9059] |  |
| GRB14 | 6,30 | 0,00 | 5,71 | 2 | 164492812 | 164621848 | - | 2958 | growth factor receptor bound protein 14 [Source:HGNC Symbol;Acc:HGNC:4565] |  |
| SPATA6L | 0,00 | 5,56 | -5,55 | 9 | 4553386 | 4666674 | - | 7361 | spermatogenesis associated 6 like [Source:HGNC Symbol;Acc:HGNC:25472] |  |
| RPS6KA2-IT1 | 6,30 | 0,00 | 5,71 | 6 | 166460663 | 166465383 | - | 579 | RPS6KA2 intronic transcript 1 [Source:HGNC Symbol;Acc:HGNC:41378] |  |
| CIB2 | 0,00 | 5,56 | -5,55 | 15 | 78104606 | 78131544 | - | 2146 | calcium and integrin binding family member 2 [Source:HGNC Symbol;Acc:HGNC:24579] |  |
| PLGLB1 | 6,30 | 0,00 | 5,71 | 2 | 87002559 | 87021852 | - | 3220 | plasminogen-like B1 [Source:HGNC Symbol;Acc:HGNC:9072] |  |
| AC002470,1 | 0,00 | 5,56 | -5,55 | 22 | 20981361 | 20981755 | - | 395 | novel transcript |  |
| LINC01979 | 6,30 | 0,00 | 5,71 | 17 | 79915252 | 79926725 | - | 5271 | long intergenic non-protein coding RNA 1979 [Source:HGNC Symbol;Acc:HGNC:52807] |  |
| AC010501,1 | 0,00 | 5,56 | -5,55 | 5 | 74865893 | 74867854 | + | 1962 | uncharacterized LOC441086 [Source:NCBI gene;Acc:441086] |  |
| AC018410,2 | 0,00 | 5,56 | -5,55 | 11 | 47220218 | 47221751 | - | 610 | novel transcript |  |
| AC006539,2 | 0,00 | 5,56 | -5,55 | 19 | 20033444 | 20034332 | + | 572 | BCL2/adenovirus E1B 19kDa interacting protein 3 (BNIP3) pseudogene |  |
| AL358072,1 | 0,00 | 5,56 | -5,55 | 1 | 117364899 | 117365473 | - | 575 | novel transcript |  |
| AC007938,1 | 0,00 | 5,56 | -5,55 | 7 | 130481491 | 130484392 | - | 2902 | novel transcript |  |
| AC124276,1 | 0,00 | 5,56 | -5,55 | 11 | 12066929 | 12073014 | + | 899 | novel transcript |  |
| AC012640,1 | 0,00 | 5,56 | -5,55 | 5 | 10248325 | 10249915 | + | 688 | novel transcript |  |
| MIR3181 | 0,00 | 5,56 | -5,55 | 16 | 50742305 | 50742377 | + | 73 | microRNA 3181 [Source:HGNC Symbol;Acc:HGNC:38378] |  |
| AL513218,1 | 0,00 | 5,56 | -5,55 | 1 | 52353487 | 52353877 | + | 391 | novel transcript, antisense to CC2D1B |  |
| MT-TL2 | 0,00 | 5,56 | -5,55 | MT | 12266 | 12336 | + | 71 | mitochondrially encoded tRNA leucine 2 (CUN) [Source:HGNC Symbol;Acc:HGNC:7491] |  |
| MIR573 | 0,00 | 5,56 | -5,55 | 4 | 24520192 | 24520290 | - | 99 | microRNA 573 [Source:HGNC Symbol;Acc:HGNC:32829] |  |
| AL390955,2 | 6,30 | 0,00 | 5,71 | 6 | 157323964 | 157324477 | + | 514 | novel transcript |  |
| MMP21 | 6,30 | 0,00 | 5,71 | 10 | 125766453 | 125775821 | - | 1919 | matrix metallopeptidase 21 [Source:HGNC Symbol;Acc:HGNC:14357] |  |
| AC123768,2 | 6,30 | 0,00 | 5,71 | 15 | 32613733 | 32615111 | + | 732 | novel transcript |  |
| AL024508,1 | 0,00 | 5,56 | -5,55 | 6 | 136629172 | 136647999 | + | 505 | uncharacterized LOC101928461 [Source:NCBI gene;Acc:101928461] |  |
| RPL21P132 | 0,00 | 5,56 | -5,55 | X | 101339917 | 101340934 | + | 462 | ribosomal protein L21 pseudogene 132 [Source:HGNC Symbol;Acc:HGNC:35865] |  |
| AF131216,4 | 0,00 | 5,56 | -5,55 | 8 | 11283481 | 11285068 | - | 1588 | novel transcript, antisense to MTMR9 |  |
| RPS3AP38 | 0,00 | 5,56 | -5,55 | 10 | 67960702 | 67961464 | - | 763 | ribosomal protein S3a pseudogene 38 [Source:HGNC Symbol;Acc:HGNC:36145] |  |
| RF00003 | 0,00 | 5,56 | -5,55 | 1 | 148522601 | 148522765 | + | 165 |  |  |
| SUMO2P3 | 0,00 | 5,56 | -5,55 | 7 | 55732144 | 55732431 | + | 288 | SUMO2 pseudogene 3 [Source:HGNC Symbol;Acc:HGNC:39013] |  |
| AC068305,2 | 0,00 | 5,56 | -5,55 | 12 | 58920639 | 59064238 | + | 555 | uncharacterized LOC105369791 [Source:NCBI gene;Acc:105369791] |  |
| AC005197,1 | 6,30 | 0,00 | 5,71 | 19 | 18881680 | 18883376 | + | 683 | novel transcript, antisense to CERS1 |  |
| AC092634,4 | 6,30 | 0,00 | 5,71 | 7 | 63924787 | 63926306 | - | 876 | TP53 target 3 (TP53TG3) pseudogene |  |
| AL358781,2 | 0,00 | 5,56 | -5,55 | 9 | 131516558 | 131522229 | - | 473 | novel transcript |  |
| SAA2-SAA4 | 0,00 | 5,56 | -5,55 | 11 | 18231423 | 18248635 | - | 743 | SAA2-SAA4 readthrough [Source:HGNC Symbol;Acc:HGNC:39550] |  |
| RPS26P8 | 6,30 | 0,00 | 5,71 | 17 | 45608571 | 45608918 | + | 348 | ribosomal protein S26 pseudogene 8 [Source:HGNC Symbol;Acc:HGNC:31329] |  |
| AF038458,3 | 6,30 | 0,00 | 5,71 | 19 | 35947115 | 35959192 | + | 500 | novel transcript |  |
| PTPRC | 0,00 | 5,56 | -5,55 | 1 | 198638457 | 198757476 | + | 9168 | protein tyrosine phosphatase, receptor type C [Source:HGNC Symbol;Acc:HGNC:9666] |  |
| LIMD1-AS1 | 6,30 | 0,00 | 5,71 | 3 | 45679043 | 45689134 | - | 1651 | LIMD1 antisense RNA 1 [Source:HGNC Symbol;Acc:HGNC:44107] |  |
| LINC00535 | 0,00 | 5,56 | -5,55 | 8 | 93213302 | 93700433 | - | 2595 | long intergenic non-protein coding RNA 535 [Source:HGNC Symbol;Acc:HGNC:43644] |  |
| SRP14P2 | 0,00 | 5,56 | -5,55 | 11 | 93535468 | 93535816 | + | 349 | signal recognition particle 14 pseudogene 2 [Source:HGNC Symbol;Acc:HGNC:53768] |  |
| AC073569,1 | 0,00 | 5,56 | -5,55 | 12 | 79823778 | 79825496 | + | 667 | novel transcript, antisense to PPP1R12A |  |
| AC087392,4 | 0,00 | 5,56 | -5,55 | 17 | 789744 | 790525 | - | 486 | novel transcript, antisense to RNMTL1 |  |
| FAM83C-AS1 | 0,00 | 5,56 | -5,55 | 20 | 35285251 | 35285756 | + | 352 | FAM83C antisense RNA 1 [Source:HGNC Symbol;Acc:HGNC:16113] |  |
| AL161729,3 | 6,30 | 0,00 | 5,71 | 9 | 95406990 | 95407662 | - | 673 | novel transcript |  |
| AC112694,2 | 0,00 | 5,56 | -5,55 | 11 | 18601882 | 18602649 | + | 768 | novel transcript |  |
| TERF1P5 | 6,30 | 0,00 | 5,71 | 13 | 18680494 | 18681700 | + | 1207 | telomeric repeat binding factor 1 pseudogene 5 [Source:HGNC Symbol;Acc:HGNC:39686] |  |
| AL645924,2 | 0,00 | 5,56 | -5,55 | 4 | 2505081 | 2506279 | - | 900 | ribosomal protein S3A (RPS3A) pseudogene |  |
| AC091057,3 | 6,30 | 0,00 | 5,71 | 15 | 30616958 | 30617749 | + | 792 | novel transcript |  |
| NRN1 | 6,30 | 0,00 | 5,71 | 6 | 5997999 | 6007605 | - | 2328 | neuritin 1 [Source:HGNC Symbol;Acc:HGNC:17972] |  |
| RPL21P44 | 0,00 | 5,56 | -5,55 | 4 | 53986587 | 53987058 | - | 472 | ribosomal protein L21 pseudogene 44 [Source:HGNC Symbol;Acc:HGNC:33820] |  |
| PCDH10 | 0,00 | 5,56 | -5,55 | 4 | 133149315 | 133208606 | + | 10161 | protocadherin 10 [Source:HGNC Symbol;Acc:HGNC:13404] |  |
| RNU6-680P | 0,00 | 5,56 | -5,55 | 5 | 75709495 | 75709599 | - | 105 | RNA, U6 small nuclear 680, pseudogene [Source:HGNC Symbol;Acc:HGNC:47643] |  |
| CCT7P1 | 6,30 | 0,00 | 5,71 | 6 | 149879962 | 149881572 | + | 1611 | chaperonin containing TCP1 subunit 7 pseudogene 1 [Source:HGNC Symbol;Acc:HGNC:35149] |  |
| ADAMTS8 | 0,00 | 5,56 | -5,55 | 11 | 130404925 | 130428993 | - | 4523 | ADAM metallopeptidase with thrombospondin type 1 motif 8 [Source:HGNC Symbol;Acc:HGNC:224] |  |
| RSL24D1P8 | 0,00 | 5,56 | -5,55 | 19 | 12096424 | 12096915 | + | 492 | ribosomal L24 domain containing 1 pseudogene 8 [Source:HGNC Symbol;Acc:HGNC:37873] |  |
| CTBP2P8 | 0,00 | 5,56 | -5,55 | 1 | 68161761 | 68163090 | - | 1330 | C-terminal binding protein 2 pseudogene 8 [Source:HGNC Symbol;Acc:HGNC:45200] |  |
| LIMS1-AS1 | 6,30 | 0,00 | 5,71 | 2 | 108676795 | 108678601 | - | 477 | LIMS1 antisense RNA 1 [Source:HGNC Symbol;Acc:HGNC:40342] |  |
| SNORA74A | 0,00 | 5,56 | -5,55 | 5 | 139278781 | 139278978 | + | 198 | small nucleolar RNA, H/ACA box 74A [Source:HGNC Symbol;Acc:HGNC:10119] |  |
| OR52W1 | 0,00 | 5,56 | -5,55 | 11 | 6199146 | 6200259 | + | 1114 | olfactory receptor family 52 subfamily W member 1 [Source:HGNC Symbol;Acc:HGNC:15239] |  |
| TMEM72-AS1 | 0,00 | 5,56 | -5,55 | 10 | 44811024 | 44959689 | - | 3300 | TMEM72 antisense RNA 1 [Source:HGNC Symbol;Acc:HGNC:27349] |  |
| TPRKBP2 | 0,00 | 5,56 | -5,55 | 16 | 28111173 | 28111647 | - | 475 | TP53RK binding protein pseudogene 2 [Source:HGNC Symbol;Acc:HGNC:44944] |  |
| AC104794,1 | 0,00 | 5,56 | -5,55 | 2 | 10083781 | 10086101 | + | 312 | novel transcript |  |
| AL033519,3 | 0,00 | 5,56 | -5,55 | 6 | 35544632 | 35545669 | + | 595 | novel transcript |  |
| GSTA8P | 0,00 | 5,56 | -5,55 | 6 | 52687930 | 52703825 | - | 637 | glutathione S-transferase alpha 8, pseudogene [Source:HGNC Symbol;Acc:HGNC:49903] |  |
| CSMD3 | 0,00 | 5,56 | -5,55 | 8 | 112222928 | 113437099 | - | 20567 | CUB and Sushi multiple domains 3 [Source:HGNC Symbol;Acc:HGNC:19291] |  |
| KIF12 | 6,30 | 0,00 | 5,71 | 9 | 114086126 | 114099291 | - | 3036 | kinesin family member 12 [Source:HGNC Symbol;Acc:HGNC:21495] |  |
| AC008147,1 | 0,00 | 5,56 | -5,55 | 12 | 50219604 | 50229984 | + | 325 | novel transcript, antisense to LIMA1 |  |
| AC073487,1 | 6,30 | 0,00 | 5,71 | 12 | 55761550 | 55762628 | - | 529 | novel transcript, sense intronic to SARNP |  |
| ZMYND19P1 | 0,00 | 5,56 | -5,55 | 14 | 77696591 | 77697377 | + | 787 | zinc finger MYND-type containing 19 pseudogene 1 [Source:HGNC Symbol;Acc:HGNC:44540] |  |
| AC010422,3 | 6,30 | 0,00 | 5,71 | 19 | 12643831 | 12648397 | - | 838 | novel transcript |  |
| AC104771,1 | 6,30 | 0,00 | 5,71 | 4 | 77112495 | 77113458 | - | 964 | ubiquitin family domain containing 1 (UBFD1) pseudogene |  |
| AC080188,1 | 6,30 | 0,00 | 5,71 | 4 | 168828919 | 168832937 | - | 552 | novel transcript |  |
| TRBV12-3 | 0,00 | 5,56 | -5,55 | 7 | 142560423 | 142560931 | + | 409 | T cell receptor beta variable 12-3 [Source:HGNC Symbol;Acc:HGNC:12185] |  |
| FOXD4L4 | 0,00 | 5,56 | -5,55 | 9 | 65736555 | 65738784 | + | 2230 | forkhead box D4 like 4 [Source:HGNC Symbol;Acc:HGNC:23762] |  |
| CFHR1 | 0,00 | 5,56 | -5,55 | 1 | 196819757 | 196832189 | + | 2197 | complement factor H related 1 [Source:HGNC Symbol;Acc:HGNC:4888] |  |
| AC069431,1 | 0,00 | 5,56 | -5,55 | 3 | 182783236 | 182793394 | - | 1877 | novel transcript |  |
| AC026785,3 | 0,00 | 5,56 | -5,55 | 5 | 17369225 | 17375577 | - | 285 | novel transcript |  |
| AC007551,2 | 6,30 | 0,00 | 5,71 | 7 | 35847038 | 35847426 | - | 389 | gem (nuclear organelle) associated protein 7 (GEMIN7) pseudogene |  |
| KLRC1 | 6,30 | 0,00 | 5,71 | 12 | 10442264 | 10454685 | - | 2698 | killer cell lectin like receptor C1 [Source:HGNC Symbol;Acc:HGNC:6374] |  |
| AC090236,2 | 6,30 | 0,00 | 5,71 | 18 | 58371566 | 58372666 | + | 1101 | novel transcript, sense intronic to NEDD4L |  |
| RPL23AP29 | 6,30 | 0,00 | 5,71 | 2 | 153370612 | 153371064 | + | 453 | ribosomal protein L23a pseudogene 29 [Source:HGNC Symbol;Acc:HGNC:36355] |  |
| BTG4P1 | 6,30 | 0,00 | 5,71 | 5 | 24786842 | 24787024 | - | 183 | BTG anti-proliferation factor 4 pseudogene 1 [Source:HGNC Symbol;Acc:HGNC:51544] |  |
| MIR4648 | 6,30 | 0,00 | 5,71 | 7 | 2527074 | 2527145 | + | 72 | microRNA 4648 [Source:HGNC Symbol;Acc:HGNC:41560] |  |
| LINC00632 | 6,30 | 0,00 | 5,71 | X | 140709562 | 140793215 | + | 28876 | long intergenic non-protein coding RNA 632 [Source:HGNC Symbol;Acc:HGNC:27865] |  |
| VN2R3P | 6,30 | 0,00 | 5,71 | 9 | 39016969 | 39039594 | + | 1412 | vomeronasal 2 receptor 3, pseudogene [Source:HGNC Symbol;Acc:HGNC:33209] |  |
| AC106869,1 | 30,14 | 11,93 | 1,32 | 2 | 47192405 | 47345074 | - | 3552 | uncharacterized LOC101927043 [Source:NCBI gene;Acc:101927043] |  |
| SLCO2A1 | 1,28 | 10,34 | -2,93 | 3 | 133928145 | 134052184 | - | 9834 | solute carrier organic anion transporter family member 2A1 [Source:HGNC Symbol;Acc:HGNC:10955] |  |
| GPR37L1 | 1,28 | 10,34 | -2,93 | 1 | 202122858 | 202133592 | + | 6588 | G protein-coupled receptor 37 like 1 [Source:HGNC Symbol;Acc:HGNC:14923] |  |
| KIRREL3 | 1,28 | 10,34 | -2,93 | 11 | 126423359 | 127003460 | - | 6594 | kirre like nephrin family adhesion molecule 3 [Source:HGNC Symbol;Acc:HGNC:23204] |  |
| SH3BGR | 11,32 | 2,37 | 2,18 | 21 | 39445855 | 39515506 | + | 1495 | SH3 domain binding glutamate rich protein [Source:HGNC Symbol;Acc:HGNC:10822] |  |
| AC018695,6 | 1,28 | 10,34 | -2,93 | 16 | 85697335 | 85697868 | - | 534 | novel transcript, sense intronic to C16orf74 |  |
| RPS6P25 | 11,32 | 2,37 | 2,18 | 19 | 12894133 | 12894880 | + | 748 | ribosomal protein S6 pseudogene 25 [Source:HGNC Symbol;Acc:HGNC:35948] |  |
| AC131011,1 | 1,28 | 10,34 | -2,93 | X | 23772992 | 23782956 | - | 672 | novel transcript |  |
| SNORD20 | 1,28 | 10,34 | -2,93 | 2 | 231456444 | 231456523 | - | 80 | small nucleolar RNA, C/D box 20 [Source:HGNC Symbol;Acc:HGNC:10143] |  |
| LILRA6 | 1,28 | 10,34 | -2,93 | 19 | 54236592 | 54242791 | - | 2718 | leukocyte immunoglobulin like receptor A6 [Source:HGNC Symbol;Acc:HGNC:15495] |  |
| AC091390,4 | 11,32 | 2,37 | 2,18 | 7 | 102364162 | 102380633 | + | 740 | uncharacterized LOC100289561 [Source:NCBI gene;Acc:100289561] |  |
| AC027796,2 | 1,28 | 10,34 | -2,93 | 17 | 3619256 | 3619913 | - | 344 | pterin-4 alpha-carbinolamine dehydratase/dimerization cofactor of hepatocyte nuclear factor pseudogene |  |
| AC084036,1 | 1,28 | 10,34 | -2,93 | 3 | 156523740 | 156524247 | - | 508 | novel transcript, antisense to KCNAB1 |  |
| AC006017,1 | 11,32 | 2,37 | 2,18 | 7 | 152120001 | 152121717 | - | 673 | novel transcript |  |
| AL160175,1 | 11,32 | 2,37 | 2,18 | 20 | 31808503 | 31808749 | - | 247 | ATP synthase, H+ transporting, mitochondrial F0 complex subunit f isoform 2 (ATP5J2) pseudogene |  |
| CLVS1 | 11,32 | 2,37 | 2,18 | 8 | 61057158 | 61501645 | + | 7393 | clavesin 1 [Source:HGNC Symbol;Acc:HGNC:23139] |  |
| AVPR2 | 1,28 | 10,34 | -2,93 | X | 153902531 | 153907166 | + | 2252 | arginine vasopressin receptor 2 [Source:HGNC Symbol;Acc:HGNC:897] |  |
| RMDN2-AS1 | 53,99 | 24,68 | 1,12 | 2 | 37949911 | 38067041 | - | 3282 | RMDN2 antisense RNA 1 [Source:HGNC Symbol;Acc:HGNC:41150] |  |
| MTCO2P16 | 27,63 | 10,34 | 1,40 | 2 | 201549631 | 201550307 | + | 677 | MT-CO2 pseudogene 16 [Source:HGNC Symbol;Acc:HGNC:52032] |  |
| PRSS27 | 16,34 | 39,82 | -1,28 | 16 | 2712418 | 2720551 | - | 5921 | serine protease 27 [Source:HGNC Symbol;Acc:HGNC:15475] |  |
| TMEM191C | 8,81 | 24,68 | -1,48 | 22 | 21466423 | 21471269 | + | 4299 | transmembrane protein 191C [Source:HGNC Symbol;Acc:HGNC:33601] |  |
| IRF6 | 7,55 | 22,29 | -1,55 | 1 | 209785623 | 209806175 | - | 4965 | interferon regulatory factor 6 [Source:HGNC Symbol;Acc:HGNC:6121] |  |
| ATP5PBP5 | 7,55 | 22,29 | -1,55 | 11 | 122960327 | 122961086 | + | 760 | ATP synthase peripheral stalk-membrane subunit b pseudogene 5 [Source:HGNC Symbol;Acc:HGNC:39746] |  |
| AP003072,2 | 23,87 | 7,95 | 1,57 | 11 | 93152075 | 93152750 | + | 676 | aminoacyl tRNA synthetase complex-interacting multifunctional protein 2 (AIMP2) pseudogene |  |
| AL356481,3 | 2,53 | 12,73 | -2,29 | 9 | 128630328 | 128631685 | - | 575 | novel transcript, antisense to SPTAN1 |  |
| AC003072,1 | 2,53 | 12,73 | -2,29 | 22 | 30653877 | 30654814 | - | 612 | ribosomal protein L13a (RPL13A) pseudogene |  |
| AC010271,1 | 2,53 | 12,73 | -2,29 | 19 | 40443436 | 40444087 | + | 558 | novel transcript, antisense to SERTAD3 |  |
| ZCWPW2 | 2,53 | 12,73 | -2,29 | 3 | 28349146 | 28538122 | + | 2895 | zinc finger CW-type and PWWP domain containing 2 [Source:HGNC Symbol;Acc:HGNC:23574] |  |
| AL158152,1 | 2,53 | 12,73 | -2,29 | 9 | 94176458 | 94177892 | + | 1435 | novel transcript |  |
| HMGA1P4 | 2,53 | 12,73 | -2,29 | 9 | 128663134 | 128663578 | - | 334 | high mobility group AT-hook 1 pseudogene 4 [Source:HGNC Symbol;Acc:HGNC:39093] |  |
| RF00410 | 13,83 | 3,17 | 2,07 | 6 | 149594625 | 149594759 | + | 135 |  |  |
| UBE2E1-AS1 | 2,53 | 12,73 | -2,29 | 3 | 23804024 | 23806905 | - | 778 | UBE2E1 antisense RNA 1 [Source:HGNC Symbol;Acc:HGNC:40598] |  |
| AC021205,3 | 13,83 | 3,17 | 2,07 | 4 | 122881878 | 122884712 | - | 1709 | novel transcript, antisense to FGF2 |  |
| AC008035,1 | 13,83 | 3,17 | 2,07 | 12 | 46537502 | 46652550 | + | 448 | novel transcript |  |
| GPM6A | 2,53 | 12,73 | -2,29 | 4 | 175632934 | 176002664 | - | 7040 | glycoprotein M6A [Source:HGNC Symbol;Acc:HGNC:4460] |  |
| AC017033,1 | 2,53 | 12,73 | -2,29 | 2 | 120866378 | 120867403 | - | 708 | novel transcript |  |
| RETREG1 | 36,42 | 15,12 | 1,26 | 5 | 16473038 | 16617058 | - | 4568 | reticulophagy regulator 1 [Source:HGNC Symbol;Acc:HGNC:25964] |  |
| SPTB | 5,04 | 18,31 | -1,84 | 14 | 64746283 | 64879907 | - | 14796 | spectrin beta, erythrocytic [Source:HGNC Symbol;Acc:HGNC:11274] |  |
| LPAR5 | 5,04 | 18,31 | -1,84 | 12 | 6618835 | 6636447 | - | 3258 | lysophosphatidic acid receptor 5 [Source:HGNC Symbol;Acc:HGNC:13307] |  |
| C6orf99 | 5,04 | 18,31 | -1,84 | 6 | 158869939 | 158919105 | + | 1614 | chromosome 6 open reading frame 99 [Source:HGNC Symbol;Acc:HGNC:21179] |  |
| AC133644,3 | 5,04 | 18,31 | -1,84 | 2 | 87455476 | 87767359 | + | 3849 | novel transcript |  |
| AC093525,4 | 5,04 | 18,31 | -1,84 | 16 | 2569043 | 2571936 | - | 459 | novel transcript, antisense to PDPK1 |  |
| RIMBP3B | 20,10 | 6,36 | 1,64 | 22 | 21383374 | 21389478 | + | 6105 | RIMS binding protein 3B [Source:HGNC Symbol;Acc:HGNC:33891] |  |
| AP000553,1 | 3,79 | 14,32 | -1,90 | 22 | 21652270 | 21670237 | + | 1093 | novel transcript |  |
| MYCL | 3,79 | 14,32 | -1,90 | 1 | 39895426 | 39902256 | - | 5063 | MYCL proto-oncogene, bHLH transcription factor [Source:HGNC Symbol;Acc:HGNC:7555] |  |
| AC124947,2 | 15,08 | 4,76 | 1,63 | 12 | 93314809 | 93315941 | + | 1133 | anaphase promoting complex subunit 10 (ANAPC10) pseudogene |  |
| MT-TG | 3,79 | 14,32 | -1,90 | MT | 9991 | 10058 | + | 68 | mitochondrially encoded tRNA glycine [Source:HGNC Symbol;Acc:HGNC:7486] |  |
| AL359715,2 | 3,79 | 14,32 | -1,90 | 6 | 80466958 | 80469080 | + | 2123 | novel transcript |  |
| MTND4P12 | 3,79 | 14,32 | -1,90 | 5 | 134926660 | 134928036 | - | 1377 | MT-ND4 pseudogene 12 [Source:HGNC Symbol;Acc:HGNC:42199] |  |
| AC107032,2 | 15,08 | 3,97 | 1,89 | 12 | 76562294 | 76615567 | + | 386 | uncharacterized LOC105369850 [Source:NCBI gene;Acc:105369850] |  |
| MEI1 | 3,79 | 14,32 | -1,90 | 22 | 41699499 | 41799456 | + | 5393 | meiotic double-stranded break formation protein 1 [Source:HGNC Symbol;Acc:HGNC:28613] |  |
| AP001767,4 | 15,08 | 4,76 | 1,63 | 11 | 83083687 | 83084138 | + | 452 | TEC |  |
| ATP6V1B1-AS1 | 15,08 | 4,76 | 1,63 | 2 | 70941817 | 70948610 | - | 1139 | ATP6V1B1 antisense RNA 1 [Source:HGNC Symbol;Acc:HGNC:51118] |  |
| AC009962,1 | 15,08 | 3,97 | 1,89 | 2 | 181887851 | 181891663 | - | 3813 | novel transcript, antisense to SSFA2 |  |
| HIF1A-AS1 | 15,08 | 3,97 | 1,89 | 14 | 61681041 | 61695823 | - | 652 | HIF1A antisense RNA 1 [Source:HGNC Symbol;Acc:HGNC:43014] |  |
| AC096642,1 | 15,08 | 4,76 | 1,63 | 1 | 219270774 | 219273387 | + | 2614 | novel transcript |  |
| CTLA4 | 3,79 | 14,32 | -1,90 | 2 | 203867771 | 203873965 | + | 2140 | cytotoxic T-lymphocyte associated protein 4 [Source:HGNC Symbol;Acc:HGNC:2505] |  |
| SOX15 | 48,97 | 98,78 | -1,01 | 17 | 7588178 | 7590170 | - | 1541 | SRY-box 15 [Source:HGNC Symbol;Acc:HGNC:11196] |  |
| NRG2 | 17,59 | 5,56 | 1,63 | 5 | 139846779 | 140043299 | - | 4520 | neuregulin 2 [Source:HGNC Symbol;Acc:HGNC:7998] |  |
| AL031281,2 | 5,04 | 16,71 | -1,71 | 1 | 22025511 | 22101360 | + | 10598 | novel protein, identical to cell division cycle 42 (GTP binding protein, 25kDa) CDC42 |  |
| AC004241,3 | 5,04 | 16,71 | -1,71 | 12 | 47699401 | 47699917 | - | 517 | novel transcript, sense intronic to RPAP3 |  |
| MIR3685 | 5,04 | 16,71 | -1,71 | 12 | 95309923 | 95309984 | + | 62 | microRNA 3685 [Source:HGNC Symbol;Acc:HGNC:38886] |  |
| FGF18 | 5,04 | 16,71 | -1,71 | 5 | 171419656 | 171457623 | + | 1986 | fibroblast growth factor 18 [Source:HGNC Symbol;Acc:HGNC:3674] |  |
| IMPDH1P8 | 5,04 | 16,71 | -1,71 | 3 | 15878047 | 15879571 | + | 1525 | inosine monophosphate dehydrogenase 1 pseudogene 8 [Source:HGNC Symbol;Acc:HGNC:33963] |  |
| MIR210 | 17,59 | 5,56 | 1,63 | 11 | 568089 | 568198 | - | 110 | microRNA 210 [Source:HGNC Symbol;Acc:HGNC:31587] |  |
| OMG | 5,04 | 16,71 | -1,71 | 17 | 31272013 | 31297539 | - | 3837 | oligodendrocyte myelin glycoprotein [Source:HGNC Symbol;Acc:HGNC:8135] |  |
| SOWAHD | 35,16 | 14,32 | 1,29 | X | 119758613 | 119760164 | + | 1552 | sosondowah ankyrin repeat domain family member D [Source:HGNC Symbol;Acc:HGNC:32960] |  |
| DET1 | 35,16 | 14,32 | 1,29 | 15 | 88494440 | 88546675 | - | 6853 | DET1, COP1 ubiquitin ligase partner [Source:HGNC Symbol;Acc:HGNC:25477] |  |
| NOX1 | 12,57 | 31,06 | -1,30 | X | 100843324 | 100874345 | - | 2529 | NADPH oxidase 1 [Source:HGNC Symbol;Acc:HGNC:7889] |  |
| AL365217,1 | 32,65 | 13,53 | 1,26 | 6 | 65302522 | 65304957 | + | 2436 | zinc finger CCCH-type containing 11A (ZC3H11A) pseudogene |  |
