# Supplementary material for: Sulfatide Acts as a Regulatory Molecule Controlling β1 Integrin–STAT5 Signaling and BOLA2-Dependent Apoptotic Pathway in Breast Cancer Cells
Source: Int J Mol Sci. 2025 Dec 9;26(24):11873. doi: 10.3390/ijms262411873 (PMC12733076; doi:10.3390/ijms262411873)
Supplement: Supplementary file 1 [file ijms-26-11873-s001.zip › Table S3.docx]

| gene_name | MDA468.CST | MDA468.C | log2FoldChange | Gene  chr | Gene  start | Gene  end | Gene  strand | Gene  length | gene_description |
| --- | --- | --- | --- | --- | --- | --- | --- | --- | --- |
| GAL3ST1 | 98319,04 | 2,26 | 15,34 | 22 | 30554635 | 30574587 | - | 3017 | galactose-3-O-sulfotransferase 1 [Source:HGNC Symbol;Acc:HGNC:24240] |
| GREM1 | 341,53 | 13,50 | 4,65 | 15 | 32717974 | 32745107 | + | 14606 | gremlin 1, DAN family BMP antagonist [Source:HGNC Symbol;Acc:HGNC:2001] |
| KRT34 | 432,26 | 25,87 | 4,06 | 17 | 41377650 | 41382403 | - | 1751 | keratin 34 [Source:HGNC Symbol;Acc:HGNC:6452] |
| KRT6A | 1930,06 | 167,53 | 3,53 | 12 | 52487174 | 52493257 | - | 2928 | keratin 6A [Source:HGNC Symbol;Acc:HGNC:6443] |
| KRT13 | 89,82 | 1,13 | 6,17 | 17 | 41500981 | 41505705 | - | 2913 | keratin 13 [Source:HGNC Symbol;Acc:HGNC:6415] |
| TP63 | 568,34 | 57,35 | 3,31 | 3 | 189631416 | 189897279 | + | 7454 | tumor protein p63 [Source:HGNC Symbol;Acc:HGNC:15979] |
| SPOCD1 | 88,04 | 2,26 | 5,22 | 1 | 31790422 | 31816051 | - | 7085 | SPOC domain containing 1 [Source:HGNC Symbol;Acc:HGNC:26338] |
| MAOA | 164,54 | 12,38 | 3,72 | X | 43654907 | 43746824 | + | 5528 | monoamine oxidase A [Source:HGNC Symbol;Acc:HGNC:6833] |
| LINC01187 | 85,38 | 2,26 | 5,17 | 5 | 170191579 | 170199141 | - | 2314 | long intergenic non-protein coding RNA 1187 [Source:HGNC Symbol;Acc:HGNC:49575] |
| SPRR3 | 86,27 | 3,38 | 4,63 | 1 | 153001747 | 153003856 | + | 1441 | small proline rich protein 3 [Source:HGNC Symbol;Acc:HGNC:11268] |
| DOCK2 | 266,82 | 30,37 | 3,13 | 5 | 169637247 | 170083382 | + | 13210 | dedicator of cytokinesis 2 [Source:HGNC Symbol;Acc:HGNC:2988] |
| UGT1A7 | 119,18 | 7,88 | 3,90 | 2 | 233681938 | 233773299 | + | 4167 | UDP glucuronosyltransferase family 1 member A7 [Source:HGNC Symbol;Acc:HGNC:12539] |
| COL17A1 | 160,09 | 14,63 | 3,44 | 10 | 104031286 | 104085957 | - | 7066 | collagen type XVII alpha 1 chain [Source:HGNC Symbol;Acc:HGNC:2194] |
| INHBA | 5244,99 | 911,83 | 2,52 | 7 | 41667168 | 41705834 | - | 8302 | inhibin subunit beta A [Source:HGNC Symbol;Acc:HGNC:6066] |
| SERPINB7 | 1734,39 | 311,44 | 2,48 | 18 | 63752935 | 63805376 | + | 2637 | serpin family B member 7 [Source:HGNC Symbol;Acc:HGNC:13902] |
| HMGA2 | 123,62 | 14,63 | 3,07 | 12 | 65824131 | 65966295 | + | 15403 | high mobility group AT-hook 2 [Source:HGNC Symbol;Acc:HGNC:5009] |
| HEPHL1 | 216,12 | 33,74 | 2,68 | 11 | 94021361 | 94113751 | + | 5345 | hephaestin like 1 [Source:HGNC Symbol;Acc:HGNC:30477] |
| SPRR1B | 102,28 | 11,25 | 3,17 | 1 | 153031202 | 153032900 | + | 620 | small proline rich protein 1B [Source:HGNC Symbol;Acc:HGNC:11260] |
| IL1R2 | 79,15 | 7,88 | 3,31 | 2 | 101991844 | 102028544 | + | 3073 | interleukin 1 receptor type 2 [Source:HGNC Symbol;Acc:HGNC:5994] |
| CLSTN2 | 155,64 | 23,62 | 2,71 | 3 | 139935185 | 140577397 | + | 14596 | calsyntenin 2 [Source:HGNC Symbol;Acc:HGNC:17448] |
| MAGEC1 | 122,73 | 16,87 | 2,85 | X | 141903894 | 141909388 | + | 4270 | MAGE family member C1 [Source:HGNC Symbol;Acc:HGNC:6812] |
| MOGAT2 | 233,91 | 41,61 | 2,49 | 11 | 75717819 | 75732958 | + | 4139 | monoacylglycerol O-acyltransferase 2 [Source:HGNC Symbol;Acc:HGNC:23248] |
| LINC02009 | 40,90 | 1,13 | 5,04 | 3 | 46416524 | 46423591 | - | 2951 | long intergenic non-protein coding RNA 2009 [Source:HGNC Symbol;Acc:HGNC:52845] |
| SERPINB3 | 253,48 | 48,36 | 2,39 | 18 | 63655197 | 63661963 | - | 1777 | serpin family B member 3 [Source:HGNC Symbol;Acc:HGNC:10569] |
| TMPRSS11E | 1102,89 | 250,73 | 2,14 | 4 | 68447449 | 68497604 | + | 2138 | transmembrane serine protease 11E [Source:HGNC Symbol;Acc:HGNC:24465] |
| CP | 779,14 | 3320,10 | -2,09 | 3 | 149162410 | 149222055 | - | 7296 | ceruloplasmin [Source:HGNC Symbol;Acc:HGNC:2295] |
| SOSTDC1 | 9,77 | 75,34 | -2,93 | 7 | 16461481 | 16530580 | - | 2473 | sclerostin domain containing 1 [Source:HGNC Symbol;Acc:HGNC:21748] |
| OLFML3 | 4183,00 | 999,52 | 2,07 | 1 | 113979391 | 114035572 | + | 2821 | olfactomedin like 3 [Source:HGNC Symbol;Acc:HGNC:24956] |
| KRT16P6 | 1752,18 | 412,63 | 2,09 | 17 | 16817983 | 16822579 | - | 1906 | keratin 16 pseudogene 6 [Source:HGNC Symbol;Acc:HGNC:50719] |
| DNER | 187,66 | 34,86 | 2,42 | 2 | 229357629 | 229714558 | - | 3935 | delta/notch like EGF repeat containing [Source:HGNC Symbol;Acc:HGNC:24456] |
| FAM156A | 25,78 | 139,42 | -2,43 | X | 52926402 | 52995472 | - | 6608 | family with sequence similarity 156 member A [Source:HGNC Symbol;Acc:HGNC:30114] |
| AP001596.1 | 85,38 | 12,38 | 2,78 | 21 | 26378552 | 26471698 | + | 2247 | novel transcript |
| SERPINB4 | 274,83 | 60,72 | 2,18 | 18 | 63637259 | 63644298 | - | 2013 | serpin family B member 4 [Source:HGNC Symbol;Acc:HGNC:10570] |
| BOLA2B | 299,73 | 1207,52 | -2,01 | 16 | 30192934 | 30194306 | - | 1365 | bolA family member 2B [Source:HGNC Symbol;Acc:HGNC:32479] |
| AL590004.3 | 723,99 | 175,40 | 2,04 | 6 | 3904920 | 3911979 | - | 7060 | novel transcript |
| LNCAROD | 203,67 | 42,73 | 2,25 | 10 | 52450874 | 52755409 | - | 2007 | lncRNA activating regulator of DKK1 [Source:HGNC Symbol;Acc:HGNC:50913] |
| MROH2B | 23,12 | 0,00 | 7,55 | 5 | 40998017 | 41071342 | - | 6085 | maestro heat like repeat family member 2B [Source:HGNC Symbol;Acc:HGNC:26857] |
| NOV | 33,79 | 1,13 | 4,76 | 8 | 119416306 | 119424353 | + | 2673 | nephroblastoma overexpressed [Source:HGNC Symbol;Acc:HGNC:7885] |
| PRR9 | 22,23 | 0,00 | 7,49 | 1 | 153217584 | 153219317 | + | 1229 | proline rich 9 [Source:HGNC Symbol;Acc:HGNC:32057] |
| HSH2D | 5,33 | 47,23 | -3,12 | 19 | 16134028 | 16158575 | + | 3398 | hematopoietic SH2 domain containing [Source:HGNC Symbol;Acc:HGNC:24920] |
| FLG | 41,79 | 3,38 | 3,58 | 1 | 152302175 | 152325203 | - | 12747 | filaggrin [Source:HGNC Symbol;Acc:HGNC:3748] |
| SPRR2A | 64,92 | 9,00 | 2,83 | 1 | 153056113 | 153057537 | - | 709 | small proline rich protein 2A [Source:HGNC Symbol;Acc:HGNC:11261] |
| AP005230.1 | 105,83 | 20,25 | 2,38 | 18 | 1883524 | 2489426 | - | 3798 | novel transcript |
| PLA2G2A | 112,95 | 22,50 | 2,32 | 1 | 19975431 | 19980416 | - | 1685 | phospholipase A2 group IIA [Source:HGNC Symbol;Acc:HGNC:9031] |
| COL15A1 | 87,16 | 15,75 | 2,46 | 9 | 98943179 | 99070792 | + | 6653 | collagen type XV alpha 1 chain [Source:HGNC Symbol;Acc:HGNC:2192] |
| PCDH19 | 135,18 | 28,12 | 2,26 | X | 100291644 | 100410273 | - | 9756 | protocadherin 19 [Source:HGNC Symbol;Acc:HGNC:14270] |
| PTGFR | 26,67 | 1,13 | 4,42 | 1 | 78303884 | 78539749 | + | 4714 | prostaglandin F receptor [Source:HGNC Symbol;Acc:HGNC:9600] |
| LINC01127 | 26,67 | 1,13 | 4,42 | 2 | 101962056 | 101987167 | + | 3841 | long intergenic non-protein coding RNA 1127 [Source:HGNC Symbol;Acc:HGNC:49292] |
| C8orf31 | 29,34 | 134,93 | -2,20 | 8 | 143039209 | 143059942 | + | 2929 | chromosome 8 open reading frame 31 (putative) [Source:HGNC Symbol;Acc:HGNC:26731] |
| CST6 | 418,03 | 107,94 | 1,95 | 11 | 66011841 | 66013505 | + | 759 | cystatin E/M [Source:HGNC Symbol;Acc:HGNC:2478] |
| NLRP10 | 51,58 | 6,76 | 2,91 | 11 | 7959424 | 7965426 | - | 2531 | NLR family pyrin domain containing 10 [Source:HGNC Symbol;Acc:HGNC:21464] |
| AC008063.2 | 49,80 | 6,76 | 2,86 | 2 | 162092232 | 162095032 | + | 1117 | novel transcript |
| HKDC1 | 45,35 | 5,63 | 2,98 | 10 | 69220303 | 69267559 | + | 4259 | hexokinase domain containing 1 [Source:HGNC Symbol;Acc:HGNC:23302] |
| TNC | 1203,40 | 345,17 | 1,80 | 9 | 115019578 | 115118257 | - | 9636 | tenascin C [Source:HGNC Symbol;Acc:HGNC:5318] |
| LINC01204 | 19,56 | 0,00 | 7,31 | X | 45505388 | 45630202 | + | 5260 | long intergenic non-protein coding RNA 1204 [Source:HGNC Symbol;Acc:HGNC:49635] |
| NXF3 | 19,56 | 0,00 | 7,31 | X | 103075810 | 103093125 | - | 3526 | nuclear RNA export factor 3 [Source:HGNC Symbol;Acc:HGNC:8073] |
| RNF150 | 19,56 | 0,00 | 7,31 | 4 | 140859807 | 141212877 | - | 11007 | ring finger protein 150 [Source:HGNC Symbol;Acc:HGNC:23138] |
| IL1RL1 | 52,47 | 7,88 | 2,72 | 2 | 102311502 | 102352037 | + | 5746 | interleukin 1 receptor like 1 [Source:HGNC Symbol;Acc:HGNC:5998] |
| MANCR | 171,65 | 42,73 | 2,00 | 10 | 4650185 | 4678154 | - | 1920 | mitotically associated long non coding RNA [Source:HGNC Symbol;Acc:HGNC:44678] |
| ABCC2 | 344,20 | 94,45 | 1,86 | 10 | 99782640 | 99852594 | + | 7837 | ATP binding cassette subfamily C member 2 [Source:HGNC Symbol;Acc:HGNC:53] |
| C1QL2 | 51,58 | 7,88 | 2,69 | 2 | 119156243 | 119158889 | - | 2043 | complement C1q like 2 [Source:HGNC Symbol;Acc:HGNC:24181] |
| CORO6 | 249,03 | 66,34 | 1,91 | 17 | 29614756 | 29622907 | - | 4321 | coronin 6 [Source:HGNC Symbol;Acc:HGNC:21356] |
| PLEKHG4B | 133,41 | 462,10 | -1,79 | 5 | 92151 | 189972 | + | 13304 | pleckstrin homology and RhoGEF domain containing G4B [Source:HGNC Symbol;Acc:HGNC:29399] |
| LRRC31 | 70,26 | 13,50 | 2,37 | 3 | 169839179 | 169869930 | - | 3113 | leucine rich repeat containing 31 [Source:HGNC Symbol;Acc:HGNC:26261] |
| SLC7A8 | 6,22 | 43,86 | -2,79 | 14 | 23125295 | 23183674 | - | 5991 | solute carrier family 7 member 8 [Source:HGNC Symbol;Acc:HGNC:11066] |
| AC017104.1 | 5,33 | 39,36 | -2,85 | 2 | 231388976 | 231394991 | + | 1597 | novel transcript |
| NT5DC4 | 22,23 | 1,13 | 4,16 | 2 | 112721486 | 112742879 | + | 4225 | 5'-nucleotidase domain containing 4 [Source:HGNC Symbol;Acc:HGNC:27678] |
| CD14 | 409,13 | 1327,82 | -1,70 | 5 | 140631728 | 140633701 | - | 1886 | CD14 molecule [Source:HGNC Symbol;Acc:HGNC:1628] |
| MYLK | 497,18 | 147,29 | 1,75 | 3 | 123610049 | 123884331 | - | 22167 | myosin light chain kinase [Source:HGNC Symbol;Acc:HGNC:7590] |
| KLF17 | 34,68 | 3,38 | 3,32 | 1 | 44118850 | 44135140 | + | 3131 | Kruppel like factor 17 [Source:HGNC Symbol;Acc:HGNC:18830] |
| LGALS1 | 450,04 | 1452,62 | -1,69 | 22 | 37675608 | 37679806 | + | 1099 | galectin 1 [Source:HGNC Symbol;Acc:HGNC:6561] |
| LDHD | 27,56 | 112,44 | -2,02 | 16 | 75111860 | 75116771 | - | 2359 | lactate dehydrogenase D [Source:HGNC Symbol;Acc:HGNC:19708] |
| CAPN14 | 152,08 | 39,36 | 1,95 | 2 | 31173056 | 31233858 | - | 4014 | calpain 14 [Source:HGNC Symbol;Acc:HGNC:16664] |
| A2ML1 | 217,90 | 60,72 | 1,84 | 12 | 8822472 | 8887001 | + | 7303 | alpha-2-macroglobulin like 1 [Source:HGNC Symbol;Acc:HGNC:23336] |
| OASL | 35,57 | 137,18 | -1,94 | 12 | 121019111 | 121039242 | - | 3517 | 2'-5'-oligoadenylate synthetase like [Source:HGNC Symbol;Acc:HGNC:8090] |
| SERPINE1 | 105,83 | 25,87 | 2,03 | 7 | 101127089 | 101139266 | + | 3190 | serpin family E member 1 [Source:HGNC Symbol;Acc:HGNC:8583] |
| SLC37A2 | 183,21 | 50,60 | 1,85 | 11 | 125063067 | 125090312 | + | 6371 | solute carrier family 37 member 2 [Source:HGNC Symbol;Acc:HGNC:20644] |
| GPRC5D | 2,66 | 26,99 | -3,28 | 12 | 12940775 | 12952147 | - | 1124 | G protein-coupled receptor class C group 5 member D [Source:HGNC Symbol;Acc:HGNC:13310] |
| SLCO2A1 | 443,82 | 138,30 | 1,68 | 3 | 133928145 | 134052184 | - | 9834 | solute carrier organic anion transporter family member 2A1 [Source:HGNC Symbol;Acc:HGNC:10955] |
| AL355032.1 | 61,36 | 210,26 | -1,77 | 14 | 101677943 | 101678380 | + | 438 | ribosomal protein L26-like 1 (RPL26L1) pseudogene |
| AL033397.1 | 123,62 | 32,62 | 1,92 | 6 | 53561289 | 53617171 | - | 3994 | uncharacterized LOC101927136 [Source:NCBI gene;Acc:101927136] |
| NOX5 | 35,57 | 4,51 | 2,95 | 15 | 68930525 | 69062743 | + | 12511 | NADPH oxidase 5 [Source:HGNC Symbol;Acc:HGNC:14874] |
| MAL | 35,57 | 4,51 | 2,95 | 2 | 95025677 | 95053996 | + | 1457 | mal, T cell differentiation protein [Source:HGNC Symbol;Acc:HGNC:6817] |
| HTRA3 | 20,45 | 1,13 | 4,04 | 4 | 8269765 | 8307111 | + | 3321 | HtrA serine peptidase 3 [Source:HGNC Symbol;Acc:HGNC:30406] |
| SYT6 | 20,45 | 1,13 | 4,04 | 1 | 114089291 | 114153919 | - | 5104 | synaptotagmin 6 [Source:HGNC Symbol;Acc:HGNC:18638] |
| NPIPB13 | 38,24 | 5,63 | 2,74 | 16 | 30222937 | 30254510 | - | 5202 | nuclear pore complex interacting protein family, member B13 [Source:HGNC Symbol;Acc:HGNC:41989] |
| ELOVL6 | 306,85 | 94,45 | 1,70 | 4 | 110045846 | 110199199 | - | 7297 | ELOVL fatty acid elongase 6 [Source:HGNC Symbol;Acc:HGNC:15829] |
| TAGLN | 843,18 | 275,47 | 1,61 | 11 | 117199321 | 117204782 | + | 3917 | transgelin [Source:HGNC Symbol;Acc:HGNC:11553] |
| PAPPA | 91,60 | 22,50 | 2,02 | 9 | 116153804 | 116402322 | + | 11573 | pappalysin 1 [Source:HGNC Symbol;Acc:HGNC:8602] |
| SGK1 | 709,76 | 230,49 | 1,62 | 6 | 134169246 | 134318112 | - | 9992 | serum/glucocorticoid regulated kinase 1 [Source:HGNC Symbol;Acc:HGNC:10810] |
| MARCO | 80,93 | 19,12 | 2,08 | 2 | 118942166 | 118994660 | + | 2079 | macrophage receptor with collagenous structure [Source:HGNC Symbol;Acc:HGNC:6895] |
| PMS2P10 | 21,34 | 85,46 | -1,99 | 7 | 75288120 | 75299795 | - | 612 | PMS1 homolog 2, mismatch repair system component pseudogene 10 [Source:HGNC Symbol;Acc:HGNC:9124] |
| AC017100.1 | 27,56 | 2,26 | 3,55 | 18 | 31685655 | 31686823 | + | 1169 | novel transcript, antisense to B4GALT6 |
| SLC15A3 | 268,60 | 811,76 | -1,60 | 11 | 60937084 | 60952530 | - | 6118 | solute carrier family 15 member 3 [Source:HGNC Symbol;Acc:HGNC:18068] |
| SPCS2P4 | 103,17 | 327,18 | -1,66 | 1 | 28095742 | 28096422 | - | 681 | signal peptidase complex subunit 2 pseudogene 4 [Source:HGNC Symbol;Acc:HGNC:45237] |
| MILR1 | 53,36 | 10,13 | 2,38 | 17 | 64449037 | 64468643 | + | 1816 | mast cell immunoglobulin like receptor 1 [Source:HGNC Symbol;Acc:HGNC:27570] |
| KCNMB1 | 6,22 | 38,24 | -2,59 | 5 | 170374671 | 170389677 | - | 6079 | potassium calcium-activated channel subfamily M regulatory beta subunit 1 [Source:HGNC Symbol;Acc:HGNC:6285] |
| CORO2B | 19,56 | 1,13 | 3,98 | 15 | 68578969 | 68727806 | + | 3998 | coronin 2B [Source:HGNC Symbol;Acc:HGNC:2256] |
| AC135279.1 | 19,56 | 1,13 | 3,98 | 12 | 64442510 | 64443330 | - | 504 | mitochondrial ribosomal protein S25 (MRPS25) pseudogene |
| SERPINB2 | 386,01 | 124,81 | 1,63 | 18 | 63871692 | 63903890 | + | 2748 | serpin family B member 2 [Source:HGNC Symbol;Acc:HGNC:8584] |
| IGFBP5 | 5814,23 | 2000,16 | 1,54 | 2 | 216672105 | 216695525 | - | 6442 | insulin like growth factor binding protein 5 [Source:HGNC Symbol;Acc:HGNC:5474] |
| WFDC3 | 43,57 | 7,88 | 2,45 | 20 | 45747944 | 45791932 | - | 2405 | WAP four-disulfide core domain 3 [Source:HGNC Symbol;Acc:HGNC:15957] |
| THBS1 | 5085,78 | 1750,56 | 1,54 | 15 | 39581079 | 39599466 | + | 9158 | thrombospondin 1 [Source:HGNC Symbol;Acc:HGNC:11785] |
| COL5A2 | 756,90 | 2207,03 | -1,54 | 2 | 189031896 | 189225312 | - | 7453 | collagen type V alpha 2 chain [Source:HGNC Symbol;Acc:HGNC:2210] |
| AC104454.2 | 91,60 | 23,62 | 1,95 | 1 | 84038529 | 84065031 | + | 441 | novel transcript |
| MTX1P1 | 169,87 | 511,57 | -1,59 | 1 | 155230975 | 155234325 | + | 838 | metaxin 1 pseudogene 1 [Source:HGNC Symbol;Acc:HGNC:7505] |
| DUSP27 | 36,46 | 5,63 | 2,67 | 1 | 167094045 | 167129165 | + | 4304 | dual specificity phosphatase 27, atypical [Source:HGNC Symbol;Acc:HGNC:25034] |
| DIO2 | 308,62 | 98,95 | 1,64 | 14 | 80197527 | 80387757 | - | 7494 | iodothyronine deiodinase 2 [Source:HGNC Symbol;Acc:HGNC:2884] |
| LINC00629 | 14,22 | 0,00 | 6,85 | X | 134549973 | 134559923 | + | 909 | long intergenic non-protein coding RNA 629 [Source:HGNC Symbol;Acc:HGNC:44262] |
| RYR2 | 14,22 | 0,00 | 6,85 | 1 | 237042205 | 237833988 | + | 17777 | ryanodine receptor 2 [Source:HGNC Symbol;Acc:HGNC:10484] |
| ATP6V0D2 | 1549,39 | 536,31 | 1,53 | 8 | 85987323 | 86154228 | + | 3010 | ATPase H+ transporting V0 subunit d2 [Source:HGNC Symbol;Acc:HGNC:18266] |
| PCDHAC1 | 45,35 | 9,00 | 2,32 | 5 | 140926369 | 141012344 | + | 6943 | protocadherin alpha subfamily C, 1 [Source:HGNC Symbol;Acc:HGNC:8676] |
| AC093001.1 | 16,89 | 67,47 | -1,99 | 3 | 149284782 | 149333653 | + | 551 | novel transcript |
| NEXN | 164,54 | 49,48 | 1,73 | 1 | 77888513 | 77943895 | + | 4436 | nexilin F-actin binding protein [Source:HGNC Symbol;Acc:HGNC:29557] |
| ABCC6P1 | 25,78 | 93,33 | -1,85 | 16 | 18571162 | 18598328 | + | 3431 | ATP binding cassette subfamily C member 6 pseudogene 1 [Source:HGNC Symbol;Acc:HGNC:33352] |
| SERPINE2 | 642,16 | 218,13 | 1,56 | 2 | 223975112 | 224039319 | - | 8891 | serpin family E member 2 [Source:HGNC Symbol;Acc:HGNC:8951] |
| SEMA3E | 445,60 | 1274,98 | -1,52 | 7 | 83363238 | 83649113 | - | 8424 | semaphorin 3E [Source:HGNC Symbol;Acc:HGNC:10727] |
| AC009812.4 | 0,88 | 19,12 | -4,25 | 8 | 80541300 | 80543104 | + | 1805 | novel transcript |
| TMEM59L | 18,67 | 1,13 | 3,91 | 19 | 18607430 | 18621039 | + | 2626 | transmembrane protein 59 like [Source:HGNC Symbol;Acc:HGNC:13237] |
| HGD | 59,58 | 13,50 | 2,13 | 3 | 120628173 | 120682571 | - | 2983 | homogentisate 1,2-dioxygenase [Source:HGNC Symbol;Acc:HGNC:4892] |
| HIST3H2A | 510,53 | 1447,00 | -1,50 | 1 | 228456979 | 228457873 | - | 895 | histone cluster 3 H2A [Source:HGNC Symbol;Acc:HGNC:20507] |
| AL157700.1 | 3,55 | 26,99 | -2,88 | X | 68013470 | 68014901 | - | 1432 | novel transcript |
| AL391427.1 | 175,21 | 53,98 | 1,70 | 10 | 4995488 | 4997380 | + | 482 | uncharacterized LOC101928051 [Source:NCBI gene;Acc:101928051] |
| CDH23 | 115,62 | 344,05 | -1,57 | 10 | 71396934 | 71815947 | + | 18587 | cadherin related 23 [Source:HGNC Symbol;Acc:HGNC:13733] |
| GPAT3 | 124,51 | 35,99 | 1,79 | 4 | 83535914 | 83605875 | + | 3468 | glycerol-3-phosphate acyltransferase 3 [Source:HGNC Symbol;Acc:HGNC:28157] |
| LINC02167 | 181,44 | 526,19 | -1,54 | 16 | 35743268 | 35756515 | + | 1989 | long intergenic non-protein coding RNA 2167 [Source:HGNC Symbol;Acc:HGNC:53028] |
| LINC00707 | 204,56 | 65,22 | 1,65 | 10 | 6779549 | 6879450 | + | 9833 | long intergenic non-protein coding RNA 707 [Source:HGNC Symbol;Acc:HGNC:44691] |
| CLCA2 | 666,18 | 233,87 | 1,51 | 1 | 86424086 | 86456558 | + | 4496 | chloride channel accessory 2 [Source:HGNC Symbol;Acc:HGNC:2016] |
| LINC00862 | 106,72 | 30,37 | 1,81 | 1 | 200253419 | 200400705 | - | 3174 | long intergenic non-protein coding RNA 862 [Source:HGNC Symbol;Acc:HGNC:21901] |
| NKX2-5 | 1,77 | 22,50 | -3,57 | 5 | 173232109 | 173235357 | - | 2094 | NK2 homeobox 5 [Source:HGNC Symbol;Acc:HGNC:2488] |
| TP53AIP1 | 2,66 | 22,50 | -3,02 | 11 | 128934731 | 128943399 | - | 3445 | tumor protein p53 regulated apoptosis inducing protein 1 [Source:HGNC Symbol;Acc:HGNC:29984] |
| SYPL1P2 | 13,33 | 0,00 | 6,76 | 17 | 27351858 | 27352584 | - | 727 | synaptophysin like 1 pseudogene 2 [Source:HGNC Symbol;Acc:HGNC:53547] |
| HSPA6 | 13,33 | 0,00 | 6,76 | 1 | 161524540 | 161526910 | + | 2371 | heat shock protein family A (Hsp70) member 6 [Source:HGNC Symbol;Acc:HGNC:5239] |
| AL592437.2 | 13,33 | 0,00 | 6,76 | 9 | 105758164 | 105760755 | + | 2434 | DEP domain containing 1 (DEPDC1) pseudogene |
| AC109635.4 | 13,33 | 0,00 | 6,76 | 11 | 50279828 | 50298452 | - | 1005 | septin 7 pseudogene [Source:NCBI gene;Acc:441601] |
| CBSL | 499,85 | 1380,66 | -1,47 | 21 | 6444869 | 6468040 | - | 4004 | cystathionine-beta-synthase like [Source:HGNC Symbol;Acc:HGNC:51829] |
| SERPINA1 | 99,61 | 28,12 | 1,82 | 14 | 94376747 | 94390693 | - | 4902 | serpin family A member 1 [Source:HGNC Symbol;Acc:HGNC:8941] |
| DHRS9 | 17,78 | 1,13 | 3,84 | 2 | 169064789 | 169096167 | + | 3750 | dehydrogenase/reductase 9 [Source:HGNC Symbol;Acc:HGNC:16888] |
| AHNAK2 | 2100,84 | 767,91 | 1,45 | 14 | 104937244 | 104978357 | - | 18771 | AHNAK nucleoprotein 2 [Source:HGNC Symbol;Acc:HGNC:20125] |
| NCR3LG1 | 51,58 | 11,25 | 2,19 | 11 | 17351726 | 17377341 | + | 6438 | natural killer cell cytotoxicity receptor 3 ligand 1 [Source:HGNC Symbol;Acc:HGNC:42400] |
| IFITM1 | 338,87 | 936,56 | -1,47 | 11 | 313506 | 315272 | + | 1080 | interferon induced transmembrane protein 1 [Source:HGNC Symbol;Acc:HGNC:5412] |
| AADACL3 | 48,02 | 10,13 | 2,23 | 1 | 12716115 | 12728759 | + | 4049 | arylacetamide deacetylase like 3 [Source:HGNC Symbol;Acc:HGNC:32037] |
| FRMPD1 | 666,18 | 238,36 | 1,48 | 9 | 37651000 | 37746904 | + | 5867 | FERM and PDZ domain containing 1 [Source:HGNC Symbol;Acc:HGNC:29159] |
| PARM1 | 223,24 | 74,21 | 1,59 | 4 | 74933095 | 75050115 | + | 5034 | prostate androgen-regulated mucin-like protein 1 [Source:HGNC Symbol;Acc:HGNC:24536] |
| TRPM6 | 5,33 | 31,49 | -2,53 | 9 | 74722495 | 74888094 | - | 9193 | transient receptor potential cation channel subfamily M member 6 [Source:HGNC Symbol;Acc:HGNC:17995] |
| NMRAL2P | 33,79 | 5,63 | 2,56 | 3 | 185959943 | 185980872 | + | 2628 | NmrA like redox sensor 2, pseudogene [Source:HGNC Symbol;Acc:HGNC:52332] |
| ACTG1P10 | 6,22 | 34,86 | -2,46 | X | 53142832 | 53143913 | - | 1082 | actin gamma 1 pseudogene 10 [Source:HGNC Symbol;Acc:HGNC:155] |
| CRISP3 | 233,02 | 644,24 | -1,47 | 6 | 49727384 | 49744437 | - | 2247 | cysteine rich secretory protein 3 [Source:HGNC Symbol;Acc:HGNC:16904] |
| GSDME | 56,91 | 13,50 | 2,07 | 7 | 24698351 | 24758113 | - | 8843 | gasdermin E [Source:HGNC Symbol;Acc:HGNC:2810] |
| HAS3 | 199,22 | 66,34 | 1,58 | 16 | 69105564 | 69118719 | + | 5289 | hyaluronan synthase 3 [Source:HGNC Symbol;Acc:HGNC:4820] |
| HS6ST2 | 501,63 | 179,90 | 1,48 | X | 132626016 | 132961395 | - | 5002 | heparan sulfate 6-O-sulfotransferase 2 [Source:HGNC Symbol;Acc:HGNC:19133] |
| FGF2 | 46,24 | 143,92 | -1,64 | 4 | 122826708 | 122898236 | + | 6775 | fibroblast growth factor 2 [Source:HGNC Symbol;Acc:HGNC:3676] |
| MDGA1 | 817,38 | 300,20 | 1,44 | 6 | 37630679 | 37699306 | - | 14187 | MAM domain containing glycosylphosphatidylinositol anchor 1 [Source:HGNC Symbol;Acc:HGNC:19267] |
| P3H2 | 578,12 | 210,26 | 1,46 | 3 | 189956728 | 190122437 | - | 5605 | prolyl 3-hydroxylase 2 [Source:HGNC Symbol;Acc:HGNC:19317] |
| ALDH3A1 | 2236,03 | 839,87 | 1,41 | 17 | 19737984 | 19748943 | - | 3918 | aldehyde dehydrogenase 3 family member A1 [Source:HGNC Symbol;Acc:HGNC:405] |
| LOXL1-AS1 | 85,38 | 24,75 | 1,78 | 15 | 73908071 | 73928248 | - | 4221 | LOXL1 antisense RNA 1 [Source:HGNC Symbol;Acc:HGNC:44169] |
| MIR221 | 24,01 | 2,26 | 3,35 | X | 45746157 | 45746266 | - | 110 | microRNA 221 [Source:HGNC Symbol;Acc:HGNC:31601] |
| SNORA3B | 23,12 | 2,26 | 3,29 | 11 | 8685439 | 8685569 | + | 131 | small nucleolar RNA, H/ACA box 3B [Source:HGNC Symbol;Acc:HGNC:32638] |
| WNT7A | 23,12 | 2,26 | 3,29 | 3 | 13816258 | 13880121 | - | 4579 | Wnt family member 7A [Source:HGNC Symbol;Acc:HGNC:12786] |
| AC037198.1 | 12,44 | 0,00 | 6,66 | 15 | 39586561 | 39587293 | + | 733 | novel transcript, sense intronic to THBS1 |
| AC008105.3 | 12,44 | 0,00 | 6,66 | 17 | 45190931 | 45222222 | - | 3719 | novel transcript, antisense to FMNL1 |
| LINC01293 | 12,44 | 0,00 | 6,66 | 2 | 74940258 | 74942670 | + | 1266 | long intergenic non-protein coding RNA 1293 [Source:HGNC Symbol;Acc:HGNC:50362] |
| LINC01615 | 12,44 | 0,00 | 6,66 | 6 | 169158092 | 169162924 | - | 1054 | long intergenic non-protein coding RNA 1615 [Source:HGNC Symbol;Acc:HGNC:51898] |
| ARL14 | 12,44 | 0,00 | 6,66 | 3 | 160677159 | 160678452 | + | 1294 | ADP ribosylation factor like GTPase 14 [Source:HGNC Symbol;Acc:HGNC:22974] |
| AC092153.1 | 12,44 | 0,00 | 6,66 | 2 | 160141981 | 160271888 | - | 2183 | novel transcript |
| FGA | 26,67 | 3,38 | 2,94 | 4 | 154583126 | 154590766 | - | 3956 | fibrinogen alpha chain [Source:HGNC Symbol;Acc:HGNC:3661] |
| AC012645.1 | 4,44 | 26,99 | -2,57 | 16 | 30096430 | 30104116 | + | 1650 | novel transcript, antisense to YPEL3 |
| LIPG | 2331,20 | 881,47 | 1,40 | 18 | 49560699 | 49599182 | + | 13112 | lipase G, endothelial type [Source:HGNC Symbol;Acc:HGNC:6623] |
| C4BPB | 16,89 | 1,13 | 3,77 | 1 | 207088842 | 207099993 | + | 3219 | complement component 4 binding protein beta [Source:HGNC Symbol;Acc:HGNC:1328] |
| KRT75 | 153,86 | 50,60 | 1,60 | 12 | 52424070 | 52434525 | - | 2324 | keratin 75 [Source:HGNC Symbol;Acc:HGNC:24431] |
| AQP1 | 590,58 | 217,00 | 1,44 | 7 | 30911694 | 30925516 | + | 3435 | aquaporin 1 (Colton blood group) [Source:HGNC Symbol;Acc:HGNC:633] |
| SLC2A6 | 69,37 | 201,26 | -1,53 | 9 | 133471095 | 133479137 | - | 3540 | solute carrier family 2 member 6 [Source:HGNC Symbol;Acc:HGNC:11011] |
| RNU5A-1 | 11,55 | 47,23 | -2,02 | 15 | 65296051 | 65296166 | + | 116 | RNA, U5A small nuclear 1 [Source:HGNC Symbol;Acc:HGNC:10211] |
| AL158209.1 | 15,11 | 58,47 | -1,94 | 10 | 21340233 | 21372950 | - | 439 | novel transcript |
| AL121956.1 | 81,82 | 23,62 | 1,79 | 6 | 166236969 | 166256947 | + | 1548 | uncharacterized LOC101929297 [Source:NCBI gene;Acc:101929297] |
| AC009229.1 | 102,28 | 31,49 | 1,70 | 2 | 38203363 | 38239590 | - | 622 | novel transcript |
| SBSPON | 123,62 | 39,36 | 1,65 | 8 | 73064540 | 73124088 | - | 4383 | somatomedin B and thrombospondin type 1 domain containing [Source:HGNC Symbol;Acc:HGNC:30362] |
| SNHG21 | 23,12 | 79,84 | -1,78 | 15 | 82750564 | 82757206 | + | 1053 | small nucleolar RNA host gene 21 [Source:HGNC Symbol;Acc:HGNC:50284] |
| IKBKGP1 | 180,55 | 61,85 | 1,54 | X | 154639978 | 154648275 | - | 1073 | inhibitor of nuclear factor kappa B kinase subunit gamma pseudogene 1 [Source:HGNC Symbol;Acc:HGNC:24455] |
| SLC47A2 | 91,60 | 26,99 | 1,76 | 17 | 19678288 | 19718979 | - | 6691 | solute carrier family 47 member 2 [Source:HGNC Symbol;Acc:HGNC:26439] |
| MME | 449,16 | 1182,78 | -1,40 | 3 | 155024124 | 155183729 | + | 9620 | membrane metalloendopeptidase [Source:HGNC Symbol;Acc:HGNC:7154] |
| SERPINB5 | 625,26 | 233,87 | 1,42 | 18 | 63476761 | 63505085 | + | 3939 | serpin family B member 5 [Source:HGNC Symbol;Acc:HGNC:8949] |
| IGFL2-AS1 | 416,25 | 1095,09 | -1,40 | 19 | 46189166 | 46203083 | - | 1751 | IGFL2 antisense RNA 1 [Source:HGNC Symbol;Acc:HGNC:52559] |
| CDC42EP5 | 1,77 | 20,25 | -3,42 | 19 | 54465026 | 54473264 | - | 903 | CDC42 effector protein 5 [Source:HGNC Symbol;Acc:HGNC:17408] |
| FP236383.2 | 2,66 | 20,25 | -2,87 | 21 | 8393419 | 8394341 | - | 923 | novel transcript, similar to YY1 associated myogenesis RNA 1 YAM1 |
| ZPLD1 | 186,77 | 64,10 | 1,54 | 3 | 102099244 | 102479841 | + | 5130 | zona pellucida like domain containing 1 [Source:HGNC Symbol;Acc:HGNC:27022] |
| LRRC26 | 11,55 | 46,11 | -1,98 | 9 | 137168758 | 137170051 | - | 1209 | leucine rich repeat containing 26 [Source:HGNC Symbol;Acc:HGNC:31409] |
| OLFM4 | 3896,61 | 1514,45 | 1,36 | 13 | 53028759 | 53052057 | + | 2897 | olfactomedin 4 [Source:HGNC Symbol;Acc:HGNC:17190] |
| RPL10P6 | 33,79 | 104,57 | -1,63 | 2 | 214847128 | 214847445 | + | 318 | ribosomal protein L10 pseudogene 6 [Source:HGNC Symbol;Acc:HGNC:52343] |
| AL451085.2 | 3,55 | 23,62 | -2,69 | 1 | 154961825 | 154962623 | + | 799 | uncharacterized LOC101928120 [Source:NCBI gene;Acc:101928120] |
| FLRT3 | 211,68 | 74,21 | 1,51 | 20 | 14322988 | 14337616 | - | 5190 | fibronectin leucine rich transmembrane protein 3 [Source:HGNC Symbol;Acc:HGNC:3762] |
| LAMC2 | 5460,23 | 2129,45 | 1,36 | 1 | 183186238 | 183244900 | + | 6543 | laminin subunit gamma 2 [Source:HGNC Symbol;Acc:HGNC:6493] |
| FGF7P6 | 87,16 | 26,99 | 1,69 | 9 | 62376161 | 62435199 | + | 2977 | fibroblast growth factor 7 pseudogene 6 [Source:HGNC Symbol;Acc:HGNC:27852] |
| IFI27 | 53,36 | 155,17 | -1,54 | 14 | 94104836 | 94116698 | + | 4147 | interferon alpha inducible protein 27 [Source:HGNC Symbol;Acc:HGNC:5397] |
| MMP24 | 105,83 | 33,74 | 1,65 | 20 | 35226654 | 35277000 | + | 4414 | matrix metallopeptidase 24 [Source:HGNC Symbol;Acc:HGNC:7172] |
| LINC01285 | 51,58 | 149,54 | -1,53 | X | 118839554 | 118919669 | + | 3861 | long intergenic non-protein coding RNA 1285 [Source:HGNC Symbol;Acc:HGNC:50344] |
| GBP4 | 35,57 | 109,07 | -1,61 | 1 | 89181148 | 89198932 | - | 6127 | guanylate binding protein 4 [Source:HGNC Symbol;Acc:HGNC:20480] |
| AC144831.1 | 0,88 | 16,87 | -4,07 | 17 | 83104255 | 83106910 | + | 2656 | novel transcript |
| PLA2G7 | 16,00 | 1,13 | 3,69 | 6 | 46704201 | 46735693 | - | 2026 | phospholipase A2 group VII [Source:HGNC Symbol;Acc:HGNC:9040] |
| CA8 | 91,60 | 28,12 | 1,70 | 8 | 60187347 | 60281412 | - | 5783 | carbonic anhydrase 8 [Source:HGNC Symbol;Acc:HGNC:1382] |
| LAMB3 | 5451,34 | 2159,81 | 1,34 | 1 | 209614870 | 209652466 | - | 4688 | laminin subunit beta 3 [Source:HGNC Symbol;Acc:HGNC:6490] |
| HRASLS5 | 0,00 | 12,38 | -6,65 | 11 | 63461404 | 63491194 | - | 3268 | HRAS like suppressor family member 5 [Source:HGNC Symbol;Acc:HGNC:24978] |
| AL355472.3 | 0,00 | 12,38 | -6,65 | 1 | 234357006 | 234365828 | + | 410 | novel transcript |
| PCDHGC4 | 11,55 | 0,00 | 6,56 | 5 | 141484997 | 141512979 | + | 4974 | protocadherin gamma subfamily C, 4 [Source:HGNC Symbol;Acc:HGNC:8717] |
| AL109955.1 | 11,55 | 0,00 | 6,56 | 20 | 57384160 | 57393062 | - | 1540 | uncharacterized LOC100291105 [Source:NCBI gene;Acc:100291105] |
| AC009948.2 | 11,55 | 0,00 | 6,56 | 2 | 178454716 | 178455428 | + | 713 | nuclear distribution gene C homolog (A. nidulans) (NUDC) pseudogene |
| PRPH | 11,55 | 0,00 | 6,56 | 12 | 49293252 | 49298686 | + | 4353 | peripherin [Source:HGNC Symbol;Acc:HGNC:9461] |
| ANKRD20A1 | 11,55 | 0,00 | 6,56 | 9 | 67859147 | 67902094 | + | 3887 | ankyrin repeat domain 20 family member A1 [Source:HGNC Symbol;Acc:HGNC:23665] |
| LINC01363 | 11,55 | 0,00 | 6,56 | 1 | 167175917 | 167195792 | - | 1375 | long intergenic non-protein coding RNA 1363 [Source:HGNC Symbol;Acc:HGNC:50598] |
| AC011473.4 | 315,74 | 116,94 | 1,43 | 19 | 50996018 | 51009581 | - | 1933 | novel transcript |
| FABP6 | 27,56 | 4,51 | 2,58 | 5 | 160187367 | 160238735 | + | 1164 | fatty acid binding protein 6 [Source:HGNC Symbol;Acc:HGNC:3561] |
| ALDH8A1 | 5,33 | 28,12 | -2,37 | 6 | 134917390 | 134950122 | - | 2868 | aldehyde dehydrogenase 8 family member A1 [Source:HGNC Symbol;Acc:HGNC:15471] |
| RGS17 | 2,66 | 19,12 | -2,78 | 6 | 153004459 | 153131249 | - | 7899 | regulator of G protein signaling 17 [Source:HGNC Symbol;Acc:HGNC:14088] |
| HOXA-AS3 | 1,77 | 19,12 | -3,34 | 7 | 27129977 | 27155928 | + | 5967 | HOXA cluster antisense RNA 3 [Source:HGNC Symbol;Acc:HGNC:43748] |
| LYPD1 | 38,24 | 9,00 | 2,07 | 2 | 132644853 | 132671579 | - | 2412 | LY6/PLAUR domain containing 1 [Source:HGNC Symbol;Acc:HGNC:28431] |
| WASIR2 | 8,88 | 39,36 | -2,13 | 16 | 22910 | 25123 | + | 1054 | WASH and IL9R antisense RNA 2 [Source:HGNC Symbol;Acc:HGNC:38609] |
| COL24A1 | 3,55 | 22,50 | -2,62 | 1 | 85729233 | 86156943 | - | 9568 | collagen type XXIV alpha 1 chain [Source:HGNC Symbol;Acc:HGNC:20821] |
| AC011444.1 | 14,22 | 52,85 | -1,88 | 19 | 5978403 | 6020363 | + | 639 | uncharacterized LOC100128568 [Source:NCBI gene;Acc:100128568] |
| PCSK9 | 176,99 | 456,48 | -1,37 | 1 | 55039548 | 55064852 | + | 4981 | proprotein convertase subtilisin/kexin type 9 [Source:HGNC Symbol;Acc:HGNC:20001] |
| AC089983.1 | 74,70 | 22,50 | 1,73 | 12 | 104262314 | 104280722 | - | 701 | novel transcript, antisense to TXNRD1 |
| LINC02577 | 79,15 | 24,75 | 1,67 | 7 | 106774955 | 106838480 | + | 4271 | long intergenic non-protein coding RNA 2577 [Source:HGNC Symbol;Acc:HGNC:53749] |
| AC106782.2 | 11,55 | 44,98 | -1,95 | 16 | 30355441 | 30357104 | + | 992 | uncharacterized LOC101928707 [Source:NCBI gene;Acc:101928707] |
| AC007608.3 | 11,55 | 44,98 | -1,95 | 16 | 50666211 | 50671639 | + | 5275 | uncharacterized LOC101927272 [Source:NCBI gene;Acc:101927272] |
| PTHLH | 35,57 | 7,88 | 2,16 | 12 | 27958084 | 27972705 | - | 3377 | parathyroid hormone like hormone [Source:HGNC Symbol;Acc:HGNC:9607] |
| C6orf223 | 39,13 | 115,81 | -1,56 | 6 | 44000580 | 44005958 | + | 3917 | chromosome 6 open reading frame 223 [Source:HGNC Symbol;Acc:HGNC:28692] |
| GALNT14 | 1564,51 | 628,50 | 1,32 | 2 | 30910467 | 31155202 | - | 4567 | polypeptide N-acetylgalactosaminyltransferase 14 [Source:HGNC Symbol;Acc:HGNC:22946] |
| AC067930.5 | 37,35 | 9,00 | 2,04 | 8 | 143573490 | 143577397 | + | 1173 | novel transcript |
| AC009229.2 | 56,03 | 15,75 | 1,82 | 2 | 38132637 | 38138946 | - | 351 | novel transcript |
| CAPN8 | 364,66 | 141,67 | 1,36 | 1 | 223538007 | 223665734 | - | 5192 | calpain 8 [Source:HGNC Symbol;Acc:HGNC:1485] |
| UPB1 | 18,67 | 61,85 | -1,72 | 22 | 24494107 | 24528390 | + | 6793 | beta-ureidopropionase 1 [Source:HGNC Symbol;Acc:HGNC:16297] |
| VPS37D | 22,23 | 71,97 | -1,69 | 7 | 73667825 | 73672112 | + | 1626 | VPS37D, ESCRT-I subunit [Source:HGNC Symbol;Acc:HGNC:18287] |
| KRT14 | 587,91 | 233,87 | 1,33 | 17 | 41582279 | 41586921 | - | 2281 | keratin 14 [Source:HGNC Symbol;Acc:HGNC:6416] |
| LINC00460 | 14,22 | 1,13 | 3,52 | 13 | 106374477 | 106384315 | + | 4232 | long intergenic non-protein coding RNA 460 [Source:HGNC Symbol;Acc:HGNC:42809] |
| AL162615.1 | 0,88 | 15,75 | -3,97 | 20 | 49829125 | 49831085 | + | 1653 | cytokine-like nuclear factor n-pac (N-PAC) pseudogene |
| AC004832.4 | 15,11 | 1,13 | 3,61 | 22 | 30421206 | 30421536 | - | 331 | novel transcript, sense intonic to |
| CASP17P | 0,88 | 15,75 | -3,97 | 11 | 104901549 | 104919073 | - | 4307 | caspase 17, pseudogene [Source:HGNC Symbol;Acc:HGNC:53657] |
| UGT1A1 | 87,16 | 28,12 | 1,63 | 2 | 233760248 | 233773299 | + | 4467 | UDP glucuronosyltransferase family 1 member A1 [Source:HGNC Symbol;Acc:HGNC:12530] |
| AC012617.1 | 49,80 | 137,18 | -1,46 | 19 | 36304580 | 36312668 | + | 2674 | uncharacterized LOC100134317 [Source:NCBI gene;Acc:100134317] |
| AL157935.1 | 13,33 | 48,36 | -1,85 | 9 | 127934503 | 127940952 | + | 2520 | novel transcript |
| GBP1 | 912,55 | 2226,14 | -1,29 | 1 | 89052319 | 89065360 | - | 4992 | guanylate binding protein 1 [Source:HGNC Symbol;Acc:HGNC:4182] |
| EGR2 | 34,68 | 7,88 | 2,12 | 10 | 62811996 | 62919900 | - | 3689 | early growth response 2 [Source:HGNC Symbol;Acc:HGNC:3239] |
| RETREG1 | 683,08 | 274,34 | 1,32 | 5 | 16473038 | 16617058 | - | 4568 | reticulophagy regulator 1 [Source:HGNC Symbol;Acc:HGNC:25964] |
| AC025259.3 | 99,61 | 33,74 | 1,56 | 12 | 52058459 | 52059503 | - | 915 | novel transcript |
| ITGAX | 24,01 | 3,38 | 2,79 | 16 | 31355134 | 31382997 | + | 7074 | integrin subunit alpha X [Source:HGNC Symbol;Acc:HGNC:6152] |
| SCD | 4216,80 | 10182,88 | -1,27 | 10 | 100347124 | 100364834 | + | 5362 | stearoyl-CoA desaturase [Source:HGNC Symbol;Acc:HGNC:10571] |
| AC008014.1 | 10,66 | 40,49 | -1,91 | 12 | 46383679 | 46876159 | + | 2227 | novel transcript |
| AL137784.1 | 2,66 | 18,00 | -2,70 | 6 | 99575712 | 99576456 | + | 745 | 40S ribososmal protein 3 (RPS3) pseudogene |
| GOLGA8Q | 2,66 | 18,00 | -2,70 | 15 | 30552078 | 30562501 | + | 2137 | golgin A8 family member Q [Source:HGNC Symbol;Acc:HGNC:44408] |
| EBAG9P1 | 2,66 | 18,00 | -2,70 | 10 | 99697407 | 99697949 | - | 543 | estrogen receptor binding site associated, antigen, 9 pseudogene 1 [Source:HGNC Symbol;Acc:HGNC:45233] |
| SPOCK1 | 20,45 | 2,26 | 3,12 | 5 | 136975298 | 137598379 | - | 6222 | SPARC (osteonectin), cwcv and kazal like domains proteoglycan 1 [Source:HGNC Symbol;Acc:HGNC:11251] |
| ATG9B | 20,45 | 2,26 | 3,12 | 7 | 151012209 | 151024499 | - | 6391 | autophagy related 9B [Source:HGNC Symbol;Acc:HGNC:21899] |
| AC009974.1 | 3,55 | 21,37 | -2,54 | 2 | 218818690 | 218819144 | + | 455 | novel transcript |
| AL365356.5 | 32,01 | 6,76 | 2,23 | 10 | 5594991 | 5596118 | - | 697 | uncharacterized LOC105376382 [Source:NCBI gene;Acc:105376382] |
| AC021945.1 | 54,25 | 15,75 | 1,78 | 8 | 119838736 | 119840385 | - | 1650 | TEC |
| EIF3C | 1410,63 | 579,03 | 1,28 | 16 | 28688558 | 28735730 | + | 6982 | eukaryotic translation initiation factor 3 subunit C [Source:HGNC Symbol;Acc:HGNC:3279] |
| S100A1 | 86,27 | 28,12 | 1,61 | 1 | 153627926 | 153632039 | + | 3030 | S100 calcium binding protein A1 [Source:HGNC Symbol;Acc:HGNC:10486] |
| TIMP4 | 10,66 | 0,00 | 6,44 | 3 | 12153051 | 12159351 | - | 1650 | TIMP metallopeptidase inhibitor 4 [Source:HGNC Symbol;Acc:HGNC:11823] |
| AC124947.1 | 10,66 | 0,00 | 6,44 | 12 | 93317135 | 93377736 | - | 865 | novel transcript, antisense to NUDT4 |
| MMP23A | 0,00 | 11,25 | -6,52 | 1 | 1699942 | 1701782 | + | 1017 | matrix metallopeptidase 23A (pseudogene) [Source:HGNC Symbol;Acc:HGNC:7170] |
| AC080162.1 | 10,66 | 0,00 | 6,44 | 2 | 9505445 | 9512412 | + | 491 | novel transcript |
| ERAS | 10,66 | 0,00 | 6,44 | X | 48826513 | 48830138 | + | 1303 | ES cell expressed Ras [Source:HGNC Symbol;Acc:HGNC:5174] |
| RN7SL600P | 10,66 | 0,00 | 6,44 | 1 | 150568971 | 150569269 | - | 299 | RNA, 7SL, cytoplasmic 600, pseudogene [Source:HGNC Symbol;Acc:HGNC:46616] |
| LINC01118 | 0,00 | 11,25 | -6,52 | 2 | 46698940 | 46822804 | + | 3360 | long intergenic non-protein coding RNA 1118 [Source:HGNC Symbol;Acc:HGNC:49261] |
| GABRD | 10,66 | 0,00 | 6,44 | 1 | 2019298 | 2030758 | + | 5556 | gamma-aminobutyric acid type A receptor delta subunit [Source:HGNC Symbol;Acc:HGNC:4084] |
| MYOZ3 | 10,66 | 0,00 | 6,44 | 5 | 150660874 | 150679365 | + | 6037 | myozenin 3 [Source:HGNC Symbol;Acc:HGNC:18565] |
| RAB3B | 504,30 | 203,51 | 1,31 | 1 | 51907956 | 51990764 | - | 12844 | RAB3B, member RAS oncogene family [Source:HGNC Symbol;Acc:HGNC:9778] |
| PNMA2 | 56,91 | 16,87 | 1,75 | 8 | 26504686 | 26514092 | - | 5579 | PNMA family member 2 [Source:HGNC Symbol;Acc:HGNC:9159] |
| GIPR | 33,79 | 96,70 | -1,51 | 19 | 45668244 | 45683724 | + | 4736 | gastric inhibitory polypeptide receptor [Source:HGNC Symbol;Acc:HGNC:4271] |
| CCDC168 | 28,45 | 5,63 | 2,31 | 13 | 102729369 | 102759070 | - | 21466 | coiled-coil domain containing 168 [Source:HGNC Symbol;Acc:HGNC:26851] |
| AL161431.1 | 570,12 | 231,62 | 1,30 | 13 | 109269634 | 109278512 | + | 5087 | novel transcript |
| AC132938.3 | 24,01 | 74,21 | -1,62 | 17 | 82454273 | 82458521 | - | 456 | novel transcript, antisense NARF |
| NFE2 | 150,31 | 55,10 | 1,45 | 12 | 54292111 | 54301121 | - | 2469 | nuclear factor, erythroid 2 [Source:HGNC Symbol;Acc:HGNC:7780] |
| ZSCAN1 | 41,79 | 115,81 | -1,47 | 19 | 58034032 | 58054631 | + | 2389 | zinc finger and SCAN domain containing 1 [Source:HGNC Symbol;Acc:HGNC:23712] |
| ARNTL2 | 1396,40 | 581,28 | 1,26 | 12 | 27332854 | 27425289 | + | 6983 | aryl hydrocarbon receptor nuclear translocator like 2 [Source:HGNC Symbol;Acc:HGNC:18984] |
| SERPINA3 | 99,61 | 34,86 | 1,51 | 14 | 94612377 | 94624055 | + | 5007 | serpin family A member 3 [Source:HGNC Symbol;Acc:HGNC:16] |
| C6orf15 | 53,36 | 15,75 | 1,75 | 6 | 31111223 | 31112559 | - | 1140 | chromosome 6 open reading frame 15 [Source:HGNC Symbol;Acc:HGNC:13927] |
| HMOX1 | 2661,18 | 1118,70 | 1,25 | 22 | 35380361 | 35394214 | + | 2405 | heme oxygenase 1 [Source:HGNC Symbol;Acc:HGNC:5013] |
| AL590666.3 | 25,78 | 4,51 | 2,49 | 1 | 156687695 | 156691997 | - | 4122 | novel transcript |
| AC004917.1 | 25,78 | 4,51 | 2,49 | 7 | 106372251 | 106770207 | - | 2082 | novel transcript, sense overlapping CCDC71L |
| NFATC1 | 10,66 | 39,36 | -1,87 | 18 | 79395856 | 79529325 | + | 9068 | nuclear factor of activated T cells 1 [Source:HGNC Symbol;Acc:HGNC:7775] |
| SPA17 | 14,22 | 49,48 | -1,79 | 11 | 124673798 | 124697518 | + | 5205 | sperm autoantigenic protein 17 [Source:HGNC Symbol;Acc:HGNC:11210] |
| FSTL4 | 65,81 | 20,25 | 1,70 | 5 | 133196455 | 133612564 | - | 6734 | follistatin like 4 [Source:HGNC Symbol;Acc:HGNC:21389] |
| PDK4 | 160,09 | 60,72 | 1,40 | 7 | 95583499 | 95596491 | - | 4519 | pyruvate dehydrogenase kinase 4 [Source:HGNC Symbol;Acc:HGNC:8812] |
| SLC35G2 | 22,23 | 3,38 | 2,68 | 3 | 136818647 | 136855892 | + | 4287 | solute carrier family 35 member G2 [Source:HGNC Symbol;Acc:HGNC:28480] |
| RBFOX3 | 23,12 | 3,38 | 2,73 | 17 | 79089345 | 79516148 | - | 9873 | RNA binding fox-1 homolog 3 [Source:HGNC Symbol;Acc:HGNC:27097] |
| LINC02515 | 74,70 | 191,14 | -1,35 | 4 | 187413564 | 187415697 | + | 536 | long intergenic non-protein coding RNA 2515 [Source:HGNC Symbol;Acc:HGNC:53504] |
| LUCAT1 | 312,18 | 125,93 | 1,31 | 5 | 91054834 | 91314547 | - | 16538 | lung cancer associated transcript 1 [Source:HGNC Symbol;Acc:HGNC:48498] |
| INA | 192,11 | 74,21 | 1,37 | 10 | 103277163 | 103290351 | + | 3231 | internexin neuronal intermediate filament protein alpha [Source:HGNC Symbol;Acc:HGNC:6057] |
| PTPN22 | 40,90 | 10,13 | 2,00 | 1 | 113813811 | 113871759 | - | 4503 | protein tyrosine phosphatase, non-receptor type 22 [Source:HGNC Symbol;Acc:HGNC:9652] |
| BMP6 | 40,90 | 10,13 | 2,00 | 6 | 7726797 | 7881422 | + | 2780 | bone morphogenetic protein 6 [Source:HGNC Symbol;Acc:HGNC:1073] |
| PLXDC2 | 184,10 | 447,49 | -1,28 | 10 | 19816239 | 20289856 | + | 12567 | plexin domain containing 2 [Source:HGNC Symbol;Acc:HGNC:21013] |
| AL592166.1 | 13,33 | 1,13 | 3,43 | 1 | 44759037 | 44775810 | - | 765 | novel transcript |
| CYP19A1 | 13,33 | 1,13 | 3,43 | 15 | 51208057 | 51338610 | - | 8437 | cytochrome P450 family 19 subfamily A member 1 [Source:HGNC Symbol;Acc:HGNC:2594] |
| VWA3B | 13,33 | 1,13 | 3,43 | 2 | 98087116 | 98313299 | + | 8307 | von Willebrand factor A domain containing 3B [Source:HGNC Symbol;Acc:HGNC:28385] |
| VIM | 76,48 | 25,87 | 1,56 | 10 | 17228259 | 17237593 | + | 5158 | vimentin [Source:HGNC Symbol;Acc:HGNC:12692] |
| AC010487.2 | 3,55 | 20,25 | -2,47 | 19 | 52923382 | 52924075 | + | 486 | novel transcript |
| AC083841.1 | 225,02 | 541,93 | -1,27 | 8 | 142763116 | 142766427 | + | 670 | novel transcript |
| INHBA-AS1 | 48,02 | 13,50 | 1,82 | 7 | 41693916 | 41779388 | + | 5417 | INHBA antisense RNA 1 [Source:HGNC Symbol;Acc:HGNC:40303] |
| AC131097.3 | 27,56 | 5,63 | 2,27 | 2 | 241808312 | 241812016 | - | 2250 | novel transcript |
| PPP1R2B | 2,66 | 16,87 | -2,60 | 5 | 156850538 | 156852528 | + | 1991 | PPP1R2 family member B [Source:HGNC Symbol;Acc:HGNC:16318] |
| MYO7B | 78,26 | 26,99 | 1,53 | 2 | 127535802 | 127637729 | + | 8800 | myosin VIIB [Source:HGNC Symbol;Acc:HGNC:7607] |
| AP3B2 | 132,52 | 49,48 | 1,42 | 15 | 82659281 | 82709914 | - | 9108 | adaptor related protein complex 3 subunit beta 2 [Source:HGNC Symbol;Acc:HGNC:567] |
| MSNP1 | 42,68 | 11,25 | 1,91 | 5 | 25909503 | 25911234 | + | 1732 | moesin pseudogene 1 [Source:HGNC Symbol;Acc:HGNC:7374] |
| IFI16 | 75,59 | 191,14 | -1,34 | 1 | 158999968 | 159055155 | + | 5294 | interferon gamma inducible protein 16 [Source:HGNC Symbol;Acc:HGNC:5395] |
| AC007292.1 | 32,90 | 7,88 | 2,05 | 19 | 4363789 | 4364640 | + | 746 | novel transcript, antisense to SH3GL1 |
| ADAM11 | 37,35 | 10,13 | 1,87 | 17 | 44759031 | 44781846 | + | 4770 | ADAM metallopeptidase domain 11 [Source:HGNC Symbol;Acc:HGNC:189] |
| SLC16A2 | 6054,38 | 2611,78 | 1,21 | X | 74421461 | 74533917 | + | 4324 | solute carrier family 16 member 2 [Source:HGNC Symbol;Acc:HGNC:10923] |
| CSF3R | 199,22 | 477,84 | -1,26 | 1 | 36466043 | 36483278 | - | 8429 | colony stimulating factor 3 receptor [Source:HGNC Symbol;Acc:HGNC:2439] |
| SERF1B | 56,91 | 18,00 | 1,66 | 5 | 70025247 | 70043113 | + | 3051 | small EDRK-rich factor 1B [Source:HGNC Symbol;Acc:HGNC:10756] |
| TLE3 | 6289,19 | 14450,76 | -1,20 | 15 | 70047790 | 70098176 | - | 12634 | transducin like enhancer of split 3 [Source:HGNC Symbol;Acc:HGNC:11839] |
| EGR1 | 26846,70 | 11693,95 | 1,20 | 5 | 138465490 | 138469315 | + | 3138 | early growth response 1 [Source:HGNC Symbol;Acc:HGNC:3238] |
| DNAJC12 | 16,00 | 52,85 | -1,71 | 10 | 67796665 | 67838166 | - | 2121 | DnaJ heat shock protein family (Hsp40) member C12 [Source:HGNC Symbol;Acc:HGNC:28908] |
| HSPA12A | 305,96 | 125,93 | 1,28 | 10 | 116671192 | 116850236 | - | 11834 | heat shock protein family A (Hsp70) member 12A [Source:HGNC Symbol;Acc:HGNC:19022] |
| MGP | 3710,71 | 8513,28 | -1,20 | 12 | 14881181 | 14885926 | - | 2639 | matrix Gla protein [Source:HGNC Symbol;Acc:HGNC:7060] |
| CBWD4P | 144,97 | 55,10 | 1,39 | 9 | 65287914 | 65323015 | - | 655 | COBW domain containing 4 pseudogene [Source:HGNC Symbol;Acc:HGNC:18520] |
| SCG5 | 29,34 | 6,76 | 2,10 | 15 | 32641676 | 32697098 | + | 2196 | secretogranin V [Source:HGNC Symbol;Acc:HGNC:10816] |
| CLEC1A | 7,11 | 30,37 | -2,07 | 12 | 10069554 | 10111627 | - | 3169 | C-type lectin domain family 1 member A [Source:HGNC Symbol;Acc:HGNC:24355] |
| LINC01444 | 9,77 | 0,00 | 6,32 | 18 | 14969001 | 14970468 | - | 1336 | long intergenic non-protein coding RNA 1444 [Source:HGNC Symbol;Acc:HGNC:50769] |
| AC002480.2 | 9,77 | 0,00 | 6,32 | 7 | 22589705 | 22591622 | + | 557 | novel transcript, antisense to STEAP1B |
| SNORA26 | 9,77 | 0,00 | 6,32 | 4 | 52713249 | 52713370 | + | 122 | small nucleolar RNA, H/ACA box 26 [Source:HGNC Symbol;Acc:HGNC:32616] |
| GNRHR | 9,77 | 0,00 | 6,32 | 4 | 67739328 | 67754360 | - | 2164 | gonadotropin releasing hormone receptor [Source:HGNC Symbol;Acc:HGNC:4421] |
| AL596223.1 | 9,77 | 0,00 | 6,32 | 10 | 69215333 | 69232490 | - | 1933 | uncharacterized LOC101928994 [Source:NCBI gene;Acc:101928994] |
| AC007256.1 | 9,77 | 0,00 | 6,32 | 2 | 201410544 | 201413308 | - | 2765 | SCY1-like 2 (S. cerevisiae) (SCYL2) pseudogene |
| AC020658.6 | 9,77 | 0,00 | 6,32 | 15 | 40258501 | 40260370 | + | 1870 | TEC |
| LAMB4 | 9,77 | 0,00 | 6,32 | 7 | 108023548 | 108130356 | - | 7016 | laminin subunit beta 4 [Source:HGNC Symbol;Acc:HGNC:6491] |
| MED28P3 | 9,77 | 0,00 | 6,32 | 2 | 186364598 | 186365128 | + | 531 | mediator complex subunit 28 pseudogene 3 [Source:HGNC Symbol;Acc:HGNC:45080] |
| IRS4 | 9,77 | 0,00 | 6,32 | X | 108732482 | 108736409 | - | 3928 | insulin receptor substrate 4 [Source:HGNC Symbol;Acc:HGNC:6128] |
| AC007314.1 | 0,00 | 10,13 | -6,37 | 2 | 10589166 | 10604830 | + | 565 | novel transcript |
| SLC4A10 | 9,77 | 0,00 | 6,32 | 2 | 161424332 | 161985282 | + | 7550 | solute carrier family 4 member 10 [Source:HGNC Symbol;Acc:HGNC:13811] |
| AC020658.3 | 9,77 | 0,00 | 6,32 | 15 | 40232082 | 40236109 | + | 4028 | novel transcript, sense intronic to PAK6 |
| AL137793.1 | 9,77 | 0,00 | 6,32 | 1 | 229092815 | 229094905 | - | 599 | novel transcript |
| NCKAP1L | 9,77 | 0,00 | 6,32 | 12 | 54497711 | 54548238 | + | 10596 | NCK associated protein 1 like [Source:HGNC Symbol;Acc:HGNC:4862] |
| CDH16 | 9,77 | 0,00 | 6,32 | 16 | 66908122 | 66918984 | - | 3656 | cadherin 16 [Source:HGNC Symbol;Acc:HGNC:1755] |
| MYRFL | 9,77 | 0,00 | 6,32 | 12 | 69825304 | 69959097 | + | 3524 | myelin regulatory factor like [Source:HGNC Symbol;Acc:HGNC:26316] |
| IGFL1P1 | 41,79 | 110,19 | -1,40 | 19 | 46196238 | 46197323 | + | 360 | IGF like family member 1 pseudogene 1 [Source:HGNC Symbol;Acc:HGNC:32956] |
| AC005332.5 | 32,90 | 89,96 | -1,45 | 17 | 68133201 | 68135935 | + | 2735 | novel transcript |
| AC006449.2 | 21,34 | 3,38 | 2,62 | 17 | 38703480 | 38706261 | - | 486 | novel transcript, antisense to MLLT6 |
| AC107214.1 | 21,34 | 3,38 | 2,62 | 4 | 183494938 | 183504494 | - | 559 | uncharacterized LOC389247 [Source:NCBI gene;Acc:389247] |
| LIN28B | 48,91 | 14,63 | 1,73 | 6 | 104936616 | 105083332 | + | 6002 | lin-28 homolog B [Source:HGNC Symbol;Acc:HGNC:32207] |
| IL11 | 76,48 | 26,99 | 1,50 | 19 | 55364389 | 55370463 | - | 2671 | interleukin 11 [Source:HGNC Symbol;Acc:HGNC:5966] |
| PKIB | 55,14 | 18,00 | 1,61 | 6 | 122471917 | 122726373 | + | 3432 | cAMP-dependent protein kinase inhibitor beta [Source:HGNC Symbol;Acc:HGNC:9018] |
| ADAMTSL4 | 244,59 | 100,07 | 1,29 | 1 | 150549369 | 150560937 | + | 6432 | ADAMTS like 4 [Source:HGNC Symbol;Acc:HGNC:19706] |
| CRABP1 | 71,15 | 24,75 | 1,52 | 15 | 78340324 | 78348230 | + | 993 | cellular retinoic acid binding protein 1 [Source:HGNC Symbol;Acc:HGNC:2338] |
| AL513550.1 | 64,92 | 21,37 | 1,60 | 6 | 99424922 | 99431373 | + | 1638 | thiosulfate sulfurtransferase like domain containing 3 [Source:NCBI gene;Acc:100130890] |
| CCL2 | 16,00 | 51,73 | -1,68 | 17 | 34255218 | 34257203 | + | 1986 | C-C motif chemokine ligand 2 [Source:HGNC Symbol;Acc:HGNC:10618] |
| IFIT1 | 104,94 | 251,86 | -1,26 | 10 | 89392546 | 89406486 | + | 4613 | interferon induced protein with tetratricopeptide repeats 1 [Source:HGNC Symbol;Acc:HGNC:5407] |
| AC090181.2 | 18,67 | 2,26 | 2,99 | 15 | 77067654 | 77068325 | - | 672 | novel transcript, sense intronic to TSPAN3 |
| C17orf107 | 3,55 | 19,12 | -2,39 | 17 | 4899418 | 4902932 | + | 3408 | chromosome 17 open reading frame 107 [Source:HGNC Symbol;Acc:HGNC:37238] |
| POTEE | 3,55 | 19,12 | -2,39 | 2 | 131218067 | 131265278 | + | 4192 | POTE ankyrin domain family member E [Source:HGNC Symbol;Acc:HGNC:33895] |
| AC115618.3 | 26,67 | 74,21 | -1,47 | X | 48580741 | 48581165 | + | 425 | TEC |
| METTL7A | 334,42 | 767,91 | -1,20 | 12 | 50923472 | 50932517 | + | 4083 | methyltransferase like 7A [Source:HGNC Symbol;Acc:HGNC:24550] |
| MGAT5B | 45,35 | 13,50 | 1,74 | 17 | 76868456 | 76950393 | + | 6386 | alpha-1,6-mannosylglycoprotein 6-beta-N-acetylglucosaminyltransferase B [Source:HGNC Symbol;Acc:HGNC:24140] |
| STAG3L1 | 167,20 | 391,27 | -1,23 | 7 | 75359194 | 75395383 | + | 2094 | stromal antigen 3-like 1 (pseudogene) [Source:HGNC Symbol;Acc:HGNC:33852] |
| PLAC4 | 8,88 | 34,86 | -1,96 | 21 | 41175231 | 41186788 | - | 10570 | placenta specific 4 [Source:HGNC Symbol;Acc:HGNC:14616] |
| AL670729.1 | 9,77 | 34,86 | -1,82 | 1 | 228407381 | 228409694 | + | 283 | novel transcript |
| CLIP4 | 1011,28 | 444,11 | 1,19 | 2 | 29097705 | 29189643 | + | 7775 | CAP-Gly domain containing linker protein family member 4 [Source:HGNC Symbol;Acc:HGNC:26108] |
| NAV3 | 16,00 | 2,26 | 2,77 | 12 | 77324641 | 78213008 | + | 13265 | neuron navigator 3 [Source:HGNC Symbol;Acc:HGNC:15998] |
| CYP24A1 | 1,77 | 16,87 | -3,16 | 20 | 54153449 | 54173973 | - | 3972 | cytochrome P450 family 24 subfamily A member 1 [Source:HGNC Symbol;Acc:HGNC:2602] |
| OACYLP | 1,77 | 16,87 | -3,16 | 18 | 58996734 | 59069338 | + | 3166 | O-acyltransferase like, pseudogene [Source:HGNC Symbol;Acc:HGNC:44362] |
| GPR1 | 12,44 | 1,13 | 3,33 | 2 | 206175316 | 206218047 | - | 2834 | G protein-coupled receptor 1 [Source:HGNC Symbol;Acc:HGNC:4463] |
| C7orf31 | 0,88 | 13,50 | -3,75 | 7 | 25134697 | 25180356 | - | 3837 | chromosome 7 open reading frame 31 [Source:HGNC Symbol;Acc:HGNC:21722] |
| AC010809.1 | 12,44 | 1,13 | 3,33 | 15 | 33858602 | 33864825 | - | 562 | novel transcript, antisense to RYR3 |
| AC063965.1 | 12,44 | 1,13 | 3,33 | 10 | 87878692 | 87880427 | + | 499 | novel transcript, sense intronic to PTEN |
| KIRREL3-AS1 | 12,44 | 1,13 | 3,33 | 11 | 126543947 | 126610948 | + | 586 | KIRREL3 antisense RNA 1 [Source:HGNC Symbol;Acc:HGNC:42655] |
| RF00015 | 0,88 | 13,50 | -3,75 | 1 | 150608507 | 150608623 | - | 117 |  |
| FLT1 | 12,44 | 1,13 | 3,33 | 13 | 28300344 | 28495145 | - | 12567 | fms related tyrosine kinase 1 [Source:HGNC Symbol;Acc:HGNC:3763] |
| CDK15 | 24,01 | 4,51 | 2,38 | 2 | 201790461 | 201895550 | + | 6141 | cyclin dependent kinase 15 [Source:HGNC Symbol;Acc:HGNC:14434] |
| AL359265.3 | 5,33 | 24,75 | -2,19 | 1 | 176207648 | 176229330 | + | 579 | novel transcript |
| LINC01979 | 5,33 | 24,75 | -2,19 | 17 | 79915252 | 79926725 | - | 5271 | long intergenic non-protein coding RNA 1979 [Source:HGNC Symbol;Acc:HGNC:52807] |
| GSTP1 | 725,77 | 1635,88 | -1,17 | 11 | 67583595 | 67586656 | + | 1911 | glutathione S-transferase pi 1 [Source:HGNC Symbol;Acc:HGNC:4638] |
| MALAT1 | 15210,22 | 6809,95 | 1,16 | 11 | 65497688 | 65506516 | + | 8829 | metastasis associated lung adenocarcinoma transcript 1 [Source:HGNC Symbol;Acc:HGNC:29665] |
| OGDHL | 70,26 | 24,75 | 1,50 | 10 | 49734643 | 49762379 | - | 5724 | oxoglutarate dehydrogenase like [Source:HGNC Symbol;Acc:HGNC:25590] |
| SRPX2 | 1029,07 | 455,36 | 1,18 | X | 100644166 | 100675788 | + | 7548 | sushi repeat containing protein X-linked 2 [Source:HGNC Symbol;Acc:HGNC:30668] |
| BARX2 | 909,88 | 2033,89 | -1,16 | 11 | 129375940 | 129452279 | + | 3660 | BARX homeobox 2 [Source:HGNC Symbol;Acc:HGNC:956] |
| AC068768.1 | 13,33 | 43,86 | -1,71 | 12 | 123252030 | 123261483 | - | 1756 | novel transcript |
| EFEMP1 | 215,23 | 493,58 | -1,20 | 2 | 55865967 | 55924139 | - | 3962 | EGF containing fibulin extracellular matrix protein 1 [Source:HGNC Symbol;Acc:HGNC:3218] |
| RTP4 | 48,91 | 122,56 | -1,32 | 3 | 187368332 | 187372076 | + | 1554 | receptor transporter protein 4 [Source:HGNC Symbol;Acc:HGNC:23992] |
| RELN | 261,48 | 110,19 | 1,25 | 7 | 103471784 | 103989516 | - | 12642 | reelin [Source:HGNC Symbol;Acc:HGNC:9957] |
| DHRS2 | 112,95 | 43,86 | 1,36 | 14 | 23630115 | 23645639 | + | 7556 | dehydrogenase/reductase 2 [Source:HGNC Symbol;Acc:HGNC:18349] |
| B3GALT5 | 58,69 | 19,12 | 1,61 | 21 | 39556442 | 39673137 | + | 14319 | beta-1,3-galactosyltransferase 5 [Source:HGNC Symbol;Acc:HGNC:920] |
| ECM1 | 303,29 | 129,31 | 1,23 | 1 | 150508062 | 150513789 | + | 3360 | extracellular matrix protein 1 [Source:HGNC Symbol;Acc:HGNC:3153] |
| SLC25A18 | 103,17 | 39,36 | 1,39 | 22 | 17563439 | 17590994 | + | 4741 | solute carrier family 25 member 18 [Source:HGNC Symbol;Acc:HGNC:10988] |
| AC015813.1 | 31,12 | 7,88 | 1,97 | 17 | 57989039 | 57994850 | - | 5812 | novel transcript |
| FOXD3-AS1 | 30,23 | 7,88 | 1,93 | 1 | 63320884 | 63324441 | - | 1208 | FOXD3 antisense RNA 1 [Source:HGNC Symbol;Acc:HGNC:40241] |
| EEF1A1P19 | 31,12 | 7,88 | 1,97 | 5 | 43495073 | 43496454 | - | 1382 | eukaryotic translation elongation factor 1 alpha 1 pseudogene 19 [Source:HGNC Symbol;Acc:HGNC:37892] |
| GBAP1 | 561,22 | 246,23 | 1,19 | 1 | 155213821 | 155227422 | - | 3656 | glucosylceramidase beta pseudogene 1 [Source:HGNC Symbol;Acc:HGNC:4178] |
| LINC01508 | 54,25 | 136,05 | -1,32 | 9 | 90300902 | 90433505 | - | 1597 | long intergenic non-protein coding RNA 1508 [Source:HGNC Symbol;Acc:HGNC:51190] |
| DACH1 | 17,78 | 52,85 | -1,56 | 13 | 71437966 | 71867192 | - | 5389 | dachshund family transcription factor 1 [Source:HGNC Symbol;Acc:HGNC:2663] |
| STRIP2 | 2300,96 | 1035,50 | 1,15 | 7 | 129434433 | 129488399 | + | 5876 | striatin interacting protein 2 [Source:HGNC Symbol;Acc:HGNC:22209] |
| AC011330.1 | 78,26 | 28,12 | 1,47 | 15 | 43663654 | 43684339 | - | 3235 | histidine acid phosphatase domain containing 2A (HISPPD2A) pseudogene |
| TMPRSS6 | 4,44 | 21,37 | -2,23 | 22 | 37065436 | 37109713 | - | 4232 | transmembrane serine protease 6 [Source:HGNC Symbol;Acc:HGNC:16517] |
| LINC01698 | 20,45 | 3,38 | 2,56 | 1 | 209367662 | 209379690 | + | 892 | long intergenic non-protein coding RNA 1698 [Source:HGNC Symbol;Acc:HGNC:52486] |
| AC011483.3 | 25,78 | 5,63 | 2,17 | 19 | 50962197 | 50962781 | - | 585 | TEC |
| LINC00886 | 53,36 | 18,00 | 1,56 | 3 | 156747346 | 156817062 | - | 3822 | long intergenic non-protein coding RNA 886 [Source:HGNC Symbol;Acc:HGNC:48572] |
| OAS2 | 111,17 | 260,85 | -1,23 | 12 | 112978395 | 113011723 | + | 6264 | 2'-5'-oligoadenylate synthetase 2 [Source:HGNC Symbol;Acc:HGNC:8087] |
| ADGRG2 | 120,06 | 47,23 | 1,34 | X | 18989309 | 19122637 | - | 10155 | adhesion G protein-coupled receptor G2 [Source:HGNC Symbol;Acc:HGNC:4516] |
| AC010761.1 | 71,15 | 25,87 | 1,46 | 17 | 28721487 | 28722877 | - | 899 | novel transcript, antisense to RPL23A |
| AC008982.2 | 82,71 | 30,37 | 1,44 | 19 | 38844729 | 38845499 | - | 493 | novel transcript, sense intronic to HNRNPL |
| AL512599.1 | 231,24 | 522,81 | -1,18 | 1 | 40132764 | 40133448 | + | 685 | ornithine decarboxylase antizyme 1 (OAZ1) pseudogene |
| AC092164.1 | 9,77 | 33,74 | -1,77 | 2 | 28708953 | 28736205 | - | 1438 | novel transcript |
| AC110749.1 | 32,90 | 9,00 | 1,86 | 17 | 38855314 | 38855670 | + | 357 | ribosomal protein S20 pseudogene 35 |
| AL078582.1 | 32,90 | 9,00 | 1,86 | 6 | 152112759 | 152118397 | + | 511 | novel transcript, sense intronic to ESR1 |
| MUC16 | 238,36 | 101,20 | 1,24 | 19 | 8848844 | 8981342 | - | 43830 | mucin 16, cell surface associated [Source:HGNC Symbol;Acc:HGNC:15582] |
| BTNL10 | 289,95 | 648,74 | -1,16 | 1 | 228510425 | 228512305 | - | 731 | butyrophilin like 10 [Source:HGNC Symbol;Acc:HGNC:42540] |
| TRAPPC12-AS1 | 48,91 | 15,75 | 1,63 | 2 | 3481242 | 3482409 | - | 908 | TRAPPC12 antisense RNA 1 [Source:HGNC Symbol;Acc:HGNC:41046] |
| RCAN2 | 1326,14 | 600,39 | 1,14 | 6 | 46220736 | 46491972 | - | 3712 | regulator of calcineurin 2 [Source:HGNC Symbol;Acc:HGNC:3041] |
| MYADM | 1190,05 | 2609,53 | -1,13 | 19 | 53866223 | 53876437 | + | 3675 | myeloid associated differentiation marker [Source:HGNC Symbol;Acc:HGNC:7544] |
| AP5B1 | 209,01 | 471,10 | -1,17 | 11 | 65775893 | 65780802 | - | 4811 | adaptor related protein complex 5 subunit beta 1 [Source:HGNC Symbol;Acc:HGNC:25104] |
| DKK1 | 668,85 | 299,08 | 1,16 | 10 | 52314296 | 52318042 | + | 2206 | dickkopf WNT signaling pathway inhibitor 1 [Source:HGNC Symbol;Acc:HGNC:2891] |
| KCNN3 | 27,56 | 6,76 | 2,01 | 1 | 154697455 | 154870280 | - | 13598 | potassium calcium-activated channel subfamily N member 3 [Source:HGNC Symbol;Acc:HGNC:6292] |
| CLMP | 79,15 | 29,24 | 1,43 | 11 | 123069865 | 123195281 | - | 5097 | CXADR like membrane protein [Source:HGNC Symbol;Acc:HGNC:24039] |
| ADCYAP1 | 79,15 | 29,24 | 1,43 | 18 | 904943 | 912172 | + | 3805 | adenylate cyclase activating polypeptide 1 [Source:HGNC Symbol;Acc:HGNC:241] |
| DDN-AS1 | 17,78 | 2,26 | 2,92 | 12 | 48998367 | 49019235 | + | 2238 | DDN and PRKAG1 antisense RNA 1 [Source:HGNC Symbol;Acc:HGNC:53464] |
| AL355482.1 | 17,78 | 2,26 | 2,92 | 1 | 182062677 | 182069253 | - | 2463 | novel transcript (LOC149157) |
| ISCA1P4 | 3,55 | 18,00 | -2,30 | 15 | 40948736 | 40948869 | - | 134 | iron-sulfur cluster assembly 1 pseudogene 4 [Source:HGNC Symbol;Acc:HGNC:38025] |
| CNNM1 | 46,24 | 115,81 | -1,32 | 10 | 99329099 | 99394330 | + | 6413 | cyclin and CBS domain divalent metal cation transport mediator 1 [Source:HGNC Symbol;Acc:HGNC:102] |
| AC004067.1 | 22,23 | 4,51 | 2,27 | 4 | 109692004 | 109692703 | + | 700 | novel transcript, antisense to CASP6 |
| AC011979.1 | 5,33 | 23,62 | -2,12 | 11 | 9660438 | 9660920 | + | 483 | ribosomal protein L21 (RPL21) pseudogene |
| AC026979.2 | 5,33 | 23,62 | -2,12 | 8 | 30155830 | 30156232 | - | 403 | novel transcript |
| CAPNS2 | 22,23 | 4,51 | 2,27 | 16 | 55566672 | 55567687 | + | 1016 | calpain small subunit 2 [Source:HGNC Symbol;Acc:HGNC:16371] |
| DNM3 | 303,29 | 132,68 | 1,19 | 1 | 171841498 | 172418466 | + | 11329 | dynamin 3 [Source:HGNC Symbol;Acc:HGNC:29125] |
| PRSS23 | 1031,74 | 468,85 | 1,14 | 11 | 86791059 | 86952910 | + | 11725 | serine protease 23 [Source:HGNC Symbol;Acc:HGNC:14370] |
| EPHX4 | 129,85 | 52,85 | 1,30 | 1 | 92029982 | 92063536 | + | 2251 | epoxide hydrolase 4 [Source:HGNC Symbol;Acc:HGNC:23758] |
| CTAGE7P | 8,88 | 0,00 | 6,18 | 10 | 130106046 | 130108481 | + | 2436 | CTAGE family member 7, pseudogene [Source:HGNC Symbol;Acc:HGNC:25111] |
| C16orf86 | 0,00 | 9,00 | -6,20 | 16 | 67666816 | 67668758 | + | 1789 | chromosome 16 open reading frame 86 [Source:HGNC Symbol;Acc:HGNC:33755] |
| TDO2 | 8,88 | 0,00 | 6,18 | 4 | 155854738 | 155920406 | + | 5264 | tryptophan 2,3-dioxygenase [Source:HGNC Symbol;Acc:HGNC:11708] |
| AC010530.1 | 8,88 | 0,00 | 6,18 | 16 | 67738588 | 67739922 | - | 569 | novel transcript |
| PRDX3P2 | 8,88 | 0,00 | 6,18 | 1 | 28526318 | 28527227 | - | 611 | peroxiredoxin 3 pseudogene 2 [Source:HGNC Symbol;Acc:HGNC:39265] |
| AC091152.2 | 0,00 | 9,00 | -6,20 | 17 | 44673689 | 44676257 | - | 2569 | novel transcript, antisense to C17orf104 |
| Z97055.2 | 8,88 | 0,00 | 6,18 | 22 | 43812456 | 43817394 | + | 1981 | novel transcript, antisense to EFCAB6 |
| AC022336.3 | 0,00 | 9,00 | -6,20 | 3 | 124791119 | 124793104 | - | 1986 | TEC |
| ACTG1P17 | 8,88 | 0,00 | 6,18 | 15 | 82725873 | 82738904 | - | 2096 | actin gamma 1 pseudogene 17 [Source:HGNC Symbol;Acc:HGNC:51497] |
| SERPINC1 | 0,00 | 9,00 | -6,20 | 1 | 173903804 | 173917378 | - | 1768 | serpin family C member 1 [Source:HGNC Symbol;Acc:HGNC:775] |
| AC016924.1 | 8,88 | 0,00 | 6,18 | 3 | 126266796 | 126291279 | + | 795 | novel transcript |
| AC084026.3 | 8,88 | 0,00 | 6,18 | 8 | 29587001 | 29588426 | + | 1426 | TEC |
| KCNV1 | 8,88 | 0,00 | 6,18 | 8 | 109963645 | 109975847 | - | 6979 | potassium voltage-gated channel modifier subfamily V member 1 [Source:HGNC Symbol;Acc:HGNC:18861] |
| AC131902.1 | 0,00 | 9,00 | -6,20 | 8 | 53395211 | 53395946 | - | 594 | novel transcript |
| AC021066.1 | 0,00 | 9,00 | -6,20 | 12 | 52274647 | 52279156 | - | 647 | novel transcript, antisense to KRT86 |
| AL109840.2 | 0,00 | 9,00 | -6,20 | 20 | 58916000 | 58981169 | - | 1925 | novel transcript |
| LINC00163 | 0,00 | 9,00 | -6,20 | 21 | 44989864 | 44994086 | - | 2155 | long intergenic non-protein coding RNA 163 [Source:HGNC Symbol;Acc:HGNC:33165] |
| SCTR | 8,88 | 0,00 | 6,18 | 2 | 119439843 | 119525301 | - | 3426 | secretin receptor [Source:HGNC Symbol;Acc:HGNC:10608] |
| CYP1A2 | 8,88 | 0,00 | 6,18 | 15 | 74748844 | 74756202 | + | 2728 | cytochrome P450 family 1 subfamily A member 2 [Source:HGNC Symbol;Acc:HGNC:2596] |
| NUDT19P3 | 0,00 | 9,00 | -6,20 | 6 | 114019621 | 114020483 | - | 555 | nudix hydrolase 19 pseudogene 3 [Source:HGNC Symbol;Acc:HGNC:43587] |
| UQCRFS1P2 | 0,00 | 9,00 | -6,20 | 7 | 138701607 | 138703062 | + | 722 | ubiquinol-cytochrome c reductase, Rieske iron-sulfur polypeptide 1 pseudogene 2 [Source:HGNC Symbol;Acc:HGNC:39172] |
| MAP1B | 400,24 | 177,65 | 1,17 | 5 | 72107234 | 72209570 | + | 12630 | microtubule associated protein 1B [Source:HGNC Symbol;Acc:HGNC:6836] |
| C2CD4A | 248,14 | 107,94 | 1,20 | 15 | 62066977 | 62070917 | + | 3445 | C2 calcium dependent domain containing 4A [Source:HGNC Symbol;Acc:HGNC:33627] |
| PIP | 85,38 | 32,62 | 1,39 | 7 | 143132077 | 143139746 | + | 585 | prolactin induced protein [Source:HGNC Symbol;Acc:HGNC:8993] |
| ITGAM | 174,32 | 73,09 | 1,25 | 16 | 31259967 | 31332892 | + | 5688 | integrin subunit alpha M [Source:HGNC Symbol;Acc:HGNC:6149] |
| IFFO2 | 619,93 | 1351,43 | -1,12 | 1 | 18904281 | 18956686 | - | 6188 | intermediate filament family orphan 2 [Source:HGNC Symbol;Acc:HGNC:27006] |
| SYNGR3 | 8,00 | 30,37 | -1,91 | 16 | 1989660 | 1994275 | + | 3420 | synaptogyrin 3 [Source:HGNC Symbol;Acc:HGNC:11501] |
| AC040169.1 | 29,34 | 77,59 | -1,40 | 16 | 84117051 | 84117571 | + | 521 | novel transcript, antisense to MBTPS1 |
| LINC00552 | 89,82 | 34,86 | 1,36 | 13 | 113748511 | 113751089 | - | 2579 | long intergenic non-protein coding RNA 552 [Source:HGNC Symbol;Acc:HGNC:43692] |
| XK | 128,96 | 52,85 | 1,29 | X | 37685756 | 37732130 | + | 5209 | X-linked Kx blood group [Source:HGNC Symbol;Acc:HGNC:12811] |
| KNDC1 | 1,77 | 15,75 | -3,06 | 10 | 133160447 | 133226412 | + | 7985 | kinase non-catalytic C-lobe domain containing 1 [Source:HGNC Symbol;Acc:HGNC:29374] |
| CCDC74B | 2,66 | 15,75 | -2,51 | 2 | 130139287 | 130145134 | - | 4487 | coiled-coil domain containing 74B [Source:HGNC Symbol;Acc:HGNC:25267] |
| FAM217A | 15,11 | 2,26 | 2,68 | 6 | 4049434 | 4087344 | - | 3138 | family with sequence similarity 217 member A [Source:HGNC Symbol;Acc:HGNC:21362] |
| AC022165.1 | 2,66 | 15,75 | -2,51 | 16 | 84495599 | 84497495 | + | 596 | novel transcript, antisense to KIAA1609 |
| SLX1B | 15,11 | 2,26 | 2,68 | 16 | 29454501 | 29458219 | + | 2462 | SLX1 homolog B, structure-specific endonuclease subunit [Source:HGNC Symbol;Acc:HGNC:28748] |
| AC090229.1 | 1,77 | 15,75 | -3,06 | 18 | 35716370 | 35717978 | + | 1609 | novel transcript |
| AC015910.1 | 80,93 | 30,37 | 1,41 | 17 | 39238466 | 39250818 | + | 1294 | novel pseudogene |
| EGR3 | 119,18 | 48,36 | 1,30 | 8 | 22687659 | 22693302 | - | 4855 | early growth response 3 [Source:HGNC Symbol;Acc:HGNC:3240] |
| TCHHL1 | 235,69 | 102,32 | 1,20 | 1 | 152084144 | 152089064 | - | 3603 | trichohyalin like 1 [Source:HGNC Symbol;Acc:HGNC:31796] |
| XDH | 1811,77 | 838,75 | 1,11 | 2 | 31334321 | 31414715 | - | 6037 | xanthine dehydrogenase [Source:HGNC Symbol;Acc:HGNC:12805] |
| TRPC1 | 24,89 | 5,63 | 2,12 | 3 | 142724074 | 142807888 | + | 4768 | transient receptor potential cation channel subfamily C member 1 [Source:HGNC Symbol;Acc:HGNC:12333] |
| TACR1 | 24,89 | 5,63 | 2,12 | 2 | 75046463 | 75199700 | - | 5308 | tachykinin receptor 1 [Source:HGNC Symbol;Acc:HGNC:11526] |
| AC010280.2 | 6,22 | 25,87 | -2,03 | 5 | 68430427 | 68434481 | - | 410 | novel transcript |
| IL32 | 32,01 | 9,00 | 1,82 | 16 | 3065297 | 3082192 | + | 3209 | interleukin 32 [Source:HGNC Symbol;Acc:HGNC:16830] |
| CLDN16 | 8,88 | 32,62 | -1,86 | 3 | 190322541 | 190412143 | + | 3583 | claudin 16 [Source:HGNC Symbol;Acc:HGNC:2037] |
| HBEGF | 271,27 | 119,19 | 1,19 | 5 | 140332843 | 140346631 | - | 3018 | heparin binding EGF like growth factor [Source:HGNC Symbol;Acc:HGNC:3059] |
| AC005912.1 | 768,46 | 1658,37 | -1,11 | 12 | 3211663 | 3211917 | - | 255 | ribosomal protein S27 (RPS27) pseudogene |
| AC007785.1 | 16,89 | 50,60 | -1,57 | 19 | 46101122 | 46196218 | + | 332 | novel transcript |
| AL391421.1 | 16,89 | 50,60 | -1,57 | 10 | 77866875 | 77869610 | + | 1922 | novel transcript, antisense to DLG5 |
| DRAM1 | 623,48 | 1348,06 | -1,11 | 12 | 101877351 | 102012130 | + | 4180 | DNA damage regulated autophagy modulator 1 [Source:HGNC Symbol;Acc:HGNC:25645] |
| MT-ND1 | 48698,34 | 22786,38 | 1,10 | MT | 3307 | 4262 | + | 956 | mitochondrially encoded NADH:ubiquinone oxidoreductase core subunit 1 [Source:HGNC Symbol;Acc:HGNC:7455] |
| LAPTM5 | 11,55 | 1,13 | 3,23 | 1 | 30732469 | 30757820 | - | 3274 | lysosomal protein transmembrane 5 [Source:HGNC Symbol;Acc:HGNC:29612] |
| NPY4R2 | 11,55 | 1,13 | 3,23 | 10 | 47918739 | 47923524 | + | 1956 | neuropeptide Y receptor Y4-2 [Source:HGNC Symbol;Acc:HGNC:52383] |
| AC078817.1 | 0,88 | 12,38 | -3,62 | 12 | 80102899 | 80103333 | - | 435 | ribosomal protein L26 (RPL26) pseudogene |
| AL359921.1 | 11,55 | 1,13 | 3,23 | 1 | 236540094 | 236550280 | - | 494 | novel transcript |
| AL023806.3 | 11,55 | 1,13 | 3,23 | 6 | 145789270 | 145791973 | - | 2704 | TEC |
| AC000120.1 | 11,55 | 1,13 | 3,23 | 7 | 92200014 | 92206857 | - | 1589 | novel transcript |
| CYCSP10 | 11,55 | 1,13 | 3,23 | 3 | 10000647 | 10000940 | - | 294 | cytochrome c, somatic pseudogene 10 [Source:HGNC Symbol;Acc:HGNC:24384] |
| ALOX15B | 11,55 | 1,13 | 3,23 | 17 | 8039017 | 8049134 | + | 3028 | arachidonate 15-lipoxygenase, type B [Source:HGNC Symbol;Acc:HGNC:434] |
| AC093249.2 | 0,88 | 12,38 | -3,62 | 16 | 30585907 | 30608593 | + | 482 | novel transcript, antisense to ZNF689 |
| KCTD16 | 11,55 | 1,13 | 3,23 | 5 | 144170832 | 144485686 | + | 14294 | potassium channel tetramerization domain containing 16 [Source:HGNC Symbol;Acc:HGNC:29244] |
| NPTX2 | 11,55 | 1,13 | 3,23 | 7 | 98617297 | 98629868 | + | 3016 | neuronal pentraxin 2 [Source:HGNC Symbol;Acc:HGNC:7953] |
| DTX1 | 0,88 | 12,38 | -3,62 | 12 | 113056709 | 113098028 | + | 4387 | deltex E3 ubiquitin ligase 1 [Source:HGNC Symbol;Acc:HGNC:3060] |
| FAM25C | 11,55 | 1,13 | 3,23 | 10 | 47995355 | 47999791 | - | 319 | family with sequence similarity 25 member C [Source:HGNC Symbol;Acc:HGNC:23586] |
| AC027449.1 | 0,88 | 12,38 | -3,62 | 18 | 22347846 | 22348252 | - | 407 | novel transcript |
| IGFBP1 | 19,56 | 3,38 | 2,49 | 7 | 45888357 | 45893668 | + | 1653 | insulin like growth factor binding protein 1 [Source:HGNC Symbol;Acc:HGNC:5469] |
| AC135977.1 | 4,44 | 20,25 | -2,16 | 11 | 49433480 | 49434438 | - | 959 | solute carrier family 25, member 33 (SLC25A33) pseudogene |
| AC069222.1 | 19,56 | 3,38 | 2,49 | 3 | 99802699 | 99806058 | - | 3360 | novel transcript |
| ADD2 | 19,56 | 3,38 | 2,49 | 2 | 70607618 | 70768225 | - | 12673 | adducin 2 [Source:HGNC Symbol;Acc:HGNC:244] |
| AC010976.1 | 62,25 | 22,50 | 1,46 | 2 | 127455394 | 127514623 | + | 5510 | novel transcript, antisense to IWS1 |
| AL356234.2 | 146,75 | 61,85 | 1,25 | 6 | 137657998 | 137763984 | + | 2312 | novel transcript |
| ASTL | 18,67 | 52,85 | -1,49 | 2 | 96123850 | 96138436 | - | 1654 | astacin like metalloendopeptidase [Source:HGNC Symbol;Acc:HGNC:31704] |
| RPL36A | 442,04 | 200,14 | 1,14 | X | 101390824 | 101396154 | + | 3964 | ribosomal protein L36a [Source:HGNC Symbol;Acc:HGNC:10359] |
| DLSTP1 | 42,68 | 13,50 | 1,65 | 1 | 75743423 | 75744776 | - | 1354 | dihydrolipoamide S-succinyltransferase pseudogene 1 [Source:HGNC Symbol;Acc:HGNC:2912] |
| EDARADD | 14,22 | 43,86 | -1,62 | 1 | 236348257 | 236502915 | + | 3985 | EDAR associated death domain [Source:HGNC Symbol;Acc:HGNC:14341] |
| MIR200B | 33,79 | 10,13 | 1,73 | 1 | 1167104 | 1167198 | + | 95 | microRNA 200b [Source:HGNC Symbol;Acc:HGNC:31579] |
| SYNE1 | 79,15 | 30,37 | 1,38 | 6 | 152121684 | 152637801 | - | 46064 | spectrin repeat containing nuclear envelope protein 1 [Source:HGNC Symbol;Acc:HGNC:17089] |
| PCDH8 | 66,70 | 24,75 | 1,43 | 13 | 52842889 | 52848641 | - | 5150 | protocadherin 8 [Source:HGNC Symbol;Acc:HGNC:8660] |
| AL358115.1 | 26,67 | 6,76 | 1,96 | 1 | 165890795 | 165900683 | - | 1570 | novel transcript, antisense to UCK2 |
| SLC1A6 | 10385,04 | 22016,23 | -1,08 | 19 | 14950034 | 15022990 | - | 7646 | solute carrier family 1 member 6 [Source:HGNC Symbol;Acc:HGNC:10944] |
| CES4A | 56,03 | 19,12 | 1,55 | 16 | 66988589 | 67009758 | + | 4340 | carboxylesterase 4A [Source:HGNC Symbol;Acc:HGNC:26741] |
| PRR34-AS1 | 47,13 | 114,69 | -1,28 | 22 | 46053705 | 46057210 | + | 1093 | PRR34 antisense RNA 1 [Source:HGNC Symbol;Acc:HGNC:50499] |
| IL6R | 198,33 | 86,58 | 1,19 | 1 | 154405193 | 154469450 | + | 6453 | interleukin 6 receptor [Source:HGNC Symbol;Acc:HGNC:6019] |
| AC040970.1 | 57,80 | 20,25 | 1,51 | 8 | 141126044 | 141129961 | - | 807 | novel transcript |
| KRT86 | 162,76 | 357,54 | -1,13 | 12 | 52249300 | 52309163 | + | 2394 | keratin 86 [Source:HGNC Symbol;Acc:HGNC:6463] |
| CCDC78 | 208,12 | 91,08 | 1,19 | 16 | 722582 | 726954 | - | 4147 | coiled-coil domain containing 78 [Source:HGNC Symbol;Acc:HGNC:14153] |
| ZNF442 | 46,24 | 15,75 | 1,55 | 19 | 12345949 | 12365905 | - | 6569 | zinc finger protein 442 [Source:HGNC Symbol;Acc:HGNC:20877] |
| TF | 16,00 | 47,23 | -1,55 | 3 | 133745956 | 133796640 | + | 26199 | transferrin [Source:HGNC Symbol;Acc:HGNC:11740] |
| ANOS1 | 176,99 | 76,46 | 1,21 | X | 8528874 | 8732186 | - | 7131 | anosmin 1 [Source:HGNC Symbol;Acc:HGNC:6211] |
| BEND6 | 353,10 | 160,79 | 1,13 | 6 | 56955126 | 57027342 | + | 5588 | BEN domain containing 6 [Source:HGNC Symbol;Acc:HGNC:20871] |
| A4GALT | 200,11 | 436,24 | -1,12 | 22 | 42692112 | 42721298 | - | 3407 | alpha 1,4-galactosyltransferase (P blood group) [Source:HGNC Symbol;Acc:HGNC:18149] |
| AL390039.1 | 28,45 | 7,88 | 1,84 | X | 107131659 | 107132269 | + | 611 | general transcription factor IIIC, polypeptide 6, alpha 35kDa (GTF3C6) pseudogene |
| MUC4 | 21,34 | 4,51 | 2,22 | 3 | 195746765 | 195812277 | - | 21325 | mucin 4, cell surface associated [Source:HGNC Symbol;Acc:HGNC:7514] |
| HOPX | 21,34 | 4,51 | 2,22 | 4 | 56647988 | 56681899 | - | 7753 | HOP homeobox [Source:HGNC Symbol;Acc:HGNC:24961] |
| AKR1C3 | 906,33 | 425,00 | 1,09 | 10 | 5035354 | 5107686 | + | 4532 | aldo-keto reductase family 1 member C3 [Source:HGNC Symbol;Acc:HGNC:386] |
| AC009948.1 | 50,69 | 18,00 | 1,49 | 2 | 178413939 | 178440243 | + | 4001 | uncharacterized LOC101927027 [Source:NCBI gene;Acc:101927027] |
| CRYBA2 | 50,69 | 18,00 | 1,49 | 2 | 218990189 | 218993421 | - | 1454 | crystallin beta A2 [Source:HGNC Symbol;Acc:HGNC:2395] |
| LINC00607 | 87,16 | 197,89 | -1,18 | 2 | 215611563 | 215843722 | - | 5628 | long intergenic non-protein coding RNA 607 [Source:HGNC Symbol;Acc:HGNC:43944] |
| KRT16P2 | 16,89 | 2,26 | 2,84 | 17 | 16829999 | 16832830 | - | 1500 | keratin 16 pseudogene 2 [Source:HGNC Symbol;Acc:HGNC:37807] |
| CCDC194 | 3,55 | 16,87 | -2,21 | 19 | 17390509 | 17394158 | - | 1464 | coiled-coil domain containing 194 [Source:HGNC Symbol;Acc:HGNC:53438] |
| SH2D6 | 16,89 | 2,26 | 2,84 | 2 | 85418721 | 85437029 | + | 3858 | SH2 domain containing 6 [Source:HGNC Symbol;Acc:HGNC:30439] |
| AC008894.2 | 16,89 | 2,26 | 2,84 | 19 | 16123661 | 16139892 | - | 3927 | novel transcript, antisense RAB8A |
| DDT | 3,55 | 16,87 | -2,21 | 22 | 23971365 | 23979828 | - | 1503 | D-dopachrome tautomerase [Source:HGNC Symbol;Acc:HGNC:2732] |
| AP001574.1 | 3,55 | 16,87 | -2,21 | 8 | 100337595 | 100350707 | + | 546 | novel transcript |
| RSPH14 | 3,55 | 16,87 | -2,21 | 22 | 23059415 | 23145021 | - | 2436 | radial spoke head 14 homolog [Source:HGNC Symbol;Acc:HGNC:13437] |
| RF00019 | 16,89 | 2,26 | 2,84 | 22 | 37013225 | 37013319 | + | 95 |  |
| LEMD1 | 552,33 | 1172,66 | -1,09 | 1 | 205381378 | 205457091 | - | 2199 | LEM domain containing 1 [Source:HGNC Symbol;Acc:HGNC:18725] |
| DLG2 | 39,13 | 12,38 | 1,65 | 11 | 83455012 | 85627922 | - | 16709 | discs large MAGUK scaffold protein 2 [Source:HGNC Symbol;Acc:HGNC:2901] |
| SNORD12B | 39,13 | 12,38 | 1,65 | 20 | 49280319 | 49280409 | + | 91 | small nucleolar RNA, C/D box 12B [Source:HGNC Symbol;Acc:HGNC:33573] |
| RARRES3 | 3338,93 | 7004,46 | -1,07 | 11 | 63536809 | 63546462 | + | 1299 | retinoic acid receptor responder 3 [Source:HGNC Symbol;Acc:HGNC:9869] |
| CSF1R | 325,52 | 148,42 | 1,13 | 5 | 150053291 | 150113372 | - | 5151 | colony stimulating factor 1 receptor [Source:HGNC Symbol;Acc:HGNC:2433] |
| AC092053.3 | 30,23 | 9,00 | 1,74 | 3 | 39148281 | 39172952 | + | 4975 | novel transcript, antisense CSRNP1 |
| CLEC18B | 30,23 | 9,00 | 1,74 | 16 | 74408270 | 74421953 | - | 3143 | C-type lectin domain family 18 member B [Source:HGNC Symbol;Acc:HGNC:33849] |
| NETO2 | 4060,26 | 1942,82 | 1,06 | 16 | 47077703 | 47143997 | - | 7481 | neuropilin and tolloid like 2 [Source:HGNC Symbol;Acc:HGNC:14644] |
| AC092171.5 | 6,22 | 24,75 | -1,97 | 7 | 5428731 | 5429672 | + | 942 | novel transcript |
| AC090617.5 | 6,22 | 24,75 | -1,97 | 17 | 2043475 | 2044968 | - | 443 | novel transcript |
| CD38 | 6,22 | 24,75 | -1,97 | 4 | 15778275 | 15853230 | + | 6855 | CD38 molecule [Source:HGNC Symbol;Acc:HGNC:1667] |
| AC004596.1 | 6,22 | 24,75 | -1,97 | 17 | 44198882 | 44216565 | + | 528 | novel transcript, antisense to ATXN7L3 |
| CYP4F23P | 229,46 | 491,33 | -1,10 | 19 | 15564074 | 15584709 | + | 1579 | cytochrome P450 family 4 subfamily F member 23, pseudogene [Source:HGNC Symbol;Acc:HGNC:39944] |
| AL139100.1 | 30,23 | 76,46 | -1,33 | 6 | 34744176 | 34744673 | + | 498 | ribosomal protein S10 (RPS10) pseudogene |
| SNHG28 | 32,90 | 10,13 | 1,69 | 1 | 159834474 | 159873053 | - | 8100 | small nucleolar RNA host gene 28 [Source:NCBI gene;Acc:284677] |
| H1F0 | 3153,04 | 6555,86 | -1,06 | 22 | 37805093 | 37807436 | + | 2344 | H1 histone family member 0 [Source:HGNC Symbol;Acc:HGNC:4714] |
| FAT3 | 106,72 | 44,98 | 1,24 | 11 | 92352096 | 92896470 | + | 19280 | FAT atypical cadherin 3 [Source:HGNC Symbol;Acc:HGNC:23112] |
| WNT3A | 84,49 | 188,89 | -1,16 | 1 | 228007051 | 228061260 | + | 2932 | Wnt family member 3A [Source:HGNC Symbol;Acc:HGNC:15983] |
| LINC02532 | 24,89 | 65,22 | -1,38 | 6 | 106717452 | 106787541 | - | 6750 | long intergenic non-protein coding RNA 2532 [Source:HGNC Symbol;Acc:HGNC:53549] |
| HOXB2 | 101,39 | 223,75 | -1,14 | 17 | 48540894 | 48544989 | - | 2336 | homeobox B2 [Source:HGNC Symbol;Acc:HGNC:5113] |
| TSHZ2 | 87,16 | 35,99 | 1,27 | 20 | 52972407 | 53495330 | + | 12581 | teashirt zinc finger homeobox 2 [Source:HGNC Symbol;Acc:HGNC:13010] |
| GCNT3 | 286,39 | 131,55 | 1,12 | 15 | 59594875 | 59640239 | + | 6475 | glucosaminyl (N-acetyl) transferase 3, mucin type [Source:HGNC Symbol;Acc:HGNC:4205] |
| ZBTB40-IT1 | 25,78 | 6,76 | 1,92 | 1 | 22517474 | 22519708 | + | 273 | ZBTB40 intronic transcript 1 [Source:HGNC Symbol;Acc:HGNC:41493] |
| RAB34 | 92,49 | 204,63 | -1,14 | 17 | 28714281 | 28718429 | - | 3325 | RAB34, member RAS oncogene family [Source:HGNC Symbol;Acc:HGNC:16519] |
| AL359922.2 | 68,48 | 26,99 | 1,34 | 9 | 21858910 | 21861926 | - | 3017 | novel transcript, antisense MTAP |
| IFI27L2 | 4,44 | 19,12 | -2,07 | 14 | 94127779 | 94130253 | - | 1662 | interferon alpha inducible protein 27 like 2 [Source:HGNC Symbol;Acc:HGNC:19753] |
| LINC00973 | 13,33 | 2,26 | 2,50 | 3 | 98981058 | 98983096 | + | 962 | long intergenic non-protein coding RNA 973 [Source:HGNC Symbol;Acc:HGNC:48868] |
| PDE2A | 13,33 | 2,26 | 2,50 | 11 | 72576141 | 72674591 | - | 7182 | phosphodiesterase 2A [Source:HGNC Symbol;Acc:HGNC:8777] |
| AC027020.2 | 2,66 | 14,63 | -2,40 | 15 | 100547765 | 100550153 | - | 2389 | novel transcript |
| AP002851.1 | 1,77 | 14,63 | -2,95 | 8 | 102656464 | 102687118 | + | 2130 | vegetative cell wall protein gp1-like [Source:NCBI gene;Acc:101927245] |
| ALPK2 | 14,22 | 2,26 | 2,60 | 18 | 58481247 | 58628957 | - | 9431 | alpha kinase 2 [Source:HGNC Symbol;Acc:HGNC:20565] |
| AL391056.1 | 13,33 | 2,26 | 2,50 | 9 | 129575409 | 129584556 | + | 2449 | novel transcript (FLJ20378) |
| C9orf43 | 2,66 | 14,63 | -2,40 | 9 | 113410054 | 113429684 | + | 2290 | chromosome 9 open reading frame 43 [Source:HGNC Symbol;Acc:HGNC:23570] |
| AL031432.4 | 1,77 | 14,63 | -2,95 | 1 | 25232586 | 25234775 | + | 2190 | novel transcript |
| HMGB2P1 | 13,33 | 2,26 | 2,50 | 19 | 1203209 | 1203842 | + | 634 | high mobility group box 2 pseudogene 1 [Source:HGNC Symbol;Acc:HGNC:39174] |
| CALML6 | 14,22 | 2,26 | 2,60 | 1 | 1914827 | 1917296 | + | 2321 | calmodulin like 6 [Source:HGNC Symbol;Acc:HGNC:24193] |
| AC025430.1 | 14,22 | 2,26 | 2,60 | 15 | 44516650 | 44517483 | - | 498 | novel transcript, antisense to CTDSPL2 |
| SERPINA6 | 14,22 | 2,26 | 2,60 | 14 | 94304248 | 94323394 | - | 1852 | serpin family A member 6 [Source:HGNC Symbol;Acc:HGNC:1540] |
| AC008993.1 | 13,33 | 2,26 | 2,50 | 19 | 68403 | 69178 | + | 776 | TEC |
| NTNG1 | 38,24 | 12,38 | 1,62 | 1 | 107140007 | 107483458 | + | 9138 | netrin G1 [Source:HGNC Symbol;Acc:HGNC:23319] |
| C19orf18 | 8,00 | 28,12 | -1,80 | 19 | 57958437 | 57974534 | - | 915 | chromosome 19 open reading frame 18 [Source:HGNC Symbol;Acc:HGNC:28642] |
| ZBED2 | 57,80 | 21,37 | 1,43 | 3 | 111592900 | 111595443 | - | 2311 | zinc finger BED-type containing 2 [Source:HGNC Symbol;Acc:HGNC:20710] |
| COL13A1 | 349,54 | 163,04 | 1,10 | 10 | 69801867 | 69964275 | + | 5888 | collagen type XIII alpha 1 chain [Source:HGNC Symbol;Acc:HGNC:2190] |
| TFCP2L1 | 7175,95 | 3499,98 | 1,04 | 2 | 121216587 | 121285207 | - | 9372 | transcription factor CP2 like 1 [Source:HGNC Symbol;Acc:HGNC:17925] |
| CSF2 | 8,00 | 0,00 | 6,03 | 5 | 132073790 | 132076170 | + | 787 | colony stimulating factor 2 [Source:HGNC Symbol;Acc:HGNC:2434] |
| MGC12916 | 8,00 | 0,00 | 6,03 | 17 | 14303854 | 14305505 | + | 1652 | uncharacterized protein MGC12916 [Source:NCBI gene;Acc:84815] |
| TMEM71 | 8,00 | 0,00 | 6,03 | 8 | 132685007 | 132760712 | - | 3332 | transmembrane protein 71 [Source:HGNC Symbol;Acc:HGNC:26572] |
| STPG4 | 8,00 | 0,00 | 6,03 | 2 | 47045538 | 47155378 | - | 3273 | sperm-tail PG-rich repeat containing 4 [Source:HGNC Symbol;Acc:HGNC:26850] |
| LINC02101 | 8,00 | 0,00 | 6,03 | 5 | 58108082 | 58122341 | - | 725 | long intergenic non-protein coding RNA 2101 [Source:HGNC Symbol;Acc:HGNC:52956] |
| AL606534.1 | 8,00 | 0,00 | 6,03 | 1 | 243135898 | 243140588 | + | 694 | novel transcript |
| AC132192.1 | 0,00 | 7,88 | -6,01 | 11 | 9430356 | 9433486 | - | 597 | novel transcript |
| AL590723.1 | 0,00 | 7,88 | -6,01 | 1 | 176017277 | 176018760 | + | 629 | novel transcript |
| AC024933.1 | 0,00 | 7,88 | -6,01 | 3 | 139349024 | 139349371 | - | 348 | novel transcript, antisense to MRPS22 |
| TREH | 8,00 | 0,00 | 6,03 | 11 | 118657316 | 118679690 | - | 3182 | trehalase [Source:HGNC Symbol;Acc:HGNC:12266] |
| POLR3GP1 | 0,00 | 7,88 | -6,01 | 14 | 91603173 | 91603433 | - | 261 | RNA polymerase III subunit G pseudogene 1 [Source:HGNC Symbol;Acc:HGNC:23345] |
| LEFTY1 | 0,00 | 7,88 | -6,01 | 1 | 225886282 | 225911382 | - | 1891 | left-right determination factor 1 [Source:HGNC Symbol;Acc:HGNC:6552] |
| LINC00466 | 0,00 | 7,88 | -6,01 | 1 | 63159087 | 63317274 | - | 4490 | long intergenic non-protein coding RNA 466 [Source:HGNC Symbol;Acc:HGNC:27294] |
| ISLR2 | 8,00 | 0,00 | 6,03 | 15 | 74100311 | 74138540 | + | 6101 | immunoglobulin superfamily containing leucine rich repeat 2 [Source:HGNC Symbol;Acc:HGNC:29286] |
| LINC01932 | 8,00 | 0,00 | 6,03 | 5 | 159227715 | 159245127 | + | 573 | long intergenic non-protein coding RNA 1932 [Source:HGNC Symbol;Acc:HGNC:52755] |
| LGI2 | 8,00 | 0,00 | 6,03 | 4 | 24998847 | 25030879 | - | 6428 | leucine rich repeat LGI family member 2 [Source:HGNC Symbol;Acc:HGNC:18710] |
| AC226118.1 | 0,00 | 7,88 | -6,01 | 7 | 379359 | 382712 | + | 2146 | uncharacterized LOC442497 [Source:NCBI gene;Acc:442497] |
| FDPSP2 | 8,00 | 0,00 | 6,03 | 7 | 76470162 | 76471058 | - | 897 | farnesyl diphosphate synthase pseudogene 2 [Source:HGNC Symbol;Acc:HGNC:3633] |
| AC103739.2 | 0,00 | 7,88 | -6,01 | 15 | 90604225 | 90614558 | - | 807 | novel transcript, antisense to CRTC3 |
| RAB40AL | 8,00 | 0,00 | 6,03 | X | 102937272 | 102938300 | + | 1029 | RAB40A like [Source:HGNC Symbol;Acc:HGNC:25410] |
| AL442067.2 | 0,00 | 7,88 | -6,01 | 13 | 97437268 | 97437630 | + | 363 | novel transcript, sense intronic to RAP2A |
| LINC01962 | 8,00 | 0,00 | 6,03 | 5 | 181191046 | 181191852 | - | 411 | long intergenic non-protein coding RNA 1962 [Source:HGNC Symbol;Acc:HGNC:52787] |
| ADGRF2 | 8,00 | 0,00 | 6,03 | 6 | 47656436 | 47697797 | + | 6395 | adhesion G protein-coupled receptor F2 [Source:HGNC Symbol;Acc:HGNC:18991] |
| AL772284.1 | 0,00 | 7,88 | -6,01 | X | 118938153 | 118939134 | + | 905 | pseudogene similar to part of KIAA0633 protein (COBL) |
| AL157871.2 | 8,00 | 0,00 | 6,03 | 14 | 100339832 | 100340554 | + | 552 | novel transcript, antisense to WARS |
| AL603840.1 | 8,00 | 0,00 | 6,03 | 1 | 55329288 | 56070513 | + | 6257 | novel transcript |
| MUCL3 | 0,00 | 7,88 | -6,01 | 6 | 30934523 | 30954221 | + | 5682 | mucin like 3 [Source:HGNC Symbol;Acc:HGNC:21666] |
| MFSD13B | 0,00 | 7,88 | -6,01 | 16 | 22382436 | 22407428 | + | 1290 | major facilitator superfamily domain containing 13B (pseudogene) [Source:HGNC Symbol;Acc:HGNC:52163] |
| RASL11A | 238,36 | 501,45 | -1,07 | 13 | 27270327 | 27273690 | + | 1624 | RAS like family 11 member A [Source:HGNC Symbol;Acc:HGNC:23802] |
| NCMAP | 191,22 | 404,76 | -1,08 | 1 | 24556111 | 24609328 | + | 4122 | non-compact myelin associated protein [Source:HGNC Symbol;Acc:HGNC:29332] |
| ROS1 | 172,54 | 77,59 | 1,15 | 6 | 117288300 | 117425855 | - | 7856 | ROS proto-oncogene 1, receptor tyrosine kinase [Source:HGNC Symbol;Acc:HGNC:10261] |
| SLC7A2 | 8,88 | 30,37 | -1,76 | 8 | 17497088 | 17570573 | + | 7905 | solute carrier family 7 member 2 [Source:HGNC Symbol;Acc:HGNC:11060] |
| DCLK1 | 8,88 | 30,37 | -1,76 | 13 | 35768652 | 36131306 | - | 14375 | doublecortin like kinase 1 [Source:HGNC Symbol;Acc:HGNC:2700] |
| TEX45 | 8,88 | 30,37 | -1,76 | 19 | 7492976 | 7508450 | + | 2912 | testis expressed 45 [Source:HGNC Symbol;Acc:HGNC:24745] |
| AP001350.1 | 29,34 | 9,00 | 1,69 | 11 | 58611119 | 58612642 | - | 1524 | novel transcript, antisense to ZFP91 |
| IL18R1 | 29,34 | 9,00 | 1,69 | 2 | 102311529 | 102398775 | + | 4353 | interleukin 18 receptor 1 [Source:HGNC Symbol;Acc:HGNC:5988] |
| MAP3K20-AS1 | 8,88 | 30,37 | -1,76 | 2 | 173166446 | 173282036 | - | 3049 | MAP3K20 antisense RNA 1 [Source:HGNC Symbol;Acc:HGNC:27935] |
| AK3P3 | 8,88 | 30,37 | -1,76 | 7 | 23129178 | 23129841 | + | 664 | adenylate kinase 3 pseudogene 3 [Source:HGNC Symbol;Acc:HGNC:39062] |
| NRXN1 | 9,77 | 30,37 | -1,62 | 2 | 49918505 | 51225575 | - | 24320 | neurexin 1 [Source:HGNC Symbol;Acc:HGNC:8008] |
| TH | 9,77 | 30,37 | -1,62 | 11 | 2163929 | 2171877 | - | 3243 | tyrosine hydroxylase [Source:HGNC Symbol;Acc:HGNC:11782] |
| AC010531.6 | 5,33 | 21,37 | -1,98 | 16 | 87317509 | 87318043 | + | 401 | uncharacterized LOC101928659 [Source:NCBI gene;Acc:101928659] |
| HYDIN2 | 20,45 | 4,51 | 2,15 | 1 | 146472566 | 146914294 | + | 17641 | HYDIN2, axonemal central pair apparatus protein (pseudogene) [Source:HGNC Symbol;Acc:HGNC:33129] |
| TFAP2B | 5,33 | 21,37 | -1,98 | 6 | 50818723 | 50847613 | + | 6149 | transcription factor AP-2 beta [Source:HGNC Symbol;Acc:HGNC:11743] |
| ITIH6 | 5,33 | 21,37 | -1,98 | X | 54748899 | 54798240 | - | 5497 | inter-alpha-trypsin inhibitor heavy chain family member 6 [Source:HGNC Symbol;Acc:HGNC:28907] |
| AL024498.1 | 44,46 | 15,75 | 1,49 | 6 | 10743324 | 10747663 | - | 1471 | novel transcript |
| PALM | 199,22 | 419,38 | -1,07 | 19 | 708939 | 748329 | + | 4908 | paralemmin [Source:HGNC Symbol;Acc:HGNC:8594] |
| LINC01094 | 10,66 | 1,13 | 3,11 | 4 | 78645903 | 78682699 | + | 2951 | long intergenic non-protein coding RNA 1094 [Source:HGNC Symbol;Acc:HGNC:49219] |
| AL021918.4 | 10,66 | 1,13 | 3,11 | 6 | 27454568 | 27457575 | + | 1591 | novel transcript, antisense to ZNF184 |
| GPR61 | 10,66 | 1,13 | 3,11 | 1 | 109539872 | 109548406 | + | 5057 | G protein-coupled receptor 61 [Source:HGNC Symbol;Acc:HGNC:13300] |
| AC093525.7 | 10,66 | 1,13 | 3,11 | 16 | 2561471 | 2565096 | - | 3626 | novel transcript, antisense to PDPK1 |
| AL627309.2 | 0,88 | 11,25 | -3,49 | 1 | 139790 | 140339 | - | 323 | novel transcript |
| AC124276.1 | 10,66 | 1,13 | 3,11 | 11 | 12066929 | 12073014 | + | 899 | novel transcript |
| FAM96AP2 | 0,88 | 11,25 | -3,49 | 1 | 228114997 | 228115473 | - | 477 | family with sequence similarity 96 member A pseudogene 2 [Source:HGNC Symbol;Acc:HGNC:43862] |
| OR2L2 | 10,66 | 1,13 | 3,11 | 1 | 248030070 | 248042305 | + | 4375 | olfactory receptor family 2 subfamily L member 2 [Source:HGNC Symbol;Acc:HGNC:8266] |
| FAM221B | 10,66 | 1,13 | 3,11 | 9 | 35816391 | 35828747 | - | 3941 | family with sequence similarity 221 member B [Source:HGNC Symbol;Acc:HGNC:30762] |
| TNRC18P1 | 0,88 | 11,25 | -3,49 | 4 | 140641840 | 140645489 | - | 3650 | trinucleotide repeat containing 18 pseudogene 1 [Source:HGNC Symbol;Acc:HGNC:43881] |
| P2RY4 | 0,88 | 11,25 | -3,49 | X | 70258170 | 70259764 | - | 1595 | pyrimidinergic receptor P2Y4 [Source:HGNC Symbol;Acc:HGNC:8542] |
| LINC00898 | 10,66 | 1,13 | 3,11 | 22 | 47621043 | 47631569 | - | 4314 | long intergenic non-protein coding RNA 898 [Source:HGNC Symbol;Acc:HGNC:48581] |
| AC005005.3 | 10,66 | 1,13 | 3,11 | 22 | 31082156 | 31083565 | - | 1410 | novel transcript, antisense to SMTN |
| LINC00623 | 17,78 | 47,23 | -1,40 | 1 | 120913275 | 121009291 | + | 2075 | long intergenic non-protein coding RNA 623 [Source:HGNC Symbol;Acc:HGNC:44252] |
| TMEM221 | 17,78 | 47,23 | -1,40 | 19 | 17435509 | 17448567 | - | 2317 | transmembrane protein 221 [Source:HGNC Symbol;Acc:HGNC:21943] |
| PDLIM2 | 274,83 | 571,16 | -1,05 | 8 | 22578279 | 22598025 | + | 12388 | PDZ and LIM domain 2 [Source:HGNC Symbol;Acc:HGNC:13992] |
| FRY | 620,82 | 1274,98 | -1,04 | 13 | 31846713 | 32299122 | + | 18849 | FRY microtubule binding protein [Source:HGNC Symbol;Acc:HGNC:20367] |
| RASGRP3 | 163,65 | 73,09 | 1,16 | 2 | 33436324 | 33564750 | + | 11118 | RAS guanyl releasing protein 3 [Source:HGNC Symbol;Acc:HGNC:14545] |
| LINC01559 | 220,57 | 101,20 | 1,12 | 12 | 13371089 | 13387167 | - | 3953 | long intergenic non-protein coding RNA 1559 [Source:HGNC Symbol;Acc:HGNC:26598] |
| SLC40A1 | 32,01 | 10,13 | 1,65 | 2 | 189560579 | 189583758 | - | 4330 | solute carrier family 40 member 1 [Source:HGNC Symbol;Acc:HGNC:10909] |
| AC020907.4 | 48,91 | 18,00 | 1,44 | 19 | 35014961 | 35025335 | - | 526 | novel transcript |
| ETV7 | 137,85 | 292,33 | -1,08 | 6 | 36354091 | 36387800 | - | 2374 | ETS variant 7 [Source:HGNC Symbol;Acc:HGNC:18160] |
| MATN2 | 563,89 | 270,97 | 1,06 | 8 | 97868840 | 98036716 | + | 6933 | matrilin 2 [Source:HGNC Symbol;Acc:HGNC:6908] |
| FAM83A | 144,97 | 64,10 | 1,18 | 8 | 123178960 | 123210079 | + | 6317 | family with sequence similarity 83 member A [Source:HGNC Symbol;Acc:HGNC:28210] |
| CAVIN4 | 23,12 | 5,63 | 2,02 | 9 | 100578079 | 100587906 | + | 2615 | caveolae associated protein 4 [Source:HGNC Symbol;Acc:HGNC:33742] |
| B4GALNT2 | 22,23 | 5,63 | 1,96 | 17 | 49132460 | 49176840 | + | 8961 | beta-1,4-N-acetyl-galactosaminyltransferase 2 [Source:HGNC Symbol;Acc:HGNC:24136] |
| WNT11 | 23,12 | 5,63 | 2,02 | 11 | 76186325 | 76210736 | - | 2401 | Wnt family member 11 [Source:HGNC Symbol;Acc:HGNC:12776] |
| NCAM1 | 170,76 | 77,59 | 1,14 | 11 | 112961247 | 113278436 | + | 12737 | neural cell adhesion molecule 1 [Source:HGNC Symbol;Acc:HGNC:7656] |
| PLA2G16 | 1850,02 | 3743,96 | -1,02 | 11 | 63573195 | 63616883 | - | 3116 | phospholipase A2 group XVI [Source:HGNC Symbol;Acc:HGNC:17825] |
| DNAJC19P5 | 60,47 | 23,62 | 1,35 | 2 | 177229191 | 177229506 | - | 316 | DnaJ heat shock protein family (Hsp40) member C19 pseudogene 5 [Source:HGNC Symbol;Acc:HGNC:45068] |
| ABAT | 198,33 | 91,08 | 1,12 | 16 | 8674565 | 8784575 | + | 9744 | 4-aminobutyrate aminotransferase [Source:HGNC Symbol;Acc:HGNC:23] |
| PSG9 | 50,69 | 114,69 | -1,18 | 19 | 43211791 | 43269530 | - | 5048 | pregnancy specific beta-1-glycoprotein 9 [Source:HGNC Symbol;Acc:HGNC:9526] |
| CLIC3 | 53,36 | 120,31 | -1,17 | 9 | 136994635 | 136996803 | - | 1177 | chloride intracellular channel 3 [Source:HGNC Symbol;Acc:HGNC:2064] |
| MUCL1 | 37,35 | 12,38 | 1,59 | 12 | 54830519 | 54858393 | + | 1250 | mucin like 1 [Source:HGNC Symbol;Acc:HGNC:30588] |
| DCLK3 | 40,02 | 13,50 | 1,56 | 3 | 36712422 | 36764349 | - | 5574 | doublecortin like kinase 3 [Source:HGNC Symbol;Acc:HGNC:19005] |
| LINC00662 | 1382,17 | 2787,17 | -1,01 | 19 | 27684580 | 27793940 | - | 7958 | long intergenic non-protein coding RNA 662 [Source:HGNC Symbol;Acc:HGNC:27122] |
| GRAMD1B | 1305,68 | 643,12 | 1,02 | 11 | 123358428 | 123627774 | + | 14863 | GRAM domain containing 1B [Source:HGNC Symbol;Acc:HGNC:29214] |
| FLNC | 24,89 | 6,76 | 1,87 | 7 | 128830377 | 128859274 | + | 9280 | filamin C [Source:HGNC Symbol;Acc:HGNC:3756] |
| JAKMIP3 | 24,89 | 6,76 | 1,87 | 10 | 132104671 | 132184809 | + | 7608 | Janus kinase and microtubule interacting protein 3 [Source:HGNC Symbol;Acc:HGNC:23523] |
| CLDN10 | 586,13 | 1188,41 | -1,02 | 13 | 95433604 | 95579759 | + | 3380 | claudin 10 [Source:HGNC Symbol;Acc:HGNC:2033] |
| NBPF4 | 96,94 | 208,01 | -1,10 | 1 | 108223341 | 108244081 | - | 2586 | NBPF member 4 [Source:HGNC Symbol;Acc:HGNC:26550] |
| THEMIS2 | 19,56 | 50,60 | -1,36 | 1 | 27872543 | 27886685 | + | 4621 | thymocyte selection associated family member 2 [Source:HGNC Symbol;Acc:HGNC:16839] |
| FAM225A | 17,78 | 3,38 | 2,36 | 9 | 113113073 | 113119928 | + | 6100 | family with sequence similarity 225 member A [Source:HGNC Symbol;Acc:HGNC:27855] |
| C1orf229 | 4,44 | 18,00 | -1,99 | 1 | 247110160 | 247112417 | - | 2258 | chromosome 1 open reading frame 229 [Source:HGNC Symbol;Acc:HGNC:33759] |
| AL031777.3 | 4,44 | 18,00 | -1,99 | 6 | 26195595 | 26199293 | - | 2110 | novel protein |
| SGCD | 8,88 | 29,24 | -1,70 | 5 | 155870344 | 156767788 | + | 10584 | sarcoglycan delta [Source:HGNC Symbol;Acc:HGNC:10807] |
| LAMA3 | 1605,42 | 801,64 | 1,00 | 18 | 23689443 | 23956222 | + | 14678 | laminin subunit alpha 3 [Source:HGNC Symbol;Acc:HGNC:6483] |
| AC011448.1 | 67,59 | 28,12 | 1,26 | 19 | 19516227 | 19536076 | + | 1846 | readthrough between NDUFA13 and YJEFN3 |
| AP002884.1 | 71,15 | 29,24 | 1,28 | 11 | 112270749 | 112362534 | + | 5018 | uncharacterized LOC283140 [Source:NCBI gene;Acc:283140] |
| AC012615.6 | 131,63 | 59,60 | 1,14 | 19 | 1822089 | 1824542 | + | 2454 | uncharacterized LOC100288123 [Source:NCBI gene;Acc:100288123] |
| DSTNP1 | 77,37 | 32,62 | 1,24 | 21 | 46653558 | 46654022 | - | 465 | destrin, actin depolymerizing factor pseudogene 1 [Source:HGNC Symbol;Acc:HGNC:23769] |
| EML5 | 276,61 | 132,68 | 1,06 | 14 | 88612431 | 88792752 | - | 11000 | echinoderm microtubule associated protein like 5 [Source:HGNC Symbol;Acc:HGNC:18197] |
| TONSL-AS1 | 31,12 | 10,13 | 1,61 | 8 | 144437675 | 144439971 | + | 428 | TONSL antisense RNA 1 [Source:HGNC Symbol;Acc:HGNC:51556] |
| PLEKHD1 | 11,55 | 33,74 | -1,53 | 14 | 69484692 | 69531551 | + | 8097 | pleckstrin homology and coiled-coil domain containing D1 [Source:HGNC Symbol;Acc:HGNC:20148] |
| PLA2G4B | 12,44 | 2,26 | 2,41 | 15 | 41837775 | 41848147 | + | 4446 | phospholipase A2 group IVB [Source:HGNC Symbol;Acc:HGNC:9036] |
| MAPK8IP1P2 | 2,66 | 13,50 | -2,29 | 17 | 45600869 | 45602340 | - | 1472 | mitogen-activated protein kinase 8 interacting protein 1 pseudogene 2 [Source:HGNC Symbol;Acc:HGNC:52402] |
| AC011462.4 | 2,66 | 13,50 | -2,29 | 19 | 41373971 | 41374419 | + | 449 | novel transcript, sense intronic TMEM91 |
| HPCA | 12,44 | 2,26 | 2,41 | 1 | 32885994 | 32898441 | + | 1991 | hippocalcin [Source:HGNC Symbol;Acc:HGNC:5144] |
| AC010422.2 | 12,44 | 2,26 | 2,41 | 19 | 12682693 | 12687279 | - | 635 | novel transcript |
| APOBEC2 | 2,66 | 13,50 | -2,29 | 6 | 41053304 | 41064511 | + | 1151 | apolipoprotein B mRNA editing enzyme catalytic subunit 2 [Source:HGNC Symbol;Acc:HGNC:605] |
| SLC26A4-AS1 | 12,44 | 2,26 | 2,41 | 7 | 107653968 | 107662151 | - | 5029 | SLC26A4 antisense RNA 1 [Source:HGNC Symbol;Acc:HGNC:22385] |
| AL139156.2 | 2,66 | 13,50 | -2,29 | 1 | 52160261 | 52160600 | - | 340 | pseudogene similar to part of alcohol dehydrogenase 5 (class III), chi polypeptidem (ADH5) |
| Z83844.2 | 2,66 | 13,50 | -2,29 | 22 | 37641832 | 37658377 | - | 1945 | uncharacterized LOC101927051 [Source:NCBI gene;Acc:101927051] |
| AL032819.2 | 12,44 | 2,26 | 2,41 | 16 | 1437154 | 1439315 | + | 2162 | TEC |
| AC079988.1 | 12,44 | 2,26 | 2,41 | 2 | 121178327 | 121182580 | + | 544 | novel transcript |
| DUTP6 | 5,33 | 20,25 | -1,90 | 1 | 166868748 | 166869209 | + | 462 | deoxyuridine triphosphatase pseudogene 6 [Source:HGNC Symbol;Acc:HGNC:39519] |
| AC004846.1 | 5,33 | 20,25 | -1,90 | 14 | 73242651 | 73245979 | - | 3050 | uncharacterized LOC101928123 [Source:NCBI gene;Acc:101928123] |
| AL109613.1 | 13,33 | 37,11 | -1,47 | 1 | 93592199 | 93605573 | + | 1954 | novel transcript |
| LRRC29 | 13,33 | 37,11 | -1,47 | 16 | 67207139 | 67227048 | - | 2807 | leucine rich repeat containing 29 [Source:HGNC Symbol;Acc:HGNC:13605] |
| C2orf48 | 102,28 | 214,75 | -1,07 | 2 | 10141382 | 10211725 | + | 2502 | chromosome 2 open reading frame 48 [Source:HGNC Symbol;Acc:HGNC:26322] |
| AC004233.4 | 14,22 | 39,36 | -1,46 | 16 | 2981175 | 2981591 | - | 417 | novel transcript |
| CHKB | 118,29 | 53,98 | 1,13 | 22 | 50578949 | 50601455 | - | 3021 | choline kinase beta [Source:HGNC Symbol;Acc:HGNC:1938] |
| GDA | 699,98 | 348,55 | 1,01 | 9 | 72114595 | 72257193 | + | 7904 | guanine deaminase [Source:HGNC Symbol;Acc:HGNC:4212] |
| GPR176 | 112,95 | 50,60 | 1,16 | 15 | 39799032 | 39920892 | - | 5119 | G protein-coupled receptor 176 [Source:HGNC Symbol;Acc:HGNC:32370] |
| AP001207.3 | 51,58 | 113,57 | -1,14 | 8 | 101461177 | 101492499 | - | 491 | novel transcript |
| HSD11B1L | 120,06 | 249,61 | -1,05 | 19 | 5680604 | 5688523 | + | 2991 | hydroxysteroid 11-beta dehydrogenase 1 like [Source:HGNC Symbol;Acc:HGNC:30419] |
| GP1BA | 17,78 | 44,98 | -1,33 | 17 | 4932297 | 4935030 | + | 2501 | glycoprotein Ib platelet subunit alpha [Source:HGNC Symbol;Acc:HGNC:4439] |
| PTGER1 | 18,67 | 47,23 | -1,33 | 19 | 14472466 | 14475362 | - | 1421 | prostaglandin E receptor 1 [Source:HGNC Symbol;Acc:HGNC:9593] |
| RGPD2 | 157,42 | 73,09 | 1,11 | 2 | 87755955 | 87825952 | - | 7023 | RANBP2-like and GRIP domain containing 2 [Source:HGNC Symbol;Acc:HGNC:32415] |
| AP003733.4 | 48,91 | 19,12 | 1,35 | 11 | 61967794 | 61969490 | + | 1697 | uncharacterized LOC399900 [Source:NCBI gene;Acc:399900] |
| INSL3 | 19,56 | 49,48 | -1,33 | 19 | 17816512 | 17821574 | - | 923 | insulin like 3 [Source:HGNC Symbol;Acc:HGNC:6086] |
| MMP14 | 206,34 | 417,13 | -1,01 | 14 | 22836557 | 22849027 | + | 4464 | matrix metallopeptidase 14 [Source:HGNC Symbol;Acc:HGNC:7160] |
| REN | 68,48 | 28,12 | 1,28 | 1 | 204154819 | 204190324 | - | 1734 | renin [Source:HGNC Symbol;Acc:HGNC:9958] |
| DDX47 | 54,25 | 21,37 | 1,34 | 12 | 12813316 | 12829981 | + | 5040 | DEAD-box helicase 47 [Source:HGNC Symbol;Acc:HGNC:18682] |
| LINC01564 | 54,25 | 21,37 | 1,34 | 6 | 53628380 | 53631394 | + | 566 | long intergenic non-protein coding RNA 1564 [Source:HGNC Symbol;Acc:HGNC:51361] |
| AL358472.3 | 21,34 | 5,63 | 1,90 | 1 | 153966516 | 153966930 | + | 415 | novel transcript, antisense to SLC39A1 |
| AC125611.4 | 21,34 | 5,63 | 1,90 | 12 | 49292631 | 49324576 | - | 4166 | uncharacterized LOC101927267 [Source:NCBI gene;Acc:101927267] |
| HCG9 | 21,34 | 5,63 | 1,90 | 6 | 29975112 | 29978410 | + | 700 | HLA complex group 9 [Source:HGNC Symbol;Acc:HGNC:21243] |
| RNF212B | 6,22 | 22,50 | -1,83 | 14 | 23185316 | 23273477 | + | 2059 | ring finger protein 212B [Source:HGNC Symbol;Acc:HGNC:20438] |
| PCDHA1 | 6,22 | 22,50 | -1,83 | 5 | 140786136 | 141012347 | + | 5595 | protocadherin alpha 1 [Source:HGNC Symbol;Acc:HGNC:8663] |
| DOCK10 | 24,01 | 6,76 | 1,81 | 2 | 224765090 | 225042445 | - | 11265 | dedicator of cytokinesis 10 [Source:HGNC Symbol;Acc:HGNC:23479] |
| AL031847.1 | 7,11 | 24,75 | -1,78 | 1 | 6204840 | 6205780 | - | 378 | uncharacterized LOC102724450 [Source:NCBI gene;Acc:102724450] |
| PAICSP1 | 24,01 | 6,76 | 1,81 | 9 | 37878116 | 37879493 | + | 1217 | phosphoribosylaminoimidazole carboxylase, phosphoribosylaminoimidazole succinocarboxamide synthetase pseudogene 1 [Source:HGNC Symbol;Acc:HGNC:8588] |
| RIMS2 | 152,97 | 71,97 | 1,09 | 8 | 103500748 | 104256094 | + | 13619 | regulating synaptic membrane exocytosis 2 [Source:HGNC Symbol;Acc:HGNC:17283] |
| FBXL19-AS1 | 234,80 | 113,57 | 1,05 | 16 | 30919319 | 30923269 | - | 3951 | FBXL19 antisense RNA 1 [Source:HGNC Symbol;Acc:HGNC:27557] |
| FSCN1 | 269,49 | 131,55 | 1,03 | 7 | 5592823 | 5606655 | + | 3692 | fascin actin-bundling protein 1 [Source:HGNC Symbol;Acc:HGNC:11148] |
| FYN | 179,66 | 85,46 | 1,07 | 6 | 111660332 | 111873452 | - | 7411 | FYN proto-oncogene, Src family tyrosine kinase [Source:HGNC Symbol;Acc:HGNC:4037] |
| COL4A1 | 321,08 | 158,54 | 1,02 | 13 | 110148963 | 110307233 | - | 14738 | collagen type IV alpha 1 chain [Source:HGNC Symbol;Acc:HGNC:2202] |
| CYS1 | 139,63 | 283,34 | -1,02 | 2 | 10056780 | 10080944 | - | 2847 | cystin 1 [Source:HGNC Symbol;Acc:HGNC:18525] |
| PIWIL4 | 8,00 | 25,87 | -1,68 | 11 | 94543840 | 94621421 | + | 4558 | piwi like RNA-mediated gene silencing 4 [Source:HGNC Symbol;Acc:HGNC:18444] |
| AC087752.4 | 25,78 | 7,88 | 1,70 | 8 | 94884609 | 94885070 | + | 462 | novel transcript, antisense to CCNE2 |
| LANCL3 | 25,78 | 7,88 | 1,70 | X | 37571569 | 37684463 | + | 10298 | LanC like 3 [Source:HGNC Symbol;Acc:HGNC:24767] |
| SLC6A20 | 85,38 | 37,11 | 1,20 | 3 | 45755450 | 45796535 | - | 6651 | solute carrier family 6 member 20 [Source:HGNC Symbol;Acc:HGNC:30927] |
| ATP10B | 406,46 | 202,39 | 1,01 | 5 | 160563120 | 160852214 | - | 11310 | ATPase phospholipid transporting 10B (putative) [Source:HGNC Symbol;Acc:HGNC:13543] |
| MAGEA2 | 159,20 | 75,34 | 1,08 | X | 152749863 | 152753884 | - | 2272 | MAGE family member A2 [Source:HGNC Symbol;Acc:HGNC:6800] |
| AC015921.1 | 3,55 | 15,75 | -2,11 | 17 | 2962248 | 2965895 | - | 2546 | uncharacterized LOC101927911 [Source:NCBI gene;Acc:101927911] |
| PRODH | 454,49 | 227,12 | 1,00 | 22 | 18912777 | 18936553 | - | 8776 | proline dehydrogenase 1 [Source:HGNC Symbol;Acc:HGNC:9453] |
| GASAL1 | 75,59 | 32,62 | 1,21 | 8 | 102805517 | 102809971 | + | 2829 | growth arrest associated lncRNA 1 [Source:HGNC Symbol;Acc:HGNC:53461] |
| ANXA2R | 8,88 | 28,12 | -1,65 | 5 | 43039081 | 43043170 | - | 2386 | annexin A2 receptor [Source:HGNC Symbol;Acc:HGNC:33463] |
| AC005162.3 | 8,88 | 28,12 | -1,65 | 7 | 28979967 | 29013367 | + | 4916 | uncharacterized LOC100506497 [Source:NCBI gene;Acc:100506497] |
| LINC00624 | 69,37 | 29,24 | 1,24 | 1 | 147258885 | 147517875 | - | 5564 | long intergenic non-protein coding RNA 624 [Source:HGNC Symbol;Acc:HGNC:44254] |
| AC040977.1 | 10,66 | 30,37 | -1,50 | 17 | 6994642 | 6995189 | - | 343 | novel transcript |
| TRAF1 | 144,08 | 67,47 | 1,09 | 9 | 120902393 | 120929173 | - | 6449 | TNF receptor associated factor 1 [Source:HGNC Symbol;Acc:HGNC:12031] |
| NPIPP1 | 61,36 | 25,87 | 1,24 | 16 | 15104312 | 15123498 | - | 1437 | nuclear pore complex interacting protein pseudogene 1 [Source:HGNC Symbol;Acc:HGNC:35407] |
| CUZD1 | 61,36 | 25,87 | 1,24 | 10 | 122832149 | 122850793 | - | 3464 | CUB and zona pellucida like domains 1 [Source:HGNC Symbol;Acc:HGNC:17937] |
| ZEB1 | 9,77 | 1,13 | 2,99 | 10 | 31318495 | 31529814 | + | 8650 | zinc finger E-box binding homeobox 1 [Source:HGNC Symbol;Acc:HGNC:11642] |
| RBMS3 | 9,77 | 1,13 | 2,99 | 3 | 28574791 | 30010391 | + | 18339 | RNA binding motif single stranded interacting protein 3 [Source:HGNC Symbol;Acc:HGNC:13427] |
| CYTH4 | 9,77 | 1,13 | 2,99 | 22 | 37282027 | 37315345 | + | 6161 | cytohesin 4 [Source:HGNC Symbol;Acc:HGNC:9505] |
| AC053513.1 | 9,77 | 1,13 | 2,99 | 12 | 22460519 | 22463914 | - | 738 | novel transcript |
| GDPD2 | 9,77 | 1,13 | 2,99 | X | 70423031 | 70433390 | + | 2638 | glycerophosphodiester phosphodiesterase domain containing 2 [Source:HGNC Symbol;Acc:HGNC:25974] |
| RBP3 | 0,88 | 10,13 | -3,34 | 10 | 47348371 | 47357875 | + | 4276 | retinol binding protein 3 [Source:HGNC Symbol;Acc:HGNC:9921] |
| LMO3 | 9,77 | 1,13 | 2,99 | 12 | 16548373 | 16610594 | - | 10268 | LIM domain only 3 [Source:HGNC Symbol;Acc:HGNC:6643] |
| LINC00345 | 9,77 | 1,13 | 2,99 | 13 | 52482804 | 52489216 | - | 1966 | long intergenic non-protein coding RNA 345 [Source:HGNC Symbol;Acc:HGNC:42503] |
| AC004837.2 | 9,77 | 1,13 | 2,99 | 7 | 39700341 | 39703296 | - | 731 | novel transcript |
| AL359532.1 | 0,88 | 10,13 | -3,34 | 10 | 30831828 | 30833387 | - | 1560 | novel transcript, sense intronic to ZNF438 |
| RN7SL526P | 9,77 | 1,13 | 2,99 | 19 | 58069970 | 58070288 | + | 319 | RNA, 7SL, cytoplasmic 526, pseudogene [Source:HGNC Symbol;Acc:HGNC:46542] |
| FER1L6 | 9,77 | 1,13 | 2,99 | 8 | 123851987 | 124120061 | + | 6051 | fer-1 like family member 6 [Source:HGNC Symbol;Acc:HGNC:28065] |
| XCR1 | 9,77 | 1,13 | 2,99 | 3 | 46017024 | 46027742 | - | 5403 | X-C motif chemokine receptor 1 [Source:HGNC Symbol;Acc:HGNC:1625] |
| C7orf65 | 0,88 | 10,13 | -3,34 | 7 | 47655244 | 47661648 | + | 2911 | chromosome 7 open reading frame 65 [Source:HGNC Symbol;Acc:HGNC:34432] |
| AC008083.2 | 0,88 | 10,13 | -3,34 | 12 | 47248124 | 47257539 | + | 510 | novel transcript |
| DLG3-AS1 | 9,77 | 1,13 | 2,99 | X | 70452958 | 70455994 | - | 482 | DLG3 antisense RNA 1 [Source:HGNC Symbol;Acc:HGNC:40182] |
| RPL7AP2 | 0,88 | 10,13 | -3,34 | 14 | 39156742 | 39157852 | + | 740 | ribosomal protein L7a pseudogene 2 [Source:HGNC Symbol;Acc:HGNC:23552] |
| SNORA74C-1 | 9,77 | 1,13 | 2,99 | 10 | 45984610 | 45984810 | + | 201 | small nucleolar RNA, H/ACA box 74C-1 [Source:HGNC Symbol;Acc:HGNC:52217] |
| SLC22A15 | 139,63 | 65,22 | 1,10 | 1 | 115976498 | 116070054 | + | 5292 | solute carrier family 22 member 15 [Source:HGNC Symbol;Acc:HGNC:20301] |
| AC093525.8 | 57,80 | 23,62 | 1,29 | 16 | 2516658 | 2517999 | + | 1342 | tec |
| LINC00578 | 12,44 | 34,86 | -1,48 | 3 | 177441921 | 177752305 | + | 1234 | long intergenic non-protein coding RNA 578 [Source:HGNC Symbol;Acc:HGNC:43807] |
| AGMAT | 13,33 | 35,99 | -1,42 | 1 | 15572353 | 15585110 | - | 2500 | agmatinase [Source:HGNC Symbol;Acc:HGNC:18407] |
| TBATA | 35,57 | 12,38 | 1,52 | 10 | 70771239 | 70785401 | - | 1717 | thymus, brain and testes associated [Source:HGNC Symbol;Acc:HGNC:23511] |
| LMO7-AS1 | 14,22 | 38,24 | -1,42 | 13 | 75604700 | 75635994 | - | 593 | LMO7 antisense RNA 1 [Source:HGNC Symbol;Acc:HGNC:50277] |
| UNC13A | 41,79 | 15,75 | 1,40 | 19 | 17601328 | 17688365 | - | 11188 | unc-13 homolog A [Source:HGNC Symbol;Acc:HGNC:23150] |
| SLC4A1APP1 | 16,00 | 42,73 | -1,41 | 9 | 30558880 | 30559481 | - | 602 | solute carrier family 4 member 1 adaptor protein pseudogene 1 [Source:HGNC Symbol;Acc:HGNC:49811] |
| MT-TL1 | 154,75 | 73,09 | 1,08 | MT | 3230 | 3304 | + | 75 | mitochondrially encoded tRNA leucine 1 (UUA/G) [Source:HGNC Symbol;Acc:HGNC:7490] |
| PDGFRB | 7,11 | 0,00 | 5,87 | 5 | 150113837 | 150155872 | - | 7137 | platelet derived growth factor receptor beta [Source:HGNC Symbol;Acc:HGNC:8804] |
| AC136475.2 | 0,00 | 6,76 | -5,79 | 11 | 310139 | 311141 | - | 392 | novel transcript |
| FTLP3 | 7,11 | 0,00 | 5,87 | 20 | 4023917 | 4024444 | + | 528 | ferritin light chain pseudogene 3 [Source:HGNC Symbol;Acc:HGNC:4000] |
| AC027088.3 | 7,11 | 0,00 | 5,87 | 15 | 69037549 | 69043565 | - | 872 | novel transcript, antisense to NOX5 |
| AC087163.3 | 7,11 | 0,00 | 5,87 | 17 | 18026072 | 18026771 | - | 700 | novel transcript |
| AC025034.1 | 7,11 | 0,00 | 5,87 | 12 | 89561129 | 89594878 | + | 1711 | novel transcript, antisense to ATP2B1 |
| AC012358.3 | 0,00 | 6,76 | -5,79 | 2 | 55282350 | 55346049 | + | 4741 | novel transcript, antisense to CCDC88A |
| LINC02340 | 0,00 | 6,76 | -5,79 | 13 | 27178263 | 27251288 | - | 1098 | long intergenic non-protein coding RNA 2340 [Source:HGNC Symbol;Acc:HGNC:53260] |
| ALOX12B | 7,11 | 0,00 | 5,87 | 17 | 8072636 | 8087716 | - | 2746 | arachidonate 12-lipoxygenase, 12R type [Source:HGNC Symbol;Acc:HGNC:430] |
| ETF1P2 | 7,11 | 0,00 | 5,87 | 7 | 151501878 | 151503463 | + | 1586 | eukaryotic translation termination factor 1 pseudogene 2 [Source:HGNC Symbol;Acc:HGNC:3479] |
| AL391839.2 | 7,11 | 0,00 | 5,87 | 10 | 32346499 | 32347179 | + | 383 | novel transcript, antisense to EPC1 |
| CAV3 | 0,00 | 6,76 | -5,79 | 3 | 8733800 | 8841808 | + | 1956 | caveolin 3 [Source:HGNC Symbol;Acc:HGNC:1529] |
| AC032044.1 | 7,11 | 0,00 | 5,87 | 17 | 1424473 | 1426484 | + | 691 | novel transcript, antisense to CRK |
| TCP1P1 | 7,11 | 0,00 | 5,87 | 7 | 42794906 | 42802494 | + | 1610 | t-complex 1 pseudogene 1 [Source:HGNC Symbol;Acc:HGNC:11659] |
| CHRNG | 0,00 | 6,76 | -5,79 | 2 | 232539727 | 232546403 | + | 2615 | cholinergic receptor nicotinic gamma subunit [Source:HGNC Symbol;Acc:HGNC:1967] |
| AC116036.2 | 7,11 | 0,00 | 5,87 | 3 | 58490830 | 58491291 | - | 462 | novel transcript |
| AL138921.1 | 0,00 | 6,76 | -5,79 | 10 | 100229667 | 100234000 | + | 846 | novel transcript |
| ANKRD36BP2 | 7,11 | 0,00 | 5,87 | 2 | 88765807 | 88806612 | + | 8042 | ankyrin repeat domain 36B pseudogene 2 [Source:HGNC Symbol;Acc:HGNC:33607] |
| LMF1-AS1 | 0,00 | 6,76 | -5,79 | 16 | 921033 | 934495 | + | 1448 | LMF1 antisense RNA 1 [Source:HGNC Symbol;Acc:HGNC:50469] |
| NOXO1 | 0,00 | 6,76 | -5,79 | 16 | 1978917 | 1984192 | - | 2159 | NADPH oxidase organizer 1 [Source:HGNC Symbol;Acc:HGNC:19404] |
| PNLIPRP3 | 7,11 | 0,00 | 5,87 | 10 | 116427867 | 116477957 | + | 2354 | pancreatic lipase related protein 3 [Source:HGNC Symbol;Acc:HGNC:23492] |
| PRR4 | 0,00 | 6,76 | -5,79 | 12 | 10845849 | 10849475 | - | 1310 | proline rich 4 [Source:HGNC Symbol;Acc:HGNC:18020] |
| HNRNPA1P14 | 7,11 | 0,00 | 5,87 | 9 | 88410529 | 88411463 | - | 935 | heterogeneous nuclear ribonucleoprotein A1 pseudogene 14 [Source:HGNC Symbol;Acc:HGNC:39132] |
| RN7SL351P | 7,11 | 0,00 | 5,87 | 11 | 126245035 | 126245282 | + | 248 | RNA, 7SL, cytoplasmic 351, pseudogene [Source:HGNC Symbol;Acc:HGNC:46367] |
| AL355075.5 | 7,11 | 0,00 | 5,87 | 14 | 20437982 | 20441268 | + | 992 | solute carrier family 12 (sodium/chloride transporters), member 3 (SLC12A3) pseudogene |
| AC138627.1 | 0,00 | 6,76 | -5,79 | 16 | 73943078 | 74296762 | - | 7473 | uncharacterized LOC101928035 [Source:NCBI gene;Acc:101928035] |
| RF01293 | 7,11 | 0,00 | 5,87 | 2 | 63883249 | 63883391 | - | 143 |  |
| AL359710.1 | 0,00 | 6,76 | -5,79 | 9 | 99585786 | 99819889 | - | 1544 | uncharacterized LOC101928438 [Source:NCBI gene;Acc:101928438] |
| AC026951.1 | 0,00 | 6,76 | -5,79 | 15 | 31765743 | 31768201 | - | 2459 | DEP domain containing 1 (DEPDC1) pseudogene |
| VN1R84P | 7,11 | 0,00 | 5,87 | 19 | 21719801 | 21720035 | - | 235 | vomeronasal 1 receptor 84 pseudogene [Source:HGNC Symbol;Acc:HGNC:37404] |
| CEACAM20 | 7,11 | 0,00 | 5,87 | 19 | 44501677 | 44529788 | - | 2193 | carcinoembryonic antigen related cell adhesion molecule 20 [Source:HGNC Symbol;Acc:HGNC:24879] |
| AC093817.1 | 7,11 | 0,00 | 5,87 | 4 | 157637687 | 157667044 | + | 812 | novel transcript |
| AC079465.1 | 7,11 | 0,00 | 5,87 | 5 | 113323028 | 113437174 | + | 4194 | novel transcript |
| AL355336.1 | 0,00 | 6,76 | -5,79 | 6 | 7183083 | 7185287 | + | 1676 | novel transcript |
| C9orf153 | 7,11 | 0,00 | 5,87 | 9 | 86220265 | 86259657 | - | 2928 | chromosome 9 open reading frame 153 [Source:HGNC Symbol;Acc:HGNC:31456] |
| AC073367.1 | 0,00 | 6,76 | -5,79 | 10 | 16721352 | 16748377 | - | 697 | novel transcript, sense intronic to RSU1 |
| AC105137.2 | 0,00 | 6,76 | -5,79 | 15 | 75452964 | 75453947 | - | 984 | novel transcript, sense intronic to SIN3A |
| SCARNA21 | 7,11 | 0,00 | 5,87 | 17 | 7906122 | 7906260 | + | 139 | small Cajal body-specific RNA 21 [Source:HGNC Symbol;Acc:HGNC:32579] |
| RNU2-63P | 0,00 | 6,76 | -5,79 | 2 | 88016354 | 88016547 | - | 194 | RNA, U2 small nuclear 63, pseudogene [Source:HGNC Symbol;Acc:HGNC:48556] |
| TIMM8AP1 | 0,00 | 6,76 | -5,79 | 2 | 162077357 | 162077651 | - | 295 | translocase of inner mitochondrial membrane 8A pseudogene 1 [Source:HGNC Symbol;Acc:HGNC:17802] |
| AC104088.1 | 0,00 | 6,76 | -5,79 | 2 | 172315287 | 172323737 | + | 510 | novel transcript |
| RN7SL711P | 0,00 | 6,76 | -5,79 | 5 | 123070782 | 123071080 | + | 299 | RNA, 7SL, cytoplasmic 711, pseudogene [Source:HGNC Symbol;Acc:HGNC:46727] |
| YBX1P4 | 7,11 | 0,00 | 5,87 | 7 | 152128879 | 152129686 | + | 808 | Y-box binding protein 1 pseudogene 4 [Source:HGNC Symbol;Acc:HGNC:42425] |
| AC023794.3 | 0,00 | 6,76 | -5,79 | 12 | 54076838 | 54081903 | - | 1390 | uncharacterized LOC100240735 [Source:NCBI gene;Acc:100240735] |
| MAF | 7,11 | 0,00 | 5,87 | 16 | 79585843 | 79600714 | - | 6976 | MAF bZIP transcription factor [Source:HGNC Symbol;Acc:HGNC:6776] |
| AC010260.1 | 7,11 | 0,00 | 5,87 | 5 | 80128361 | 80143883 | + | 664 | novel transcript |
| AC007666.1 | 7,11 | 0,00 | 5,87 | 22 | 17580157 | 17589192 | - | 1960 | novel transcript |
| UBXN10 | 0,00 | 6,76 | -5,79 | 1 | 20186085 | 20196048 | + | 5571 | UBX domain protein 10 [Source:HGNC Symbol;Acc:HGNC:26354] |
| OBP2A | 0,00 | 6,76 | -5,79 | 9 | 135546139 | 135549969 | + | 1022 | odorant binding protein 2A [Source:HGNC Symbol;Acc:HGNC:23380] |
| HRH4 | 0,00 | 6,76 | -5,79 | 18 | 24460629 | 24479957 | + | 3668 | histamine receptor H4 [Source:HGNC Symbol;Acc:HGNC:17383] |
| TMEM229B | 51,58 | 110,19 | -1,09 | 14 | 67447084 | 67533739 | - | 6377 | transmembrane protein 229B [Source:HGNC Symbol;Acc:HGNC:20130] |
| TBXA2R | 4,44 | 16,87 | -1,89 | 19 | 3594506 | 3606840 | - | 2793 | thromboxane A2 receptor [Source:HGNC Symbol;Acc:HGNC:11608] |
| HMCN1 | 16,89 | 3,38 | 2,28 | 1 | 185734551 | 186190949 | + | 18579 | hemicentin 1 [Source:HGNC Symbol;Acc:HGNC:19194] |
| AC008440.1 | 4,44 | 16,87 | -1,89 | 19 | 53854581 | 53869107 | - | 439 | novel transcript, antisense to MYADM |
| AMZ1 | 16,89 | 3,38 | 2,28 | 7 | 2679522 | 2775500 | + | 7011 | archaelysin family metallopeptidase 1 [Source:HGNC Symbol;Acc:HGNC:22231] |
| LINC00589 | 16,89 | 3,38 | 2,28 | 8 | 29673922 | 29748109 | - | 2074 | long intergenic non-protein coding RNA 589 [Source:HGNC Symbol;Acc:HGNC:32299] |
| CDH26 | 16,89 | 3,38 | 2,28 | 20 | 59958427 | 60034011 | + | 5748 | cadherin 26 [Source:HGNC Symbol;Acc:HGNC:15902] |
| XIRP1 | 16,89 | 3,38 | 2,28 | 3 | 39183210 | 39192596 | - | 6467 | xin actin binding repeat containing 1 [Source:HGNC Symbol;Acc:HGNC:14301] |
| SCN4B | 32,01 | 73,09 | -1,19 | 11 | 118133377 | 118152888 | - | 5649 | sodium voltage-gated channel beta subunit 4 [Source:HGNC Symbol;Acc:HGNC:10592] |
| MSRB3 | 70,26 | 30,37 | 1,21 | 12 | 65278643 | 65491430 | + | 7405 | methionine sulfoxide reductase B3 [Source:HGNC Symbol;Acc:HGNC:27375] |
| GNAI1 | 28,45 | 65,22 | -1,19 | 7 | 79768028 | 80226181 | + | 24206 | G protein subunit alpha i1 [Source:HGNC Symbol;Acc:HGNC:4384] |
| LINC00431 | 64,92 | 28,12 | 1,20 | 13 | 110965704 | 110990579 | + | 1189 | long intergenic non-protein coding RNA 431 [Source:HGNC Symbol;Acc:HGNC:42766] |
| ITGA9 | 80,04 | 165,28 | -1,04 | 3 | 37452115 | 37823514 | + | 8277 | integrin subunit alpha 9 [Source:HGNC Symbol;Acc:HGNC:6145] |
| SCARNA13 | 18,67 | 4,51 | 2,02 | 14 | 95533355 | 95533629 | - | 275 | small Cajal body-specific RNA 13 [Source:HGNC Symbol;Acc:HGNC:32570] |
| TMEM190 | 18,67 | 4,51 | 2,02 | 19 | 55376836 | 55378244 | + | 593 | transmembrane protein 190 [Source:HGNC Symbol;Acc:HGNC:29632] |
| IL6 | 58,69 | 24,75 | 1,24 | 7 | 22725884 | 22732002 | + | 2069 | interleukin 6 [Source:HGNC Symbol;Acc:HGNC:6018] |
| LINC01836 | 97,83 | 197,89 | -1,02 | 19 | 782755 | 785080 | + | 870 | long intergenic non-protein coding RNA 1836 [Source:HGNC Symbol;Acc:HGNC:52652] |
| GATA5 | 23,12 | 55,10 | -1,25 | 20 | 62463497 | 62475970 | - | 2595 | GATA binding protein 5 [Source:HGNC Symbol;Acc:HGNC:15802] |
| SLAMF7 | 20,45 | 5,63 | 1,84 | 1 | 160739057 | 160754821 | + | 4833 | SLAM family member 7 [Source:HGNC Symbol;Acc:HGNC:21394] |
| CTHRC1 | 20,45 | 5,63 | 1,84 | 8 | 103371515 | 103382997 | + | 1776 | collagen triple helix repeat containing 1 [Source:HGNC Symbol;Acc:HGNC:18831] |
| AL121772.1 | 20,45 | 5,63 | 1,84 | 20 | 25239007 | 25245229 | - | 2335 | novel transcript |
| TRABD2B | 47,13 | 19,12 | 1,30 | 1 | 47760528 | 47996895 | - | 7034 | TraB domain containing 2B [Source:HGNC Symbol;Acc:HGNC:44200] |
| ATP2C2-AS1 | 16,89 | 43,86 | -1,37 | 16 | 84459259 | 84467361 | - | 2998 | ATP2C2 antisense RNA 1 [Source:HGNC Symbol;Acc:HGNC:53167] |
| NYAP1 | 17,78 | 43,86 | -1,30 | 7 | 100483927 | 100494799 | + | 3586 | neuronal tyrosine phosphorylated phosphoinositide-3-kinase adaptor 1 [Source:HGNC Symbol;Acc:HGNC:22009] |
| SUGT1P4-STRA6LP | 72,03 | 31,49 | 1,19 | 9 | 97238449 | 97297314 | + | 3701 | SUGT1P4-STRA6LP readthrough [Source:HGNC Symbol;Acc:HGNC:53834] |
| GDAP1 | 113,84 | 53,98 | 1,08 | 8 | 74321130 | 74488872 | + | 4052 | ganglioside induced differentiation associated protein 1 [Source:HGNC Symbol;Acc:HGNC:15968] |
| LINC00702 | 23,12 | 6,76 | 1,76 | 10 | 4201141 | 4243912 | - | 6785 | long intergenic non-protein coding RNA 702 [Source:HGNC Symbol;Acc:HGNC:44676] |
| IL12A | 22,23 | 6,76 | 1,70 | 3 | 159988750 | 159996019 | + | 2040 | interleukin 12A [Source:HGNC Symbol;Acc:HGNC:5969] |
| SMG1P6 | 22,23 | 6,76 | 1,70 | 16 | 29425800 | 29447026 | - | 2930 | SMG1 pseudogene 6 [Source:HGNC Symbol;Acc:HGNC:49863] |
| AL023583.1 | 22,23 | 6,76 | 1,70 | 6 | 13825432 | 13826574 | + | 1143 | novel transcript |
| LENG8-AS1 | 69,37 | 30,37 | 1,19 | 19 | 54444813 | 54449045 | - | 1248 | LENG8 antisense RNA 1 [Source:HGNC Symbol;Acc:HGNC:40705] |
| AC022400.5 | 38,24 | 14,63 | 1,38 | 10 | 73703735 | 73713581 | - | 1192 | novel transcript |
| RAB7B | 109,39 | 51,73 | 1,08 | 1 | 205976740 | 206003461 | - | 3686 | RAB7B, member RAS oncogene family [Source:HGNC Symbol;Acc:HGNC:30513] |
| PRKAG3 | 36,46 | 13,50 | 1,43 | 2 | 218822383 | 218832086 | - | 3334 | protein kinase AMP-activated non-catalytic subunit gamma 3 [Source:HGNC Symbol;Acc:HGNC:9387] |
| AC011483.1 | 32,90 | 11,25 | 1,54 | 19 | 50968210 | 51012129 | + | 536 | novel transcript, antisense to KLK6, KLK7, KLK8 and KLK9 |
| LINC01529 | 28,45 | 10,13 | 1,48 | 19 | 35785697 | 35797902 | - | 4415 | long intergenic non-protein coding RNA 1529 [Source:HGNC Symbol;Acc:HGNC:51268] |
| C2CD4C | 10,66 | 29,24 | -1,44 | 19 | 405438 | 409170 | - | 3129 | C2 calcium dependent domain containing 4C [Source:HGNC Symbol;Acc:HGNC:29417] |
| SH3BGR | 10,66 | 29,24 | -1,44 | 21 | 39445855 | 39515506 | + | 1495 | SH3 domain binding glutamate rich protein [Source:HGNC Symbol;Acc:HGNC:10822] |
| TBC1D3G | 9,77 | 29,24 | -1,57 | 17 | 36323884 | 36334759 | + | 2065 | TBC1 domain family member 3G [Source:HGNC Symbol;Acc:HGNC:29860] |
| LINC00160 | 28,45 | 10,13 | 1,48 | 21 | 34723807 | 34737181 | - | 693 | long intergenic non-protein coding RNA 160 [Source:HGNC Symbol;Acc:HGNC:1294] |
| AC145343.1 | 11,55 | 2,26 | 2,30 | 17 | 68096046 | 68101474 | - | 650 | novel transcript |
| PIK3CD-AS2 | 2,66 | 12,38 | -2,16 | 1 | 9672426 | 9687555 | - | 939 | PIK3CD antisense RNA 2 [Source:HGNC Symbol;Acc:HGNC:51334] |
| AC087385.1 | 11,55 | 2,26 | 2,30 | 15 | 60390371 | 60390682 | + | 312 | ribosomal protein L36a (RPL36A) pseudogene |
| AP001001.1 | 1,77 | 12,38 | -2,71 | 11 | 106085990 | 106101975 | - | 1247 | novel transcript |
| RN7SKP173 | 11,55 | 2,26 | 2,30 | 20 | 38761528 | 38761842 | + | 315 | RNA, 7SK small nuclear pseudogene 173 [Source:HGNC Symbol;Acc:HGNC:45897] |
| AC003965.2 | 1,77 | 12,38 | -2,71 | 16 | 2857898 | 2859726 | + | 265 | novel transcript, antisense to PRSS22 |
| AC010201.2 | 2,66 | 12,38 | -2,16 | 12 | 89367807 | 89369301 | + | 1495 | novel transcript |
| AC055855.1 | 1,77 | 12,38 | -2,71 | 15 | 66314914 | 66331703 | - | 500 | novel transcript, antisense to DIS3L |
| PKD2L2 | 2,66 | 12,38 | -2,16 | 5 | 137887968 | 137942747 | + | 4653 | polycystin 2 like 2, transient receptor potential cation channel [Source:HGNC Symbol;Acc:HGNC:9012] |
| AC079148.1 | 1,77 | 12,38 | -2,71 | 2 | 18547386 | 18548204 | - | 819 | novel transcript |
| AC016542.1 | 2,66 | 12,38 | -2,16 | 10 | 72766560 | 72767052 | + | 493 | novel transcript, sense intronic with MCU |
| SLC25A41 | 2,66 | 12,38 | -2,16 | 19 | 6426037 | 6433779 | - | 1649 | solute carrier family 25 member 41 [Source:HGNC Symbol;Acc:HGNC:28533] |
| LINC01399 | 11,55 | 2,26 | 2,30 | 22 | 35119824 | 35231056 | - | 806 | long intergenic non-protein coding RNA 1399 [Source:HGNC Symbol;Acc:HGNC:50680] |
| KCNJ4 | 2,66 | 12,38 | -2,16 | 22 | 38426327 | 38455199 | - | 2065 | potassium voltage-gated channel subfamily J member 4 [Source:HGNC Symbol;Acc:HGNC:6265] |
| AP003498.2 | 2,66 | 12,38 | -2,16 | 11 | 71745331 | 71747051 | + | 1145 | novel transcript |
| TWIST1 | 11,55 | 2,26 | 2,30 | 7 | 19020991 | 19117672 | - | 2033 | twist family bHLH transcription factor 1 [Source:HGNC Symbol;Acc:HGNC:12428] |
| AC020765.3 | 1,77 | 12,38 | -2,71 | 16 | 28599241 | 28601881 | - | 740 | SLX1 structure-specific endonuclease subunit homolog A (S. cerevisiae) (SLX1A) pseudogene |
| AL022328.4 | 26,67 | 60,72 | -1,18 | 22 | 50199090 | 50200837 | - | 1001 | novel transcript, antisense TRABD |
| LMOD1 | 25,78 | 58,47 | -1,18 | 1 | 201896452 | 201946588 | - | 3971 | leiomodin 1 [Source:HGNC Symbol;Acc:HGNC:6647] |
| CHRNA10 | 24,01 | 56,23 | -1,22 | 11 | 3665587 | 3671384 | - | 2703 | cholinergic receptor nicotinic alpha 10 subunit [Source:HGNC Symbol;Acc:HGNC:13800] |
| CD164L2 | 24,01 | 56,23 | -1,22 | 1 | 27379176 | 27383380 | - | 2041 | CD164 molecule like 2 [Source:HGNC Symbol;Acc:HGNC:32043] |
| SEMA4G | 74,70 | 33,74 | 1,14 | 10 | 100969518 | 100985871 | + | 5253 | semaphorin 4G [Source:HGNC Symbol;Acc:HGNC:10735] |
| ELL3 | 33,79 | 73,09 | -1,11 | 15 | 43772600 | 43777543 | - | 2880 | elongation factor for RNA polymerase II 3 [Source:HGNC Symbol;Acc:HGNC:23113] |
| HIST1H2BN | 41,79 | 88,83 | -1,09 | 6 | 27837760 | 27865798 | + | 5477 | histone cluster 1 H2B family member n [Source:HGNC Symbol;Acc:HGNC:4749] |
| PNPLA7 | 88,04 | 40,49 | 1,12 | 9 | 137459953 | 137550534 | - | 9976 | patatin like phospholipase domain containing 7 [Source:HGNC Symbol;Acc:HGNC:24768] |
| IL10RB | 21,34 | 50,60 | -1,24 | 21 | 33266358 | 33310187 | + | 4548 | interleukin 10 receptor subunit beta [Source:HGNC Symbol;Acc:HGNC:5965] |
| SUSD5 | 68,48 | 30,37 | 1,17 | 3 | 33150042 | 33219215 | - | 5008 | sushi domain containing 5 [Source:HGNC Symbol;Acc:HGNC:29061] |
| TRPV3 | 80,04 | 37,11 | 1,11 | 17 | 3510502 | 3557995 | - | 9780 | transient receptor potential cation channel subfamily V member 3 [Source:HGNC Symbol;Acc:HGNC:18084] |
| CAND2 | 3,55 | 14,63 | -2,00 | 3 | 12796472 | 12871916 | + | 5587 | cullin associated and neddylation dissociated 2 (putative) [Source:HGNC Symbol;Acc:HGNC:30689] |
| RN7SL5P | 3,55 | 14,63 | -2,00 | 9 | 9442060 | 9442380 | + | 321 | RNA, 7SL, cytoplasmic 5, pseudogene [Source:HGNC Symbol;Acc:HGNC:10040] |
| AC008494.3 | 3,55 | 14,63 | -2,00 | 5 | 115262505 | 115263448 | + | 944 | novel transcript, antisense to PGGT1B |
| AC004890.2 | 137,85 | 67,47 | 1,03 | 7 | 149285281 | 149297312 | + | 4288 | AI894139 pseudogene [Source:NCBI gene;Acc:155060] |
| LINP1 | 62,25 | 28,12 | 1,14 | 10 | 6709530 | 6740532 | + | 4118 | lncRNA in non-homologous end joining pathway 1 [Source:HGNC Symbol;Acc:HGNC:53170] |
| SNAI3 | 36,46 | 78,71 | -1,11 | 16 | 88677682 | 88686493 | - | 1732 | snail family transcriptional repressor 3 [Source:HGNC Symbol;Acc:HGNC:18411] |
| AK5 | 44,46 | 93,33 | -1,07 | 1 | 77282051 | 77559969 | + | 6919 | adenylate kinase 5 [Source:HGNC Symbol;Acc:HGNC:365] |
| AC079250.1 | 16,00 | 40,49 | -1,33 | 2 | 47690716 | 47691246 | - | 531 | ribosomal protein L18a (RPL18A) pseudogene |
| KCNJ5 | 16,00 | 40,49 | -1,33 | 11 | 128891356 | 128921035 | + | 6109 | potassium voltage-gated channel subfamily J member 5 [Source:HGNC Symbol;Acc:HGNC:6266] |
| LRMDA | 16,00 | 40,49 | -1,33 | 10 | 75431453 | 76560167 | + | 6560 | leucine rich melanocyte differentiation associated [Source:HGNC Symbol;Acc:HGNC:23405] |
| FFAR1 | 16,00 | 40,49 | -1,33 | 19 | 35351552 | 35353862 | + | 2311 | free fatty acid receptor 1 [Source:HGNC Symbol;Acc:HGNC:4498] |
| COL21A1 | 37,35 | 14,63 | 1,35 | 6 | 56056590 | 56394094 | - | 7078 | collagen type XXI alpha 1 chain [Source:HGNC Symbol;Acc:HGNC:17025] |
| GRIK5 | 35,57 | 13,50 | 1,39 | 19 | 41998321 | 42069498 | - | 5014 | glutamate ionotropic receptor kainate type subunit 5 [Source:HGNC Symbol;Acc:HGNC:4583] |
| AL731684.1 | 35,57 | 13,50 | 1,39 | 6 | 170736173 | 170737777 | - | 439 | novel transcript |
| RN7SKP80 | 16,00 | 3,38 | 2,21 | 22 | 42565048 | 42565330 | - | 283 | RNA, 7SK small nuclear pseudogene 80 [Source:HGNC Symbol;Acc:HGNC:45804] |
| CYBB | 16,00 | 3,38 | 2,21 | X | 37780011 | 37813461 | + | 4423 | cytochrome b-245 beta chain [Source:HGNC Symbol;Acc:HGNC:2578] |
| FYB1 | 16,00 | 3,38 | 2,21 | 5 | 39105255 | 39274528 | - | 8850 | FYN binding protein 1 [Source:HGNC Symbol;Acc:HGNC:4036] |
| AC138207.8 | 33,79 | 12,38 | 1,44 | 17 | 31008497 | 31093127 | + | 2147 | SMAD specific E3 ubiquitin protein ligase 2 (SMURF2) pseudogene |
| C11orf53 | 31,12 | 68,59 | -1,14 | 11 | 111245805 | 111286401 | + | 1264 | chromosome 11 open reading frame 53 [Source:HGNC Symbol;Acc:HGNC:30527] |
| LIPK | 50,69 | 21,37 | 1,24 | 10 | 88724544 | 88752786 | + | 1230 | lipase family member K [Source:HGNC Symbol;Acc:HGNC:23444] |
| MUM1L1 | 65,81 | 29,24 | 1,17 | X | 106168305 | 106208956 | + | 4343 | MUM1 like 1 [Source:HGNC Symbol;Acc:HGNC:26583] |
| AL606760.1 | 11,55 | 30,37 | -1,38 | 1 | 53238610 | 53242783 | + | 1181 | uncharacterized LOC100507564 [Source:NCBI gene;Acc:100507564] |
| AC110285.7 | 51,58 | 105,70 | -1,03 | 17 | 81395609 | 81397144 | - | 1536 | TEC |
| CDHR3 | 17,78 | 4,51 | 1,95 | 7 | 105876796 | 106033773 | + | 7087 | cadherin related family member 3 [Source:HGNC Symbol;Acc:HGNC:26308] |
| FFAR4 | 17,78 | 4,51 | 1,95 | 10 | 93566665 | 93604480 | + | 4060 | free fatty acid receptor 4 [Source:HGNC Symbol;Acc:HGNC:19061] |
| KRTAP2-3 | 17,78 | 4,51 | 1,95 | 17 | 41059240 | 41060114 | - | 875 | keratin associated protein 2-3 [Source:HGNC Symbol;Acc:HGNC:18906] |
| MT-TV | 17,78 | 4,51 | 1,95 | MT | 1602 | 1670 | + | 69 | mitochondrially encoded tRNA valine [Source:HGNC Symbol;Acc:HGNC:7500] |
| MT-TR | 17,78 | 4,51 | 1,95 | MT | 10405 | 10469 | + | 65 | mitochondrially encoded tRNA arginine [Source:HGNC Symbol;Acc:HGNC:7496] |
| ZNF154 | 5,33 | 18,00 | -1,73 | 19 | 57697367 | 57709194 | - | 5713 | zinc finger protein 154 [Source:HGNC Symbol;Acc:HGNC:12939] |
| CRB2 | 5,33 | 18,00 | -1,73 | 9 | 123356170 | 123380324 | + | 6349 | crumbs 2, cell polarity complex component [Source:HGNC Symbol;Acc:HGNC:18688] |
| LINC01732 | 17,78 | 4,51 | 1,95 | 1 | 181174484 | 181182208 | + | 667 | long intergenic non-protein coding RNA 1732 [Source:HGNC Symbol;Acc:HGNC:52520] |
| OGFR-AS1 | 10,66 | 28,12 | -1,39 | 20 | 62800627 | 62805587 | - | 668 | OGFR antisense RNA 1 [Source:HGNC Symbol;Acc:HGNC:40724] |
| AURKC | 29,34 | 64,10 | -1,12 | 19 | 57230802 | 57235548 | + | 1948 | aurora kinase C [Source:HGNC Symbol;Acc:HGNC:11391] |
| AP001148.1 | 8,88 | 25,87 | -1,53 | 11 | 86434924 | 86437282 | - | 2359 | novel transcript |
| AL391988.1 | 8,88 | 25,87 | -1,53 | 10 | 117267116 | 117268668 | - | 1553 | novel transcript, antisense to SLC18A2 |
| CDNF | 8,88 | 25,87 | -1,53 | 10 | 14819250 | 14838575 | - | 1733 | cerebral dopamine neurotrophic factor [Source:HGNC Symbol;Acc:HGNC:24913] |
| PSPC1-AS2 | 25,78 | 9,00 | 1,51 | 13 | 19674624 | 19675884 | + | 376 | PSPC1 antisense RNA 2 [Source:HGNC Symbol;Acc:HGNC:52951] |
| DMRTA1 | 25,78 | 9,00 | 1,51 | 9 | 22446841 | 22455740 | + | 5569 | DMRT like family A1 [Source:HGNC Symbol;Acc:HGNC:13826] |
| AC003070.1 | 19,56 | 5,63 | 1,78 | 17 | 45396932 | 45397477 | + | 457 | novel transcript, antisense to ARHGAP27 |
| AC005722.2 | 19,56 | 5,63 | 1,78 | 17 | 19719059 | 19722428 | + | 3370 | novel transcript |
| CALHM3 | 24,01 | 7,88 | 1,59 | 10 | 103472804 | 103479240 | - | 1652 | calcium homeostasis modulator 3 [Source:HGNC Symbol;Acc:HGNC:23458] |
| LINC00921 | 21,34 | 6,76 | 1,64 | 16 | 3263743 | 3267567 | + | 2468 | long intergenic non-protein coding RNA 921 [Source:HGNC Symbol;Acc:HGNC:26830] |
| AC096537.1 | 8,88 | 1,13 | 2,85 | 1 | 224611404 | 224616220 | - | 684 | novel transcript |
| RIMBP3 | 8,88 | 1,13 | 2,85 | 22 | 18605815 | 18611919 | - | 6105 | RIMS binding protein 3 [Source:HGNC Symbol;Acc:HGNC:29344] |
| LINC01909 | 8,88 | 1,13 | 2,85 | 18 | 70335439 | 70352459 | + | 1040 | long intergenic non-protein coding RNA 1909 [Source:HGNC Symbol;Acc:HGNC:52728] |
| RNA5SP317 | 8,88 | 1,13 | 2,85 | 10 | 49979665 | 49979780 | - | 116 | RNA, 5S ribosomal pseudogene 317 [Source:HGNC Symbol;Acc:HGNC:43217] |
| FOXA3 | 0,88 | 9,00 | -3,17 | 19 | 45863989 | 45873797 | + | 2192 | forkhead box A3 [Source:HGNC Symbol;Acc:HGNC:5023] |
| RNASEH2B-AS1 | 0,88 | 9,00 | -3,17 | 13 | 50862172 | 50910764 | - | 4642 | RNASEH2B antisense RNA 1 [Source:HGNC Symbol;Acc:HGNC:39967] |
| OR7E38P | 8,88 | 1,13 | 2,85 | 7 | 97966090 | 97967074 | - | 985 | olfactory receptor family 7 subfamily E member 38 pseudogene [Source:HGNC Symbol;Acc:HGNC:8411] |
| AC131097.1 | 8,88 | 1,13 | 2,85 | 2 | 241800916 | 241801907 | - | 739 | novel transcript |
| FAM187B2P | 8,88 | 1,13 | 2,85 | 19 | 35232291 | 35233003 | - | 713 | family with sequence similarity 187 member B2, pseudogene [Source:HGNC Symbol;Acc:HGNC:49213] |
| AL138752.2 | 8,88 | 1,13 | 2,85 | 9 | 37588413 | 38068687 | - | 2591 | novel transcript |
| AC023043.3 | 8,88 | 1,13 | 2,85 | 18 | 36278735 | 36279119 | - | 385 | related RAS viral (r-ras) oncogene homolog 2, pseudogene |
| AC018797.3 | 0,88 | 9,00 | -3,17 | 4 | 102751401 | 102752641 | + | 1241 | WD repeat domain 12 (WDR12) pseudogene |
| GATA3-AS1 | 8,88 | 1,13 | 2,85 | 10 | 8050450 | 8053484 | - | 2372 | GATA3 antisense RNA 1 [Source:HGNC Symbol;Acc:HGNC:33786] |
| AC011442.1 | 8,88 | 1,13 | 2,85 | 19 | 11010917 | 11016011 | - | 366 | novel transcript, antisense to SMARCA4 |
| BBIP1P1 | 0,88 | 9,00 | -3,17 | 2 | 101068378 | 101068656 | + | 279 | BBSome interacting protein 1 pseudogene 1 [Source:HGNC Symbol;Acc:HGNC:52781] |
| AC005154.4 | 0,88 | 9,00 | -3,17 | 7 | 30525731 | 30533613 | + | 368 | novel transcript |
| AL355490.1 | 0,88 | 9,00 | -3,17 | 10 | 97334564 | 97343203 | + | 805 | novel transcript |
| AC104695.1 | 8,88 | 1,13 | 2,85 | 2 | 28448167 | 28450184 | - | 849 | novel transcript |
| CEACAM22P | 8,88 | 1,13 | 2,85 | 19 | 44537058 | 44620821 | - | 3170 | carcinoembryonic antigen related cell adhesion molecule 22, pseudogene [Source:HGNC Symbol;Acc:HGNC:38029] |
| CFAP65 | 8,88 | 1,13 | 2,85 | 2 | 219002846 | 219041527 | - | 10519 | cilia and flagella associated protein 65 [Source:HGNC Symbol;Acc:HGNC:25325] |
| NYX | 8,88 | 1,13 | 2,85 | X | 41447434 | 41475710 | + | 2988 | nyctalopin [Source:HGNC Symbol;Acc:HGNC:8082] |
| SLX1A-SULT1A3 | 0,88 | 9,00 | -3,17 | 16 | 30193892 | 30204310 | + | 4611 | SLX1A-SULT1A3 readthrough (NMD candidate) [Source:HGNC Symbol;Acc:HGNC:44437] |
| CD40LG | 0,88 | 9,00 | -3,17 | X | 136648193 | 136660390 | + | 1817 | CD40 ligand [Source:HGNC Symbol;Acc:HGNC:11935] |
| PCDH12 | 26,67 | 58,47 | -1,13 | 5 | 141943585 | 141969741 | - | 6820 | protocadherin 12 [Source:HGNC Symbol;Acc:HGNC:8657] |
| BRWD1-AS2 | 26,67 | 58,47 | -1,13 | 21 | 39313935 | 39314962 | + | 1028 | BRWD1 antisense RNA 2 [Source:HGNC Symbol;Acc:HGNC:16423] |
| AC083880.1 | 18,67 | 43,86 | -1,23 | 7 | 139359032 | 139359566 | - | 535 | novel transcript, antisense to LUC7L2 |
| CSTA | 68,48 | 31,49 | 1,12 | 3 | 122325244 | 122341972 | + | 1373 | cystatin A [Source:HGNC Symbol;Acc:HGNC:2481] |
| RIPPLY3 | 16,89 | 41,61 | -1,29 | 21 | 37006150 | 37019659 | + | 2319 | ripply transcriptional repressor 3 [Source:HGNC Symbol;Acc:HGNC:3047] |
| PLA2G2F | 66,70 | 30,37 | 1,13 | 1 | 20139326 | 20150386 | + | 6440 | phospholipase A2 group IIF [Source:HGNC Symbol;Acc:HGNC:30040] |
| TMPRSS9 | 88,93 | 42,73 | 1,06 | 19 | 2360238 | 2426239 | + | 4328 | transmembrane serine protease 9 [Source:HGNC Symbol;Acc:HGNC:30079] |
| AC008915.2 | 16,00 | 39,36 | -1,29 | 16 | 23452758 | 23457606 | + | 1007 | novel transcript |
| SNORD101 | 38,24 | 15,75 | 1,27 | 6 | 132815307 | 132815379 | + | 73 | small nucleolar RNA, C/D box 101 [Source:HGNC Symbol;Acc:HGNC:32764] |
| AC010422.6 | 38,24 | 15,75 | 1,27 | 19 | 12525720 | 12580975 | - | 5857 | novel transcript |
| AL021997.2 | 39,13 | 15,75 | 1,31 | 6 | 28315613 | 28315883 | - | 271 | zinc finger and SCAN domain containing 16 (ZSCAN16) pseudogene |
| SOCS2 | 64,92 | 29,24 | 1,15 | 12 | 93569814 | 93583487 | + | 6776 | suppressor of cytokine signaling 2 [Source:HGNC Symbol;Acc:HGNC:19382] |
| AL133406.2 | 51,58 | 22,50 | 1,19 | 6 | 105279016 | 105281755 | + | 1198 | novel transcript |
| AC121764.1 | 51,58 | 22,50 | 1,19 | 3 | 55493830 | 55505261 | - | 563 | novel transcript |
| MSI1 | 35,57 | 74,21 | -1,06 | 12 | 120341330 | 120369180 | - | 3090 | musashi RNA binding protein 1 [Source:HGNC Symbol;Acc:HGNC:7330] |
| TRIM74 | 14,22 | 34,86 | -1,28 | 7 | 72959485 | 72969466 | - | 1471 | tripartite motif containing 74 [Source:HGNC Symbol;Acc:HGNC:17453] |
| AC017116.2 | 14,22 | 34,86 | -1,28 | 7 | 44011882 | 44038978 | - | 2698 | novel transcript |
| FLRT1 | 34,68 | 71,97 | -1,05 | 11 | 64103188 | 64119173 | + | 3949 | fibronectin leucine rich transmembrane protein 1 [Source:HGNC Symbol;Acc:HGNC:3760] |
| AC020917.4 | 48,02 | 20,25 | 1,24 | 19 | 16356329 | 16358327 | - | 1999 | TEC |
| C5AR1 | 48,02 | 20,25 | 1,24 | 19 | 47290023 | 47322066 | + | 2951 | complement C5a receptor 1 [Source:HGNC Symbol;Acc:HGNC:1338] |
| SNX15 | 58,69 | 26,99 | 1,12 | 11 | 65027408 | 65040572 | + | 4915 | sorting nexin 15 [Source:HGNC Symbol;Acc:HGNC:14978] |
| DUBR | 89,82 | 43,86 | 1,03 | 3 | 107240692 | 107326964 | + | 4206 | DPPA2 upstream binding RNA [Source:HGNC Symbol;Acc:HGNC:48569] |
| JMJD1C-AS1 | 20,45 | 46,11 | -1,17 | 10 | 63465229 | 63466563 | + | 1335 | JMJD1C antisense RNA 1 [Source:HGNC Symbol;Acc:HGNC:28222] |
| PDZK1P1 | 67,59 | 31,49 | 1,10 | 1 | 147993862 | 148014956 | - | 3674 | PDZ domain containing 1 pseudogene 1 [Source:HGNC Symbol;Acc:HGNC:31974] |
| PLXNA4 | 31,12 | 11,25 | 1,46 | 7 | 132123332 | 132648688 | - | 16372 | plexin A4 [Source:HGNC Symbol;Acc:HGNC:9102] |
| PSG6 | 30,23 | 11,25 | 1,42 | 19 | 42902079 | 42919563 | - | 5140 | pregnancy specific beta-1-glycoprotein 6 [Source:HGNC Symbol;Acc:HGNC:9523] |
| LINC00365 | 30,23 | 11,25 | 1,42 | 13 | 30103178 | 30108875 | - | 2998 | long intergenic non-protein coding RNA 365 [Source:HGNC Symbol;Acc:HGNC:42687] |
| SLC4A4 | 85,38 | 41,61 | 1,04 | 4 | 71062667 | 71572087 | + | 9622 | solute carrier family 4 member 4 [Source:HGNC Symbol;Acc:HGNC:11030] |
| HCG27 | 43,57 | 19,12 | 1,18 | 6 | 31197760 | 31203968 | + | 2592 | HLA complex group 27 [Source:HGNC Symbol;Acc:HGNC:27366] |
| PLAG1 | 6,22 | 0,00 | 5,68 | 8 | 56160904 | 56211324 | - | 7833 | PLAG1 zinc finger [Source:HGNC Symbol;Acc:HGNC:9045] |
| CTF1 | 6,22 | 0,00 | 5,68 | 16 | 30896607 | 30903560 | + | 1664 | cardiotrophin 1 [Source:HGNC Symbol;Acc:HGNC:2499] |
| IL1B | 6,22 | 0,00 | 5,68 | 2 | 112829751 | 112836903 | - | 3096 | interleukin 1 beta [Source:HGNC Symbol;Acc:HGNC:5992] |
| LRRN4 | 6,22 | 0,00 | 5,68 | 20 | 6040778 | 6054049 | - | 2692 | leucine rich repeat neuronal 4 [Source:HGNC Symbol;Acc:HGNC:16208] |
| MARCH4 | 6,22 | 0,00 | 5,68 | 2 | 216257865 | 216372027 | - | 4447 | membrane associated ring-CH-type finger 4 [Source:HGNC Symbol;Acc:HGNC:29269] |
| SCHIP1 | 6,22 | 0,00 | 5,68 | 3 | 159839861 | 159897360 | + | 7645 | schwannomin interacting protein 1 [Source:HGNC Symbol;Acc:HGNC:15678] |
| NEXN-AS1 | 6,22 | 0,00 | 5,68 | 1 | 77881348 | 77889539 | - | 2292 | NEXN antisense RNA 1 [Source:HGNC Symbol;Acc:HGNC:31983] |
| AL161729.1 | 6,22 | 0,00 | 5,68 | 9 | 95506235 | 95507636 | + | 1402 | novel transcript, antisense to PTCH1 |
| CLIP1-AS1 | 6,22 | 0,00 | 5,68 | 12 | 122395542 | 122400857 | + | 1809 | CLIP1 antisense RNA 1 [Source:HGNC Symbol;Acc:HGNC:48586] |
| AL022323.4 | 6,22 | 0,00 | 5,68 | 22 | 25052122 | 25065241 | - | 13120 | novel transcript |
| AL139021.1 | 6,22 | 0,00 | 5,68 | 14 | 58398557 | 58427125 | + | 436 | novel transcript, antisense to TIMM9 |
| LINC01968 | 6,22 | 0,00 | 5,68 | 3 | 194708421 | 194782168 | + | 4181 | long intergenic non-protein coding RNA 1968 [Source:HGNC Symbol;Acc:HGNC:52794] |
| MT-TW | 6,22 | 0,00 | 5,68 | MT | 5512 | 5579 | + | 68 | mitochondrially encoded tRNA tryptophan [Source:HGNC Symbol;Acc:HGNC:7501] |
| LINC00622 | 6,22 | 0,00 | 5,68 | 1 | 119597702 | 119599271 | - | 1570 | long intergenic non-protein coding RNA 622 [Source:HGNC Symbol;Acc:HGNC:44251] |
| LNX1-AS2 | 6,22 | 0,00 | 5,68 | 4 | 53592956 | 53604047 | + | 887 | LNX1 antisense RNA 2 [Source:HGNC Symbol;Acc:HGNC:41450] |
| AC004895.1 | 6,22 | 0,00 | 5,68 | 7 | 6081103 | 6093085 | + | 820 | novel transcript |
| RPL37P6 | 6,22 | 0,00 | 5,68 | 8 | 56588385 | 56588787 | + | 403 | ribosomal protein L37 pseudogene 6 [Source:HGNC Symbol;Acc:HGNC:31080] |
| RF00411 | 6,22 | 0,00 | 5,68 | 12 | 122492113 | 122492241 | - | 129 |  |
| LGALS9C | 6,22 | 0,00 | 5,68 | 17 | 18476737 | 18494945 | + | 4754 | galectin 9C [Source:HGNC Symbol;Acc:HGNC:33874] |
| AC024361.2 | 6,22 | 0,00 | 5,68 | 17 | 82713908 | 82716255 | - | 898 | uncharacterized LOC101929552 [Source:NCBI gene;Acc:101929552] |
| TRBV30 | 6,22 | 0,00 | 5,68 | 7 | 142812586 | 142813399 | - | 447 | T cell receptor beta variable 30 (gene/pseudogene) [Source:HGNC Symbol;Acc:HGNC:12214] |
| ABCB10P1 | 6,22 | 0,00 | 5,68 | 15 | 23179869 | 23182049 | + | 2181 | ABCB10 pseudogene 1 [Source:HGNC Symbol;Acc:HGNC:14114] |
| AL050343.2 | 6,22 | 0,00 | 5,68 | 1 | 51801028 | 51801307 | + | 280 | novel transcript, antisense to NRD1 |
| IL20 | 6,22 | 0,00 | 5,68 | 1 | 206865354 | 206869223 | + | 1567 | interleukin 20 [Source:HGNC Symbol;Acc:HGNC:6002] |
| AC037487.3 | 6,22 | 0,00 | 5,68 | 17 | 64951498 | 64951609 | - | 112 | eyes shut homolog (Drosophila) 9 (EYS) pseudogene |
| AL391845.2 | 6,22 | 0,00 | 5,68 | 1 | 2013213 | 2015530 | - | 1954 | novel transcript |
| LINC00458 | 6,22 | 0,00 | 5,68 | 13 | 54115783 | 54132866 | - | 2444 | long intergenic non-protein coding RNA 458 [Source:HGNC Symbol;Acc:HGNC:42807] |
| GAST | 6,22 | 0,00 | 5,68 | 17 | 41712326 | 41715969 | + | 470 | gastrin [Source:HGNC Symbol;Acc:HGNC:4164] |
| AC138904.3 | 6,22 | 0,00 | 5,68 | 16 | 28258686 | 28292173 | + | 2681 | novel transcript |
| AC005695.3 | 6,22 | 0,00 | 5,68 | 17 | 9244666 | 9244897 | - | 232 | novel transcript |
| KRTAP3-2 | 6,22 | 0,00 | 5,68 | 17 | 40999193 | 40999894 | - | 702 | keratin associated protein 3-2 [Source:HGNC Symbol;Acc:HGNC:16779] |
| IL17REL | 6,22 | 0,00 | 5,68 | 22 | 49994513 | 50012659 | - | 3696 | interleukin 17 receptor E like [Source:HGNC Symbol;Acc:HGNC:33808] |
| AL590556.1 | 6,22 | 0,00 | 5,68 | 1 | 21908098 | 21908580 | + | 483 | ribosomal protein L21 (RPL21) pseudogene |
| MIR135B | 6,22 | 0,00 | 5,68 | 1 | 205448302 | 205448398 | - | 97 | microRNA 135b [Source:HGNC Symbol;Acc:HGNC:31760] |
| UGT3A2 | 6,22 | 0,00 | 5,68 | 5 | 36035017 | 36071358 | - | 2694 | UDP glycosyltransferase family 3 member A2 [Source:HGNC Symbol;Acc:HGNC:27266] |
| MIR4512 | 6,22 | 0,00 | 5,68 | 15 | 66496958 | 66497034 | - | 77 | microRNA 4512 [Source:HGNC Symbol;Acc:HGNC:41701] |
| Z92544.1 | 6,22 | 0,00 | 5,68 | 16 | 678504 | 679777 | - | 1274 | uncharacterized LOC105371184 [Source:NCBI gene;Acc:105371184] |
| AC011471.4 | 6,22 | 0,00 | 5,68 | 19 | 6199405 | 6205121 | + | 216 | acyl-CoA synthetase bubblegum family member 2 (ACSBG2) pseudogene |
| AC004832.1 | 6,22 | 0,00 | 5,68 | 22 | 30475364 | 30492804 | + | 2422 | uncharacterized LOC107985579 [Source:NCBI gene;Acc:107985579] |
| AC004076.1 | 6,22 | 0,00 | 5,68 | 19 | 57437970 | 57477536 | - | 1028 | novel transcript |
| CACNA1E | 6,22 | 0,00 | 5,68 | 1 | 181317690 | 181808084 | + | 18271 | calcium voltage-gated channel subunit alpha1 E [Source:HGNC Symbol;Acc:HGNC:1392] |
| LINC02487 | 6,22 | 0,00 | 5,68 | 6 | 167679626 | 167696290 | - | 3867 | long intergenic non-protein coding RNA 2487 [Source:HGNC Symbol;Acc:HGNC:53466] |
| SMILR | 6,22 | 0,00 | 5,68 | 8 | 122414332 | 122428551 | - | 553 | smooth muscle induced lncRNA, enhancer of proliferation [Source:HGNC Symbol;Acc:HGNC:51825] |
| AC106779.1 | 6,22 | 0,00 | 5,68 | 16 | 56708772 | 56729968 | - | 1201 | uncharacterized LOC105371286 [Source:NCBI gene;Acc:105371286] |
| DMRTC2 | 6,22 | 0,00 | 5,68 | 19 | 41844743 | 41852333 | + | 3389 | DMRT like family C2 [Source:HGNC Symbol;Acc:HGNC:13911] |
| AC012146.3 | 6,22 | 0,00 | 5,68 | 17 | 5075678 | 5078113 | - | 490 | novel transcript, antisense to ZFP3 |
| GSDMA | 6,22 | 0,00 | 5,68 | 17 | 39953263 | 39977766 | + | 2273 | gasdermin A [Source:HGNC Symbol;Acc:HGNC:13311] |
| AC013457.1 | 6,22 | 0,00 | 5,68 | 2 | 118949306 | 118952983 | - | 1011 | novel transcript, antisense to MARCO |
| SPANXD | 6,22 | 0,00 | 5,68 | X | 141697411 | 141698739 | - | 682 | SPANX family member D [Source:HGNC Symbol;Acc:HGNC:14332] |
| AL158211.1 | 6,22 | 0,00 | 5,68 | 10 | 22257786 | 22258548 | + | 763 | novel transcript |
| HMGB1P50 | 6,22 | 0,00 | 5,68 | 10 | 49551308 | 49552652 | + | 607 | high mobility group box 1 pseudogene 50 [Source:HGNC Symbol;Acc:HGNC:52368] |
| AC089998.4 | 6,22 | 0,00 | 5,68 | 12 | 82481118 | 82497560 | - | 459 | novel transcript |
| RN7SL745P | 6,22 | 0,00 | 5,68 | 18 | 23004564 | 23004862 | + | 299 | RNA, 7SL, cytoplasmic 745, pseudogene [Source:HGNC Symbol;Acc:HGNC:46761] |
| NEBL-AS1 | 11,55 | 29,24 | -1,33 | 10 | 21174014 | 21175048 | + | 681 | NEBL antisense RNA 1 [Source:HGNC Symbol;Acc:HGNC:44899] |
| DNALI1 | 10,66 | 2,26 | 2,19 | 1 | 37556919 | 37566857 | + | 4456 | dynein axonemal light intermediate chain 1 [Source:HGNC Symbol;Acc:HGNC:14353] |
| CYP11A1 | 1,77 | 11,25 | -2,58 | 15 | 74337759 | 74367740 | - | 3491 | cytochrome P450 family 11 subfamily A member 1 [Source:HGNC Symbol;Acc:HGNC:2590] |
| EFHB | 1,77 | 11,25 | -2,58 | 3 | 19879472 | 19947025 | - | 4163 | EF-hand domain family member B [Source:HGNC Symbol;Acc:HGNC:26330] |
| AC099778.1 | 10,66 | 2,26 | 2,19 | 3 | 47379089 | 47380999 | - | 1911 | novel transcript, antisense to PTPN23 |
| TMEM220 | 1,77 | 11,25 | -2,58 | 17 | 10699015 | 10730316 | - | 3508 | transmembrane protein 220 [Source:HGNC Symbol;Acc:HGNC:33757] |
| AC012442.1 | 1,77 | 11,25 | -2,58 | 2 | 112590796 | 112591939 | + | 529 | novel transcript |
| AC092757.2 | 1,77 | 11,25 | -2,58 | 15 | 59121034 | 59133250 | + | 633 | novel transcript, sense overlapping to CCNB2 |
| AC131212.2 | 10,66 | 2,26 | 2,19 | 12 | 132565071 | 132566425 | + | 1355 | novel transcript |
| AC092902.5 | 10,66 | 2,26 | 2,19 | 3 | 125766516 | 125852936 | + | 4236 | novel transcript |
| AL663058.1 | 10,66 | 2,26 | 2,19 | 1 | 233836080 | 233836432 | - | 353 | acid phosphatase 1, soluble (ACP1) pseudogene |
| LINC01764 | 1,77 | 11,25 | -2,58 | 19 | 15827045 | 15835853 | - | 669 | long intergenic non-protein coding RNA 1764 [Source:HGNC Symbol;Acc:HGNC:52553] |
| MYBPHL | 10,66 | 2,26 | 2,19 | 1 | 109292365 | 109307041 | - | 1789 | myosin binding protein H like [Source:HGNC Symbol;Acc:HGNC:30434] |
| AL136038.5 | 1,77 | 11,25 | -2,58 | 14 | 63642143 | 63642696 | + | 554 | novel transcript |
| AP000344.2 | 10,66 | 2,26 | 2,19 | 22 | 23392553 | 23394792 | + | 1202 | coiled-coil domain containing 188 (CCDC188) pseudogene |
| ARX | 10,66 | 2,26 | 2,19 | X | 25003694 | 25016420 | - | 3097 | aristaless related homeobox [Source:HGNC Symbol;Acc:HGNC:18060] |
| AC022558.1 | 10,66 | 2,26 | 2,19 | 15 | 83022236 | 83024336 | - | 454 | novel transcript, sense intronic to BTBD1 |
| AC006262.4 | 10,66 | 2,26 | 2,19 | 19 | 46249394 | 46250163 | - | 286 | IGF-like family member 4 (IGFL4) pseudogene |
| AL031864.1 | 1,77 | 11,25 | -2,58 | 1 | 171803517 | 171803939 | + | 423 | ribosomal protein S15 (RPS15) pseudogene |
| SEPSECS-AS1 | 92,49 | 46,11 | 1,00 | 4 | 25160641 | 25201440 | + | 4356 | SEPSECS antisense RNA 1 (head to head) [Source:HGNC Symbol;Acc:HGNC:27737] |
| FOXCUT | 41,79 | 18,00 | 1,21 | 6 | 1605531 | 1606079 | + | 333 | FOXC1 upstream transcript [Source:HGNC Symbol;Acc:HGNC:50650] |
| AP000662.1 | 18,67 | 42,73 | -1,19 | 11 | 57638024 | 57652790 | + | 3223 | novel transcript, sense overlapping CLP1 |
| CNGA1 | 9,77 | 26,99 | -1,45 | 4 | 47935977 | 48016672 | - | 3581 | cyclic nucleotide gated channel alpha 1 [Source:HGNC Symbol;Acc:HGNC:2148] |
| RPL37AP1 | 9,77 | 26,99 | -1,45 | 20 | 44466564 | 44466842 | - | 279 | ribosomal protein L37a pseudogene 1 [Source:HGNC Symbol;Acc:HGNC:16548] |
| PCP2 | 10,66 | 26,99 | -1,33 | 19 | 7631611 | 7633748 | - | 799 | Purkinje cell protein 2 [Source:HGNC Symbol;Acc:HGNC:30209] |
| KLHL3 | 71,15 | 33,74 | 1,07 | 5 | 137617500 | 137736090 | - | 11854 | kelch like family member 3 [Source:HGNC Symbol;Acc:HGNC:6354] |
| CPXM1 | 50,69 | 22,50 | 1,17 | 20 | 2794069 | 2800637 | - | 2391 | carboxypeptidase X, M14 family member 1 [Source:HGNC Symbol;Acc:HGNC:15771] |
| CAMK4 | 68,48 | 32,62 | 1,07 | 5 | 111223653 | 111494886 | + | 13910 | calcium/calmodulin dependent protein kinase IV [Source:HGNC Symbol;Acc:HGNC:1464] |
| FMO2 | 32,90 | 69,72 | -1,08 | 1 | 171185208 | 171211230 | + | 4786 | flavin containing monooxygenase 2 [Source:HGNC Symbol;Acc:HGNC:3770] |
| AL118558.3 | 22,23 | 49,48 | -1,15 | 14 | 101948347 | 101949425 | + | 1079 | novel transcript |
| GGTLC4P | 16,00 | 38,24 | -1,25 | 22 | 24248074 | 24249466 | - | 664 | gamma-glutamyltransferase light chain 4 pseudogene [Source:HGNC Symbol;Acc:HGNC:33428] |
| SNORA60 | 3,55 | 13,50 | -1,89 | 20 | 38449370 | 38449503 | + | 134 | small nucleolar RNA, H/ACA box 60 [Source:HGNC Symbol;Acc:HGNC:32654] |
| AL138831.2 | 3,55 | 13,50 | -1,89 | 6 | 4018713 | 4019202 | + | 490 | novel transcript |
| RN7SL521P | 3,55 | 13,50 | -1,89 | 7 | 149125690 | 149125986 | - | 297 | RNA, 7SL, cytoplasmic 521, pseudogene [Source:HGNC Symbol;Acc:HGNC:46537] |
| AL021328.1 | 75,59 | 37,11 | 1,02 | 6 | 5451683 | 5458075 | - | 1333 | uncharacterized LOC101927972 [Source:NCBI gene;Acc:101927972] |
| AC006273.1 | 7,11 | 21,37 | -1,57 | 19 | 781002 | 781862 | - | 861 | novel transcript |
| CYYR1-AS1 | 82,71 | 40,49 | 1,03 | 21 | 26393635 | 26569252 | + | 3713 | cysteine and tyrosine rich 1 antisense RNA 1 [Source:HGNC Symbol;Acc:HGNC:39560] |
| UFSP1 | 26,67 | 56,23 | -1,07 | 7 | 100888723 | 100889718 | - | 996 | UFM1 specific peptidase 1 (inactive) [Source:HGNC Symbol;Acc:HGNC:33821] |
| AC078883.1 | 18,67 | 5,63 | 1,71 | 2 | 172480840 | 172556596 | - | 2447 | novel transcript |
| AC007842.1 | 18,67 | 5,63 | 1,71 | 19 | 40023384 | 40025502 | - | 2039 | protein associated with topoisomerase II homolog 1 (yeast) (PATL1) pseudogene |
| MIR3685 | 18,67 | 5,63 | 1,71 | 12 | 95309923 | 95309984 | + | 62 | microRNA 3685 [Source:HGNC Symbol;Acc:HGNC:38886] |
| AC068189.1 | 18,67 | 5,63 | 1,71 | 8 | 95071732 | 95087924 | - | 794 | novel transcript, antisense to NDUFAF6 |
| AC008083.1 | 18,67 | 5,63 | 1,71 | 12 | 47237734 | 47279021 | - | 663 | novel transcript, antisense to FAM113B |
| CNTNAP3B | 64,92 | 30,37 | 1,09 | 9 | 41890314 | 42129510 | - | 13220 | contactin associated protein like 3B [Source:HGNC Symbol;Acc:HGNC:32035] |
| SLC38A3 | 31,12 | 65,22 | -1,06 | 3 | 50205246 | 50221486 | + | 3968 | solute carrier family 38 member 3 [Source:HGNC Symbol;Acc:HGNC:18044] |
| HOXB9 | 15,11 | 3,38 | 2,12 | 17 | 48621159 | 48626356 | - | 2581 | homeobox B9 [Source:HGNC Symbol;Acc:HGNC:5120] |
| SERPINI1 | 15,11 | 3,38 | 2,12 | 3 | 167735243 | 167825568 | + | 2418 | serpin family I member 1 [Source:HGNC Symbol;Acc:HGNC:8943] |
| AC007216.3 | 15,11 | 3,38 | 2,12 | 16 | 11881075 | 11882569 | - | 1495 | novel transcript, sense intronic to GSPT1 |
| LINC02561 | 15,11 | 3,38 | 2,12 | 10 | 5266033 | 5271236 | - | 692 | long intergenic non-protein coding RNA 2561 [Source:HGNC Symbol;Acc:HGNC:53601] |
| BEGAIN | 15,11 | 3,38 | 2,12 | 14 | 100537147 | 100587413 | - | 7878 | brain enriched guanylate kinase associated [Source:HGNC Symbol;Acc:HGNC:24163] |
| AC138932.3 | 4,44 | 15,75 | -1,80 | 16 | 14988259 | 14990160 | - | 1902 | uncharacterized protein KIAA2013 [Source:NCBI gene;Acc:102724984] |
| AC011458.1 | 15,11 | 3,38 | 2,12 | 19 | 19708965 | 19710465 | + | 617 | ankyrin repeat domain 26 (ANKRD26) pseudogene |
| TMEM238L | 4,44 | 15,75 | -1,80 | 17 | 10794913 | 10804099 | - | 1648 | transmembrane protein 238 like [Source:HGNC Symbol;Acc:HGNC:44356] |
| AC011468.1 | 5,33 | 16,87 | -1,64 | 19 | 52058490 | 52063703 | - | 675 | novel transcript |
| SPDYE18 | 16,89 | 4,51 | 1,88 | 7 | 77052785 | 77060512 | - | 1050 | speedy/RINGO cell cycle regulator family member E18 [Source:HGNC Symbol;Acc:HGNC:51514] |
| RDH5 | 16,89 | 4,51 | 1,88 | 12 | 55720367 | 55724705 | + | 2983 | retinol dehydrogenase 5 [Source:HGNC Symbol;Acc:HGNC:9940] |
| AC106820.4 | 5,33 | 16,87 | -1,64 | 16 | 2476558 | 2482173 | + | 5616 | novel transcript, intronic to TBC1D24 |
| HM13-AS1 | 5,33 | 16,87 | -1,64 | 20 | 31567707 | 31573263 | - | 489 | HM13 antisense RNA 1 [Source:HGNC Symbol;Acc:HGNC:41940] |
| SUMO2P6 | 5,33 | 16,87 | -1,64 | 5 | 174561776 | 174562063 | + | 288 | SUMO2 pseudogene 6 [Source:HGNC Symbol;Acc:HGNC:39016] |
| PCDHA10 | 16,89 | 4,51 | 1,88 | 5 | 140855883 | 141012344 | + | 15224 | protocadherin alpha 10 [Source:HGNC Symbol;Acc:HGNC:8664] |
| AC095055.1 | 5,33 | 16,87 | -1,64 | 4 | 151139991 | 151141103 | + | 1113 | novel transcript, antisense to SH3D19 |
| GP6 | 5,33 | 16,87 | -1,64 | 19 | 55013705 | 55038264 | - | 2703 | glycoprotein VI platelet [Source:HGNC Symbol;Acc:HGNC:14388] |
| TAS1R3 | 80,93 | 39,36 | 1,04 | 1 | 1331314 | 1335306 | + | 3433 | taste 1 receptor member 3 [Source:HGNC Symbol;Acc:HGNC:15661] |
| DDR2 | 20,45 | 44,98 | -1,13 | 1 | 162631373 | 162787400 | + | 10730 | discoidin domain receptor tyrosine kinase 2 [Source:HGNC Symbol;Acc:HGNC:2731] |
| CCDC3 | 88,04 | 43,86 | 1,00 | 10 | 12896625 | 13099652 | - | 3683 | coiled-coil domain containing 3 [Source:HGNC Symbol;Acc:HGNC:23813] |
| ATE1-AS1 | 32,01 | 12,38 | 1,36 | 10 | 121928312 | 121951965 | + | 1172 | ATE1 antisense RNA 1 [Source:HGNC Symbol;Acc:HGNC:49496] |
| AC011290.2 | 32,01 | 12,38 | 1,36 | 7 | 39569376 | 39570198 | - | 823 | thioredoxin domain containing 1 (TXNDC1) pseudogene |
| AC009283.1 | 40,90 | 18,00 | 1,18 | 17 | 39566915 | 39567559 | + | 645 | novel transcript |
| AC022148.1 | 40,90 | 18,00 | 1,18 | 19 | 37545470 | 37549171 | - | 3267 | novel transcript, antisense to ZNF793 |
| AC027373.1 | 40,90 | 18,00 | 1,18 | 8 | 100913247 | 100914388 | - | 1142 | novel transcript, overlapping YWHAZ |
| PNCK | 18,67 | 41,61 | -1,15 | X | 153669730 | 153689010 | - | 5091 | pregnancy up-regulated nonubiquitous CaM kinase [Source:HGNC Symbol;Acc:HGNC:13415] |
| ZNRF2P2 | 49,80 | 22,50 | 1,14 | 7 | 29598795 | 29685255 | - | 887 | zinc and ring finger 2 pseudogene 2 [Source:HGNC Symbol;Acc:HGNC:42793] |
| ASB9 | 81,82 | 40,49 | 1,01 | X | 15235288 | 15270467 | - | 2363 | ankyrin repeat and SOCS box containing 9 [Source:HGNC Symbol;Acc:HGNC:17184] |
