# Supplementary figures and images for: Sulfatide Acts as a Regulatory Molecule Controlling β1 Integrin–STAT5 Signaling and BOLA2-Dependent Apoptotic Pathway in Breast Cancer Cells
Source: Int J Mol Sci. 2025 Dec 9;26(24):11873. doi: 10.3390/ijms262411873 (PMC12733076; doi:10.3390/ijms262411873)

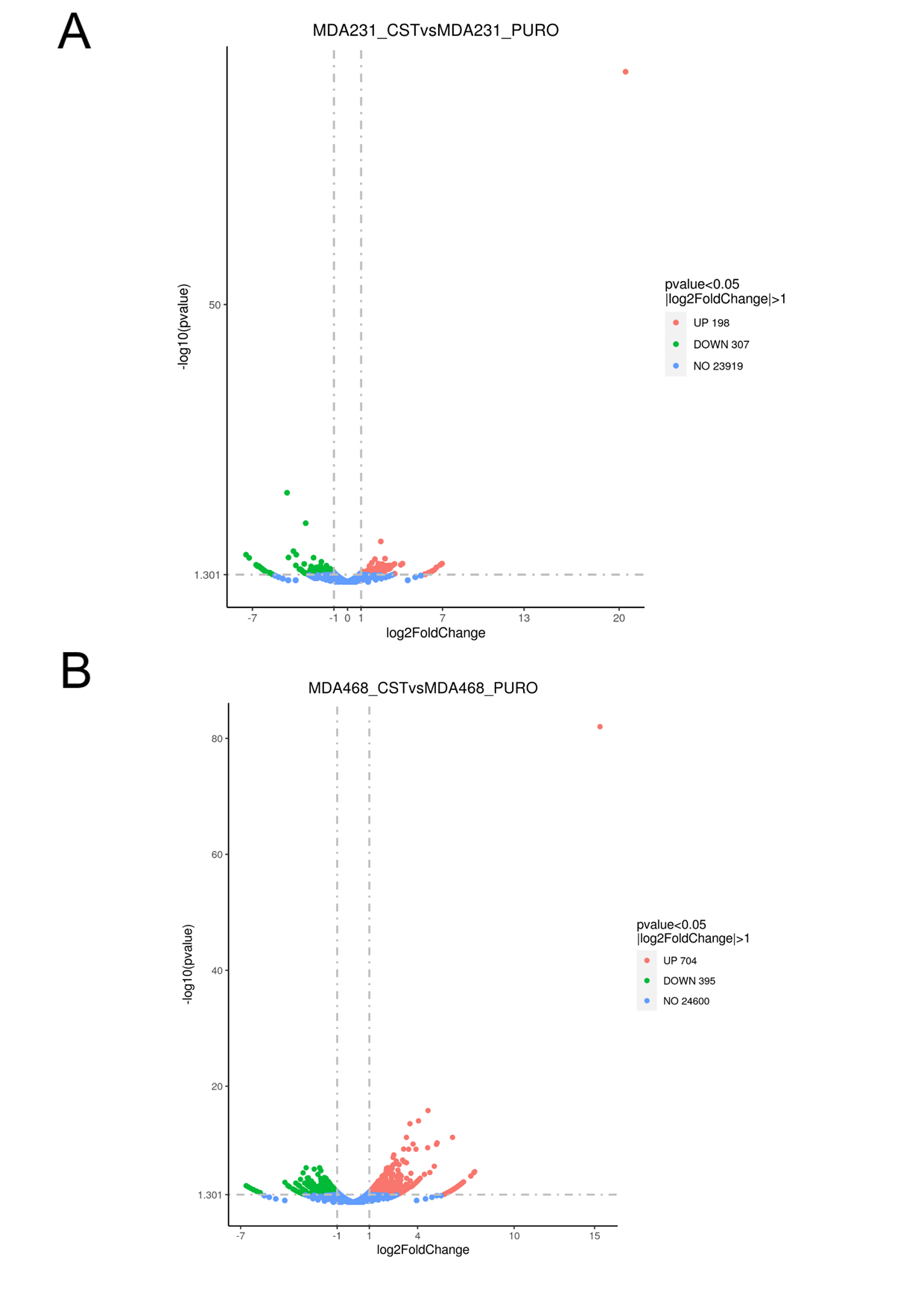

Supplement: Supplementary file 1 [file ijms-26-11873-s001.zip › Fig S1.tif]

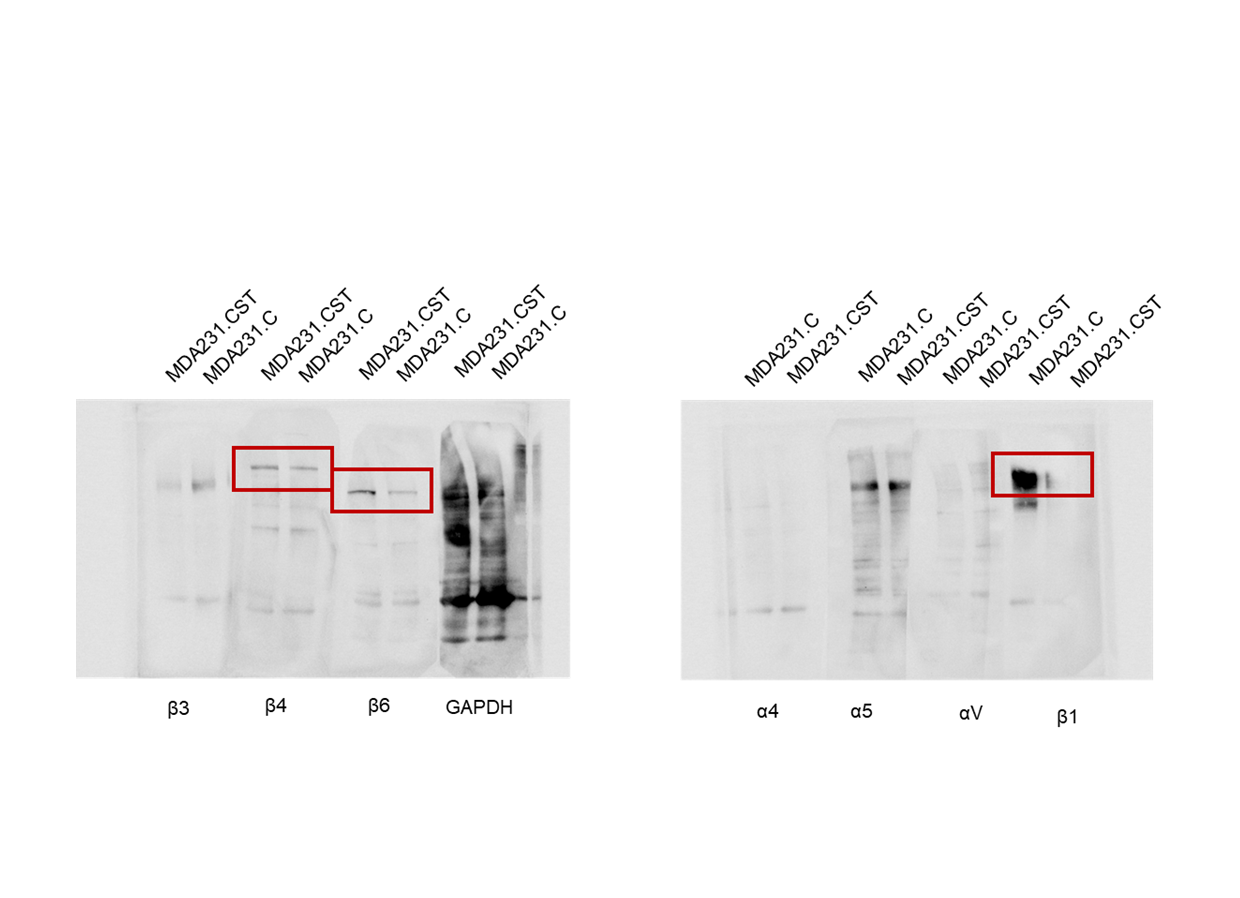

Supplement: Supplementary file 1 [file ijms-26-11873-s001.zip › Fig S2.tif]

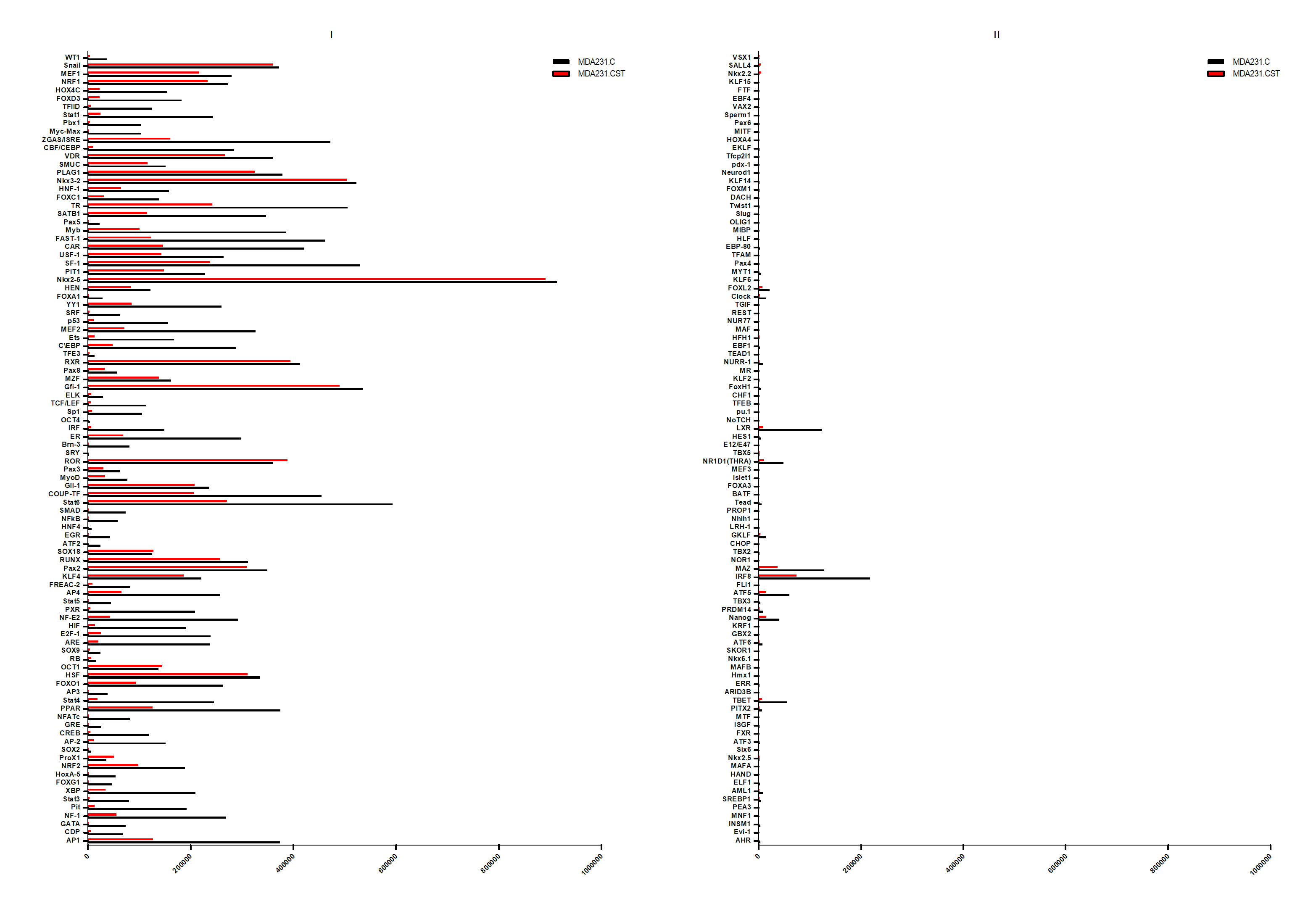

Supplement: Supplementary file 1 [file ijms-26-11873-s001.zip › Fig S3.tif]

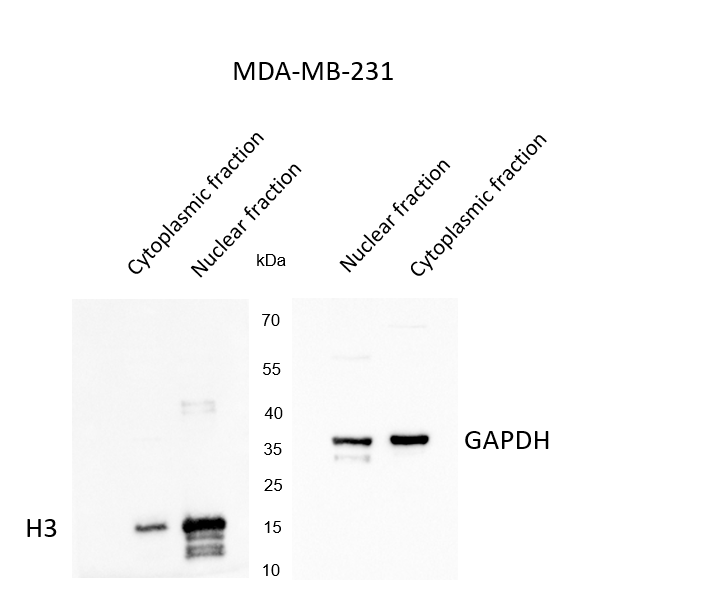

Supplement: Supplementary file 1 [file ijms-26-11873-s001.zip › Fig S4.tif]
